# A New Isolation with Migration Model along Complete Genomes Infers Very Different Divergence Processes among Closely Related Great Ape Species

**DOI:** 10.1371/journal.pgen.1003125

**Published:** 2012-12-20

**Authors:** Thomas Mailund, Anders E. Halager, Michael Westergaard, Julien Y. Dutheil, Kasper Munch, Lars N. Andersen, Gerton Lunter, Kay Prüfer, Aylwyn Scally, Asger Hobolth, Mikkel H. Schierup

**Affiliations:** 1 Bioinformatics Research Center, Aarhus University, Aarhus, Denmark; 2 Department of Computer Science, Aarhus University, Aarhus, Denmark; 3 Institute of Evolutionary Sciences, University of Montpellier 2, Montpellier, France; 4 The Wellcome Trust Centre for Human Genetics, Oxford, United Kingdom; 5 Max Planck Institute for Evolutionary Anthropology, Leipzig, Germany; 6 The Wellcome Trust Sanger Institute, Cambridge, United Kingdom; 7 Department of Biology, Aarhus University, Aarhus, Denmark; Fred Hutchinson Cancer Research Center, United States of America

## Abstract

We present a hidden Markov model (HMM) for inferring gradual isolation between two populations during speciation, modelled as a time interval with restricted gene flow. The HMM describes the history of adjacent nucleotides in two genomic sequences, such that the nucleotides can be separated by recombination, can migrate between populations, or can coalesce at variable time points, all dependent on the parameters of the model, which are the effective population sizes, splitting times, recombination rate, and migration rate. We show by extensive simulations that the HMM can accurately infer all parameters except the recombination rate, which is biased downwards. Inference is robust to variation in the mutation rate and the recombination rate over the sequence and also robust to unknown phase of genomes unless they are very closely related. We provide a test for whether divergence is gradual or instantaneous, and we apply the model to three key divergence processes in great apes: (a) the bonobo and common chimpanzee, (b) the eastern and western gorilla, and (c) the Sumatran and Bornean orang-utan. We find that the bonobo and chimpanzee appear to have undergone a clear split, whereas the divergence processes of the gorilla and orang-utan species occurred over several hundred thousands years with gene flow stopping quite recently. We also apply the model to the *Homo*/*Pan* speciation event and find that the most likely scenario involves an extended period of gene flow during speciation.

## Author Summary

Next-generation sequencing technology has enabled the generation of whole-genome data for many closely related species. For population genetic inference we have sequenced many loci, but only in a few individuals. We present a new method that allows inference of the divergence process based on two closely related genomes, modelled as gradual isolation in an isolation with migration model. This allows estimation of the initial time of restricted gene flow, the cessation of gene flow, as well as the population sizes, migration rates, and recombination rates. We show by simulations that the parameter estimation is accurate with genome-wide data and use the model to disentangle the divergence processes among three sets of closely related great ape species: bonobo/chimpanzee, eastern/western gorillas, and Sumatran/Bornean orang-utans. We find allopatric speciation for bonobo and chimpanzee and non-allopatric speciation for the gorillas and orang-utans. We also consider the split between humans and chimpanzees/bonobos and find evidence for non-allopatric speciation, similar to that within gorillas and orang-utans.

## Introduction

The decreasing cost of genome sequencing has led to the sequencing of many species, including closely related species. With these available genomes, it becomes possible to study the speciation process in more detail. Due to the processes of recombination and coalescence, the complete genomes of two related species contain a large number of partly independent histories, with different regions having different migration histories and times to common ancestry. These histories, if inferred, can inform us about key parameters of the species divergence process. Particularly informative is the distribution of the length of genomic fragments having identical histories, since this depends directly on the time interval over which recombination could have acted and therefore on the distribution of coalescent times. Demographic parameters shape this distribution, so inference of it is informative about demographic history. However the difficulty of modelling coalescence with recombination means that most previous approaches have been unable to exploit this information, with one recent notable exception being inference of population size history from a single diploid genome [1].

Previous isolation with migration (IM) models have been designed to deal with relatively short sequences from several individuals of each species, since this was typical of data sets available until recently. Adding more individuals to a data set is often not as powerful as adding loci, since most coalescence events occur recently in the history of the samples and there are only few ancestors present at deep coalescence times. For the constant population size coalescent, the total branch length in the ancestry of a sample set grows logarithmically with the number of samples, but linearly in the number of loci, and most statistical methods for exploring the evolution of closely related species therefore employ multiple loci with small sample sizes [2]–[7]. These methods, however, typically assume loci are sufficiently short and widely separated that recombination is negligible within them and occurs freely between them. One notable exception is the MIMAR model, which does allow recombination within loci but still assumes free recombination between loci, see Becquet and Przeworski (2007) [8].

Using coalescent theory it is reasonably straightforward to compute the coalescence density under many demographic scenarios, including scenarios with and without gene flow. This follows from the Markov property of the process when viewed backwards in time. When recombination is absent, we can often derive simple maximum likelihood algorithms for inferring parameters. When recombination *is* present, however, the likelihood computations quickly become computationally infeasible since the process is not Markovian across loci [9]. Reasonable approximations can be made, however, by *assuming* the Markov property across loci [10]–[12]. In order to fully model complete recombining genomes we have developed a class of models that we term “coalescent hidden Markov models” or CoalHMMs. These models are based on the sequential Markov coalescence approach [10], [11] which models the coalescent process as a Markov process along a genome alignment. The coalescence states, however, are hidden and can only be inferred by comparing the sequences.

CoalHMMs permit recombination between any neighbouring pair of nucleotides, and represent the correlation between sites as a Markov model along the alignment [1], [13]–[16]. In Hobolth et al. (2007) [13] and Dutheil et al. (2009) [14] we analysed alignments of three genomes (human, chimpanzee and gorilla) using a Markov model with four states, in which one state corresponds to a genealogy consistent with the species phylogeny, and with the two most closely related species coalescing recently, and the other states correspond to the three possible genealogies with a deep initial coalescence (further back in time than the deepest speciation). The same model was used to analyse the human, chimpanzee and orang-utan in Hobolth et al. (2011) [17]. In Mailund et al. (2011) [15], on the other hand, the model had a variable number of states corresponding to different coalescence times in an alignment of two orang-utan genomes. In all models, speciation was modelled as a simple isolation model, with panmictic mating before the speciation event and no gene flow following speciation.

**Figure 1:**
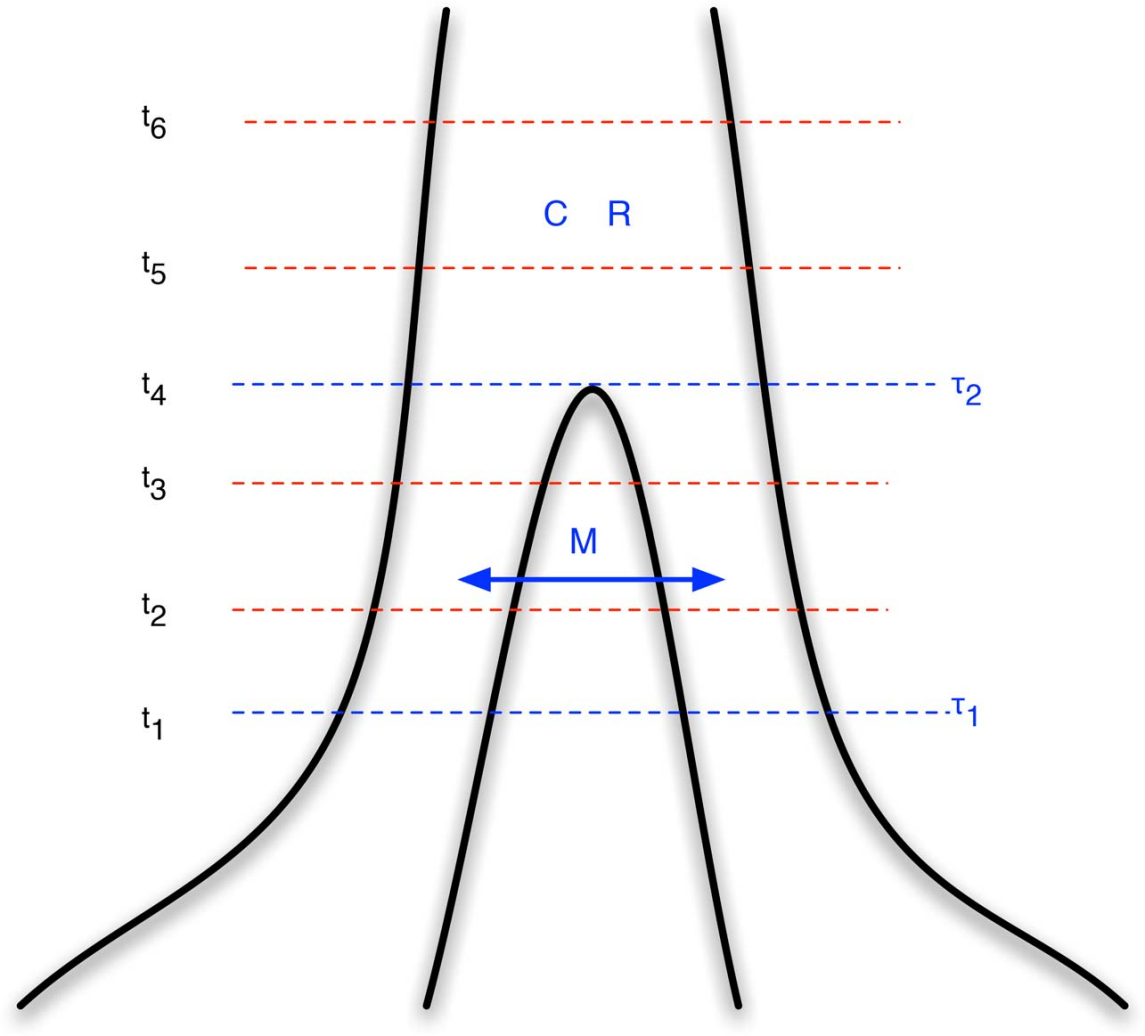
Isolation-with-migration model. Our isolation-with-migration model considers two separated populations (sub-species or species) derived from a shared ancestral population in the recent past. The model assumes that the ancestral population split into two populations in the past, at time 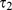, and that these two populations exchanged genes with migration rate 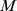 until a later time, 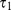, where gene flow stopped. The coalescence process in this model is parameterized with a coalescence rate (inverse of the effective population size), 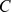, and a recombination rate, 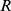. The model is translated into a finite-state hidden Markov model by discretizing time into time intervals with break points 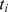.

In this paper we extend the coalescent hidden Markov model of Mailund et al. (2011) [15] to an IM model, where we allow limited gene flow after an initial population split, followed by a period with no gene flow (see Figure 1). We derive the transition probabilities for the Markov model from finite state continuous time Markov chains parameterized by the split times and the rates of coalescence, recombination and migration. With this approach we exactly compute the transition probabilities between divergence times for pairs of nucleotides according to the coalescent process with recombination, and by assuming a Markov dependency along the alignment we obtain an approximation to the process that is computationally efficient for scanning whole genome data. We apply the approach to data from three pairs of recently diverged great ape species: the two orang-utan species, the eastern and western gorilla species, and chimpanzees and bonobos. We also apply it to the more ancient divergence between humans and the *Pan* genus (chimpanzees and bonobos).

## Results

### Key aspects of the isolation-with-migration coalescent hidden Markov model

When considering only pairs of genomes, the likelihood of a model will depend only on the divergence time at each locus [1], [3], [15]. By computing the joint coalescence time density for pairs of nucleotides we can compute a density for the coalescent time of the right nucleotide of a pair, conditional on when the left nucleotide coalesces. By assuming the Markov property along an alignment we can then efficiently compute the coalescence density for each nucleotide along a pairwise genome alignment.

In Mailund et al. (2011) [15] we used this observation to compute the joint coalescence density of pairs of nucleotides in a simple isolation model from a continuous time Markov chain (CTMC). In this paper, we take the same approach, but we now compute the joint coalescence density of pairs of nucleotides in a scenario which includes a period of restricted migration. In this setting, the state space of the CTMC explodes in the number of states. Constructing the CTMC manually thus becomes tedious and error prone, and instead we have implemented an algorithm for constructing the rate matrix (see Methods).

Once the joint coalescence densities are computed however, we can construct a hidden Markov model from following the approach of our previous work. We discretize time in a number of intervals (see Figure 1) to get a finite state space for the hidden Markov model. In all analyses we used 20 intervals, 10 in the migration period and 10 in the ancestral population; see Text S1 for results for different numbers of time intervals. From the joint coalescence densities we can compute the probability of the left nucleotide of a pair coalescing in one time interval and the right nucleotide in another, and from this obtain the transition probability matrix of the hidden Markov model. We compute the mean coalescence time in each time interval to get the emission probabilities of the hidden Markov model, assuming that coalescence occurred at that time point. Once we have computed the transition and emission probability matrices for the hidden Markov model, we can use well known hidden Markov model algorithms to compute the likelihood of a genome alignment.

The parameters of the hidden Markov model are the same as those used for the CTMC for computing the coalescence densities. The IM model introduced in this paper is parameterized by *i)* the coalescence rate of lineages, *ii)* the recombination rate between pairs of nucleotides, and *iii)* the rate of migration between populations. We scale all these rates by number of substitutions, such that the coalescence densities are measured in the expected number of substitutions, which simplify the likelihood computations.

We then assume three different time periods (see Figure 1). The period from the present until time 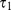 allows coalescence and recombination events within populations, but no migration events between them. From time 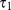 to time 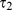, migration events are allowed as well coalescence and recombination events, and further back in time than 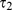 we assume a panmictic mating, where again coalescence and recombination events are allowed.

We assume that both the recombination rate 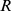 and coalescence rate 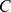 are constant across lineages and over time. We also assume that the migration rate 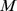 is symmetric between the two populations. The mathematical framework used to construct the model does allow us to vary all rate parameters both in time and along the sequence, and allows the migration rate to be asymmetric between populations, but due to identifiability issues (see Text S1, Sections 4 and 5, and [7]) we restrict ourselves to symmetric parameters.

### Performance of the model

The coalescent HMM employs two important approximations. It assumes that the coalescent process is Markov along the sequences and that coalescent events occur at discrete time points rather than continuously in time. The Markov assumption is very difficult to relax because it enables us to reduce the problem of inference across the genome to that of the history of two adjacent nucleotides. The assumption of discrete coalescent times can be investigated by varying the number of intervals. Therefore, we have used extensive simulation studies to validate that the model can recover true parameters simulated under the coalescent with recombination process, both under ideal circumstances and under circumstances where different aspects of the model are mis-specified (see Text S1). Simulations are carried out under the more complex coalescent with recombination and migration model, whereas inference uses the assumptions of Markov property and discrete coalescence times.

For all simulations we used a coalescence rate of 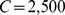 – corresponding to an effective population size 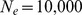 assuming a substitution rate of 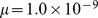 substitutions per bp per year and 20 years per generation – and a recombination rate of 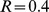 – corresponding to 0.8 cM/Mb with the assumed mutation rate and generation time. To explore different scenarios we simulated 10 independent data sets for each combination of parameters 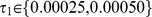 (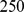 and 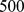 thousand years ago with the assumed mutation rate), 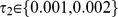 (1 and 2 million years ago), and 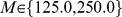 (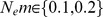). All simulation results are based on 10 Mbp of data (but see Text S1, Section 3.3 for accuracy as a function of data size). We present a broad range of analysis of the simulations in the supplement and will here only focus on a couple of key aspects.

**Figure 2:**
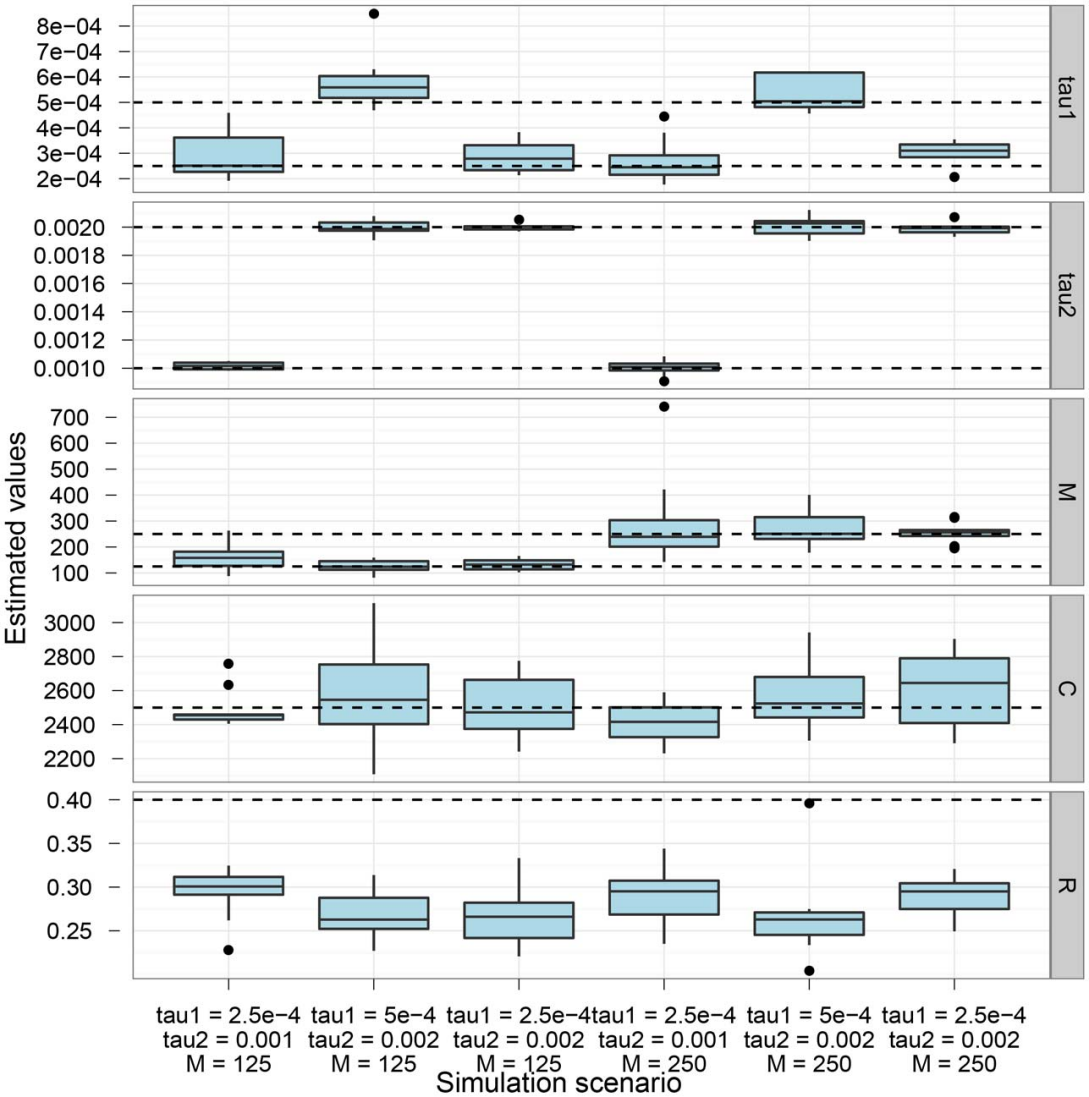
Estimation accuracy. The box-plot shows the distribution of parameter estimates for six different simulation scenarios. In all scenarios the coalescence rate and the recombination rate parameters are kept fixed, while the end of gene flow, 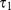, the initial population split, 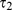, and the migration rate, 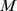, varies between scenarios. For each simulation scenario, 10 independent data sets were generated and analyzed. The dashed horizontal lines indicate the simulated values for the five parameters. The recombination rate is consistently under-estimated while the remaining parameters are well recovered.

Figure 2 shows the parameter estimation accuracy for the six different scenarios simulated. As shown, the parameters are generally well recovered except for the recombination rate that is consistently under-estimated.

**Figure 3:**
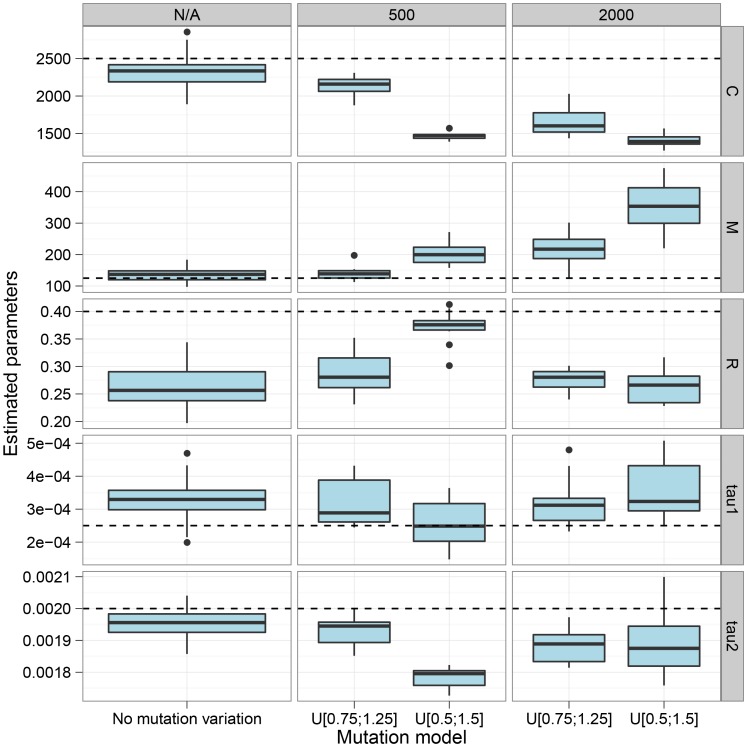
The effect of mutation rate variation. The figure shows the effect on parameter estimation when the mutation rate is varied along the genome alignment. We split the alignment into segments geometrically distributed with mean length 500 bp and 2 kbp, and the mutation rate is then scaled by a random value chosen uniformly in the range 0.75 to 1.25 or 0.5 to 1.5. The dashed lines show the simulated values. The largest effect on varying the mutation rate is seen in the top-most parameters, the coalescence rate and the mutation rate. Varying the mutation rate increases the variance in coalescence times scaled with mutation rate, which is interpreted by the model as a decreased coalescence rate, while segments with low mutation rates are seen as more recent coalescence rates which the model interprets as evidence for migration. Consequently, variation in mutation rate decreases our estimates of the coalescence rate and increases our estimates of migration rates.

The CoalHMM assumes that both the mutation rate and the recombination rate are constant in the region analysed, which in general will not be true. We explored the sensitivity to variation in rates through simulations. Figure 3 shows the effect of varying the mutation rate in segments along the alignment, in blocks of length geometrically distributed with mean either 500 or 2000 bp, and varying the mutation rate by a factor uniformly distributed in either range 0.75–1.25 or 0.5–1.5. The main effect of varying mutation rate appears to be a decrease in the estimated coalescence rate and an increase in the estimated migration rate. The decrease in coalescence rate is explained by a greater variance in estimated coalescence times when mutation rate variation is added to the variation in actual coalescence times. The model misinterpretes stretches of the genome with small divergence as recent coalescence times causes the increase in migration rate estimates.

**Figure 4:**
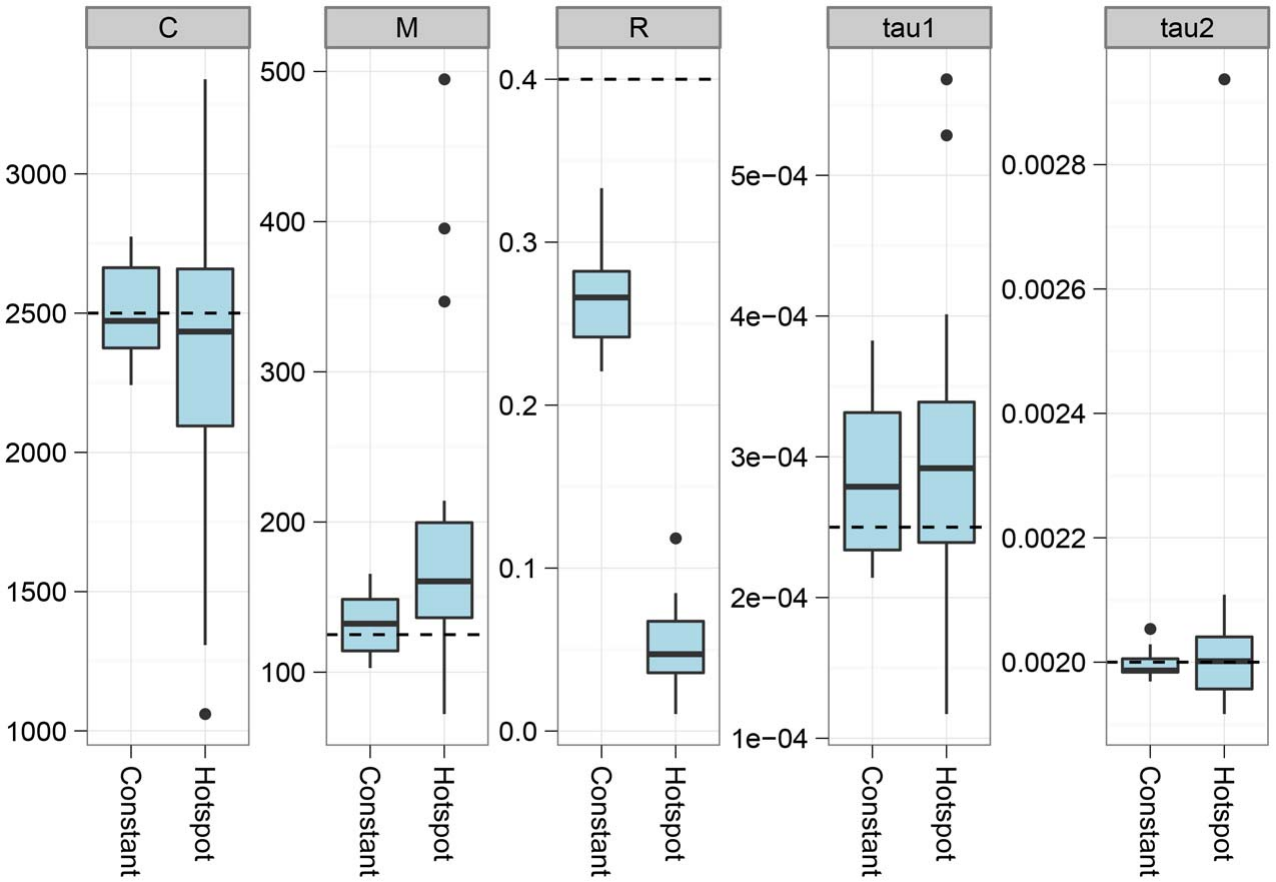
The effect of recombination rate variation. The figure shows the effect on parameter estimation when the recombination rate is varied along the genome alignment. To simulate variation in the recombination rate, we sampled random 10 Mbp segments of the human genome, extracted the DeCODE recombination map for these segments, and scaled the recombination rate in the simulations according to the variation in this map. The dashed lines show the simulated values of the parameters. For most parameters, the effect of varying the recombination rate is seen as an increased variance in the estimates, while they do not appear to be biased. The exception is the recombination rate that becomes even more underestimated than for a constant recombination rate.

Figure 4 shows estimation results when the recombination rate along the alignment is taken from the DeCODE recombination map [18]. Introducing variation in the recombination rate does not appear to bias the parameters, but the variance in estimates generally increases.

**Figure 5:**
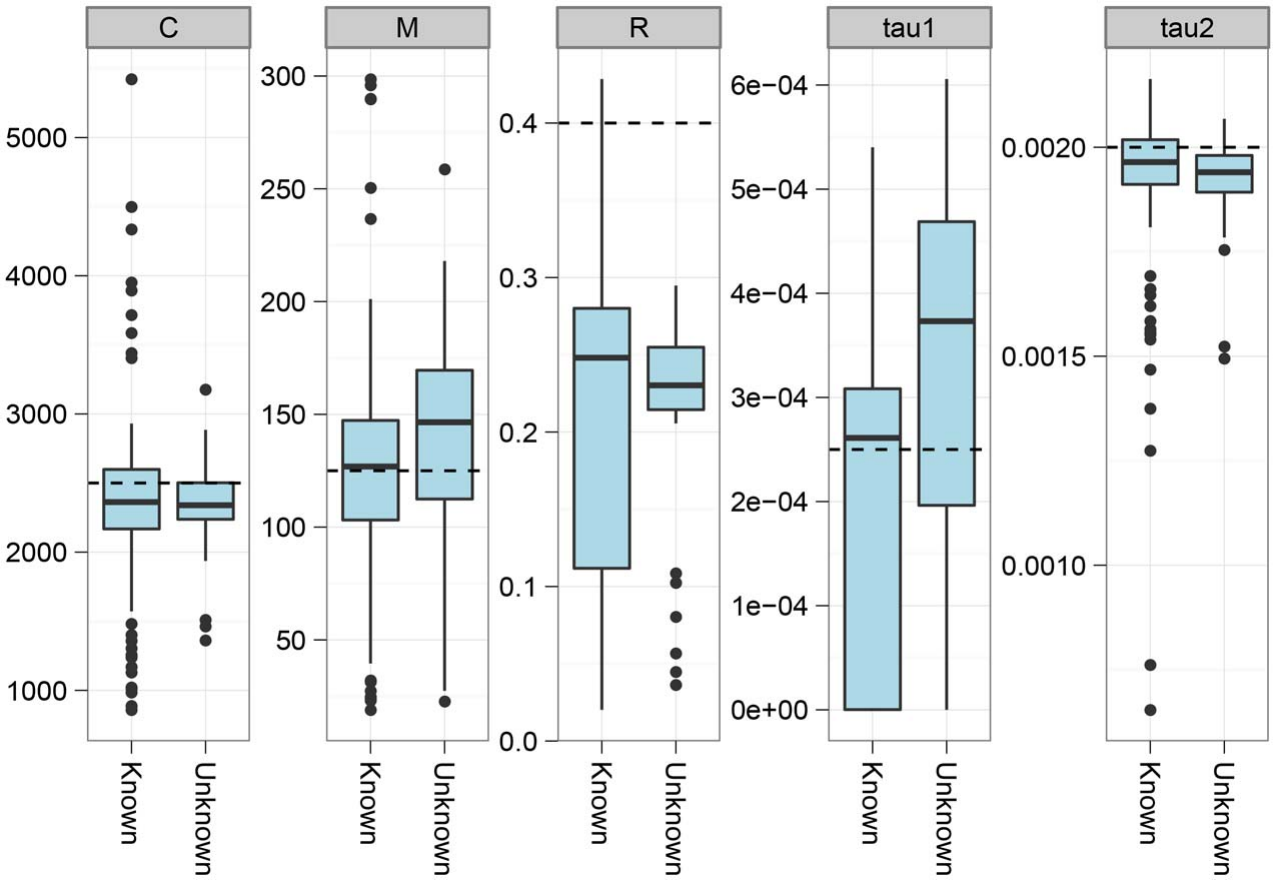
The effect of using a random genotype phase. We simulated the situation where the genotype phase is unknown by simulating two genomes and selecting a random allele for all heterozygotic sites. The plot shows the effect on parameter estimates of not knowing the phase.

The model assumes that we have one haploid genome from each species. From sequencing data, however, we generally only obtain diploid genomes, and inferring the phase to split this into haploid genomes is not immediately possible with only one genome sequenced. If the species are sufficiently diverged, however, most polymorphism in the species will be local to one of the species, and which variant is considered for a heterozygotic site will not matter for the divergence to the other species. However, when species are so closely related that shared polymorphisms are common, we expect that assuming phased chromosome will make us believe that more recombination has occurred and therefore bias the inferred 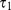 upwards. To test this we simulated two genomes per species and compared parameter estimates on haploid genomes and mosaic genomes constructed by taking a random allele at all positions where the two sequences in a species differed. As shown in Figure 5 and in the supplement, species have to have diverged very recently (within the last 250,000 years) for this effect to be detectable.

### Analysis of closely related great ape species

Recently-sequenced primate genomes allow us to apply the model on three different closely related species pairs: (a) bonobos and chimpanzees (Prüfer et al. (2012) [19]), (b) eastern and western gorillas (Scally et al. (2012) [20]), and (c) Sumatran and Bornean orang-utans (Locke et al. (2011) [21]). We analysed these species pairs in 10 Mbp intervals using both the IM model from the present study and the isolation model (I model) from Mailund et al. (2011) [15], in order to test whether a period of limited gene flow explains the data better than a clean split. Estimates for each 10 Mbp segment can be seen in Dataset S1.

**Figure 6:**
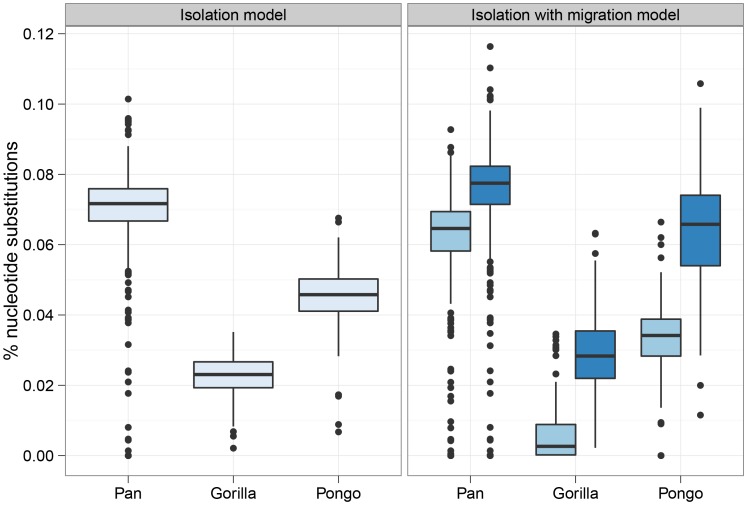
Split times estimates for the three great ape genera. The box plot shows the estimated split times using either the isolation model or the isolation-with-migration model for the three great ape comparisons. The box plots on the left shows the split time estimate in the isolation model while the box plots on the right shows both the initial population divergence and the end of gene flow. The variation in estimates is from each 10 Mbp segment of the genome.

Figure 6 shows the divergence times estimated under the I model (a single divergence time) and the IM model (two divergence times). In each case the I model estimates a time intermediate to the two divergence times of the IM model. The median migration rates per coalescence (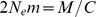) are 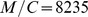 for bonobo and chimpanzee, 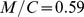 for eastern and western gorilla and 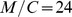 for Sumatran and Bornean orang-utan. Thus, the short migration epoch for bonobo and chimpanzee appears virtually panmictic while the epoch for the gorillas has very limited migration.

**Figure 7:**
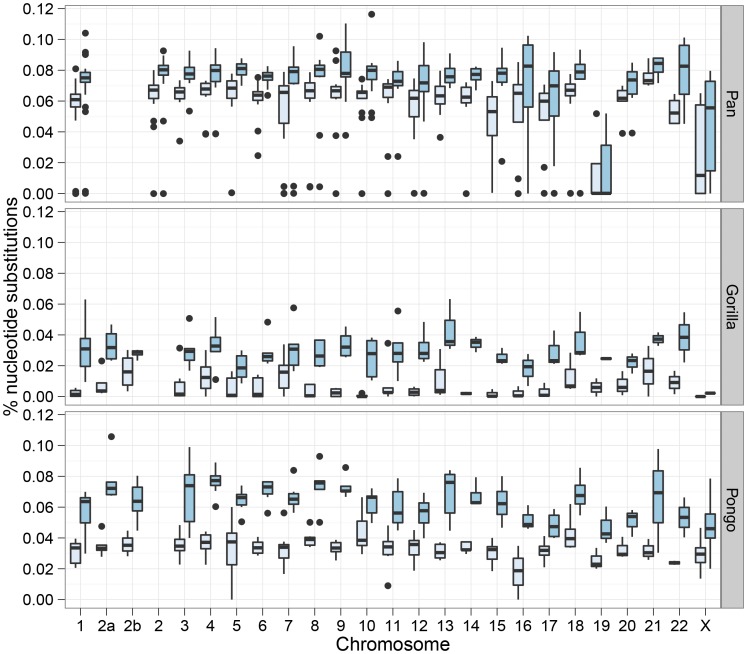
Chromosome wise split time estimates. The box plots show the estimates of the initial split time and the end of gene flow in the isolation-with-migration model for each 10 Mbp segment for each chromosome.

**Figure 8:**
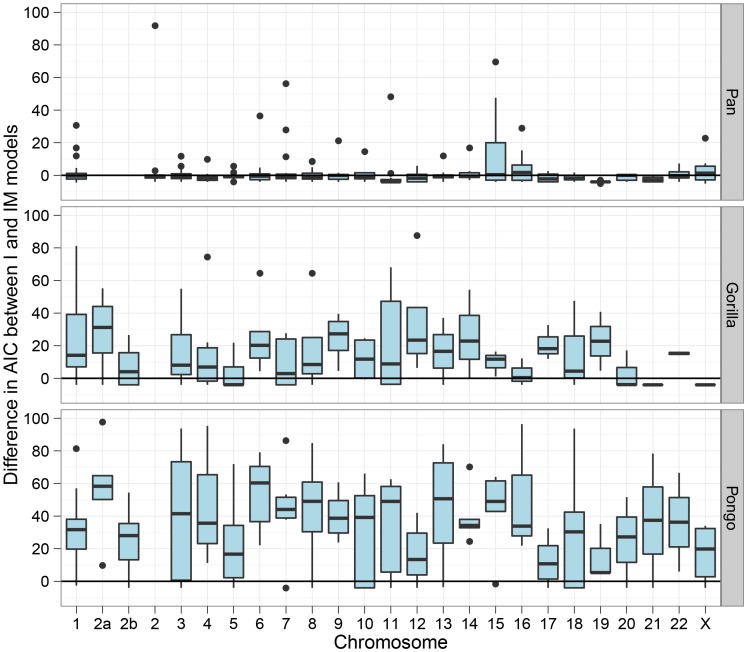
Model comparison between the isolation and the isolation-with-migration model. The box plots show the Akaike Information Criteria (AIC) for the isolation model against the isolation-with-migration model. For each 10 Mbp genomic segment we have plotted the AIC for the model including migration minus the model without. The model with the smallest AIC should be preferred, so values below zero prefers the isolation model while values above zero prefers the migration model.

Figure 7 shows the divergence times for each chromosome and Figure 8 shows a comparison of the I and IM models for each chromosome in the three species pairs (for details of the model checking approach see Text S1, Section 8). As expected from the short time interval of gene flow and the large amount of gene flow estimated, the IM model does not provide a better fit to the data than the I model for the *Pan* comparison. For gorillas and orang-utans, however, the IM model is preferred, with the strongest support for the IM model in gorillas. We conclude that the *Pan* split is consistent with allopatry, whereas both the orang-utans and gorillas split non-allopatrically, and the split between gorillas much more recent than that between orang-utans.

### Human and chimpanzee speciation

**Figure 9:**
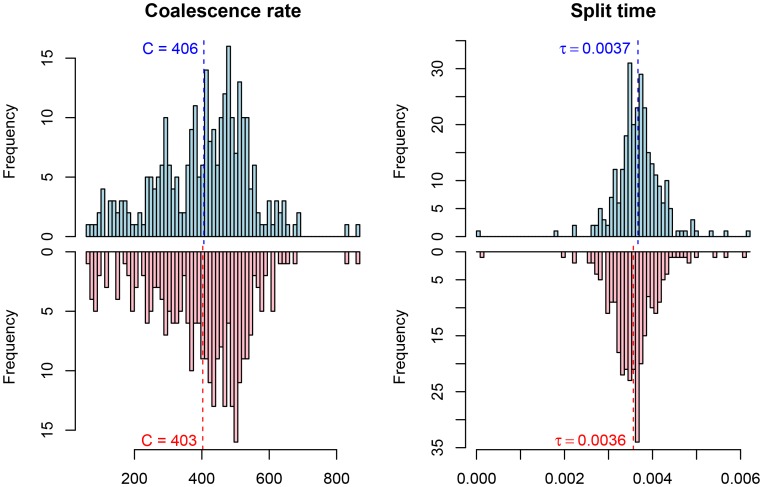
Parameter estimates for the human/chimpanzee split with the isolation model. The histograms show the distribution of parameter estimates for the human/bonobo speciation (blue) and the human/chimpanzee speciation (red) using the isolation model.

**Figure 10:**
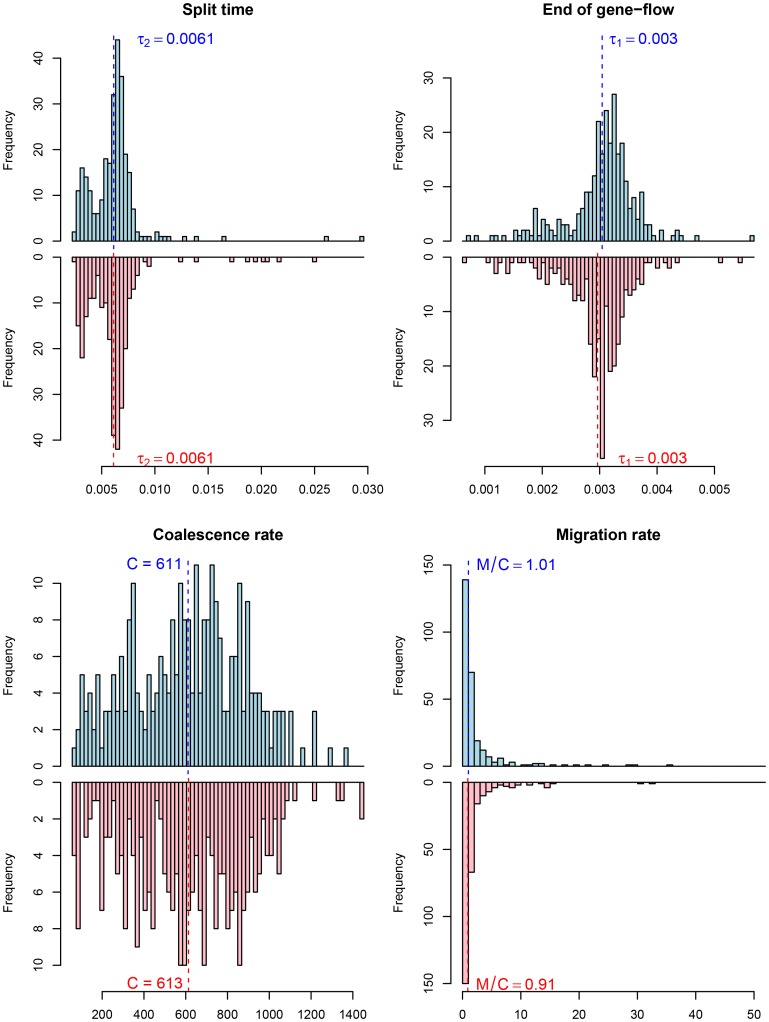
Parameter estimates for the human/chimpanzee split with the isolation-with-migration model. The histograms show the distribution of parameter estimates for the human/bonobo speciation (blue) and the human/chimpanzee speciation (red) using the isolation-with-migration model.

**Figure 11:**
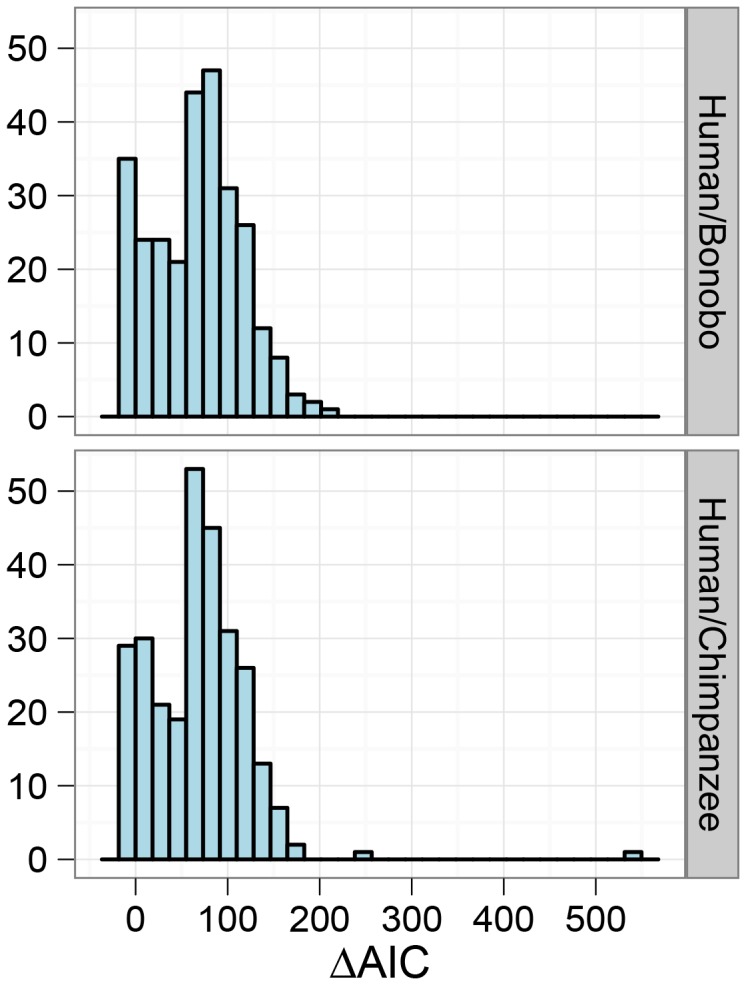
Model comparison for the human/chimpanzee and the human/bonobo split. The histograms show the distribution of AIC differences for the isolation and isolation-with-migration model for the human/chimpanzee comparison and the human/bonobo comparison. Negative values indicate a preference for the isolation model while positive values indicate a preference for the isolation-with-migration model. The overall result points toward a preference for a prolonged speciation for the Homo/Pan split.

Among the great ape speciation events, the human and chimpanzee speciation has received the most attention. We applied our new model to this speciation event using both the chimpanzee and bonobo genomes compared to the human genome, see Figure 9 and Figure 10. Estimating parameters using the isolation model, we see a recent speciation with a large ancestral population size (i.e. small coalescence rate), while estimating parameters using the isolation with migration model we find a relatively large interval with gene flow and a smaller ancestral effective population size, although still large compared to most extant great apes. Comparing the two models using the AIC approach, we find that the isolation with migration model is preferred, suggesting that the *H*omo/*P*an split was not allopatric (see Figure 11).

## Discussion

### A new isolation with migration model

Our study shows that detailed information on the divergence process can be gathered from just two related haploid genomes through joint inference of the length of segments with the same history and their times to coalescence. A period of time with limited migration is detectable as a period in which coalescences occur at a much lower frequency than in a panmictic population, because they are limited by the rate of migration events. Simulations under the full coalescence with recombination process show that the Markov assumption does not significantly bias the estimation of parameters, except for the recombination rate which is consistently underestimated, typically by 20–30%. The cause of this underestimation is the tendency of the real coalescent with recombination process to return to the same ancestor which implies that the average effect of recombination events is smaller than assumed by the Markov model (for an extended discussion, see Dutheil *et al.* (2009) [14] and Mailund *et al.* (2011) [15, Text S1 Section 1.4.3]).

Estimation of time and migration parameters is robust to typical violations of model assumptions such as mutation rate and recombination rate variation. A practical concern is that the model assumes haploid phased genomes whereas most genome sequences are a random mix of two haplotypes. Using a random phase should have no effect if the genomes are sufficiently diverged that they do not share any polymorphism; in this case the patterns of coalescence time will be the same for either phase at any position along the genome. If the genomes have shared polymorphism, then the phase will affect the estimated coalescence time, and with a high degree of shared polymorphism we expect that a random phase will cause the HMM to jump between states too often.

To investigate the consequences of this, we simulated diploid genomes and constructed haploid genomes by choosing a random phase, and then estimated parameters from this data (see Text S1, Section 7). We found that split times were very slightly overestimated when a random phase was used, while the recombination rate was underestimated by a somewhat greater extent and the coalescence rate could be overestimated by a factor of up to 50%. As expected however, the biases introduced by using a random phase quickly disappear when the genome divergence increases.

### Scaling times and effective population sizes

The demographic parameters inferred by our model are expressed in units of sequence divergence (substitutions per base pair). To translate these into units measured in years and effective population sizes measured in individuals requires both a genomic substitution rate and a generation time. A substitution rate has typically been estimated from fossil dates, with values around 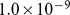 per base pair per year as typical for great apes.

**Figure 12:**
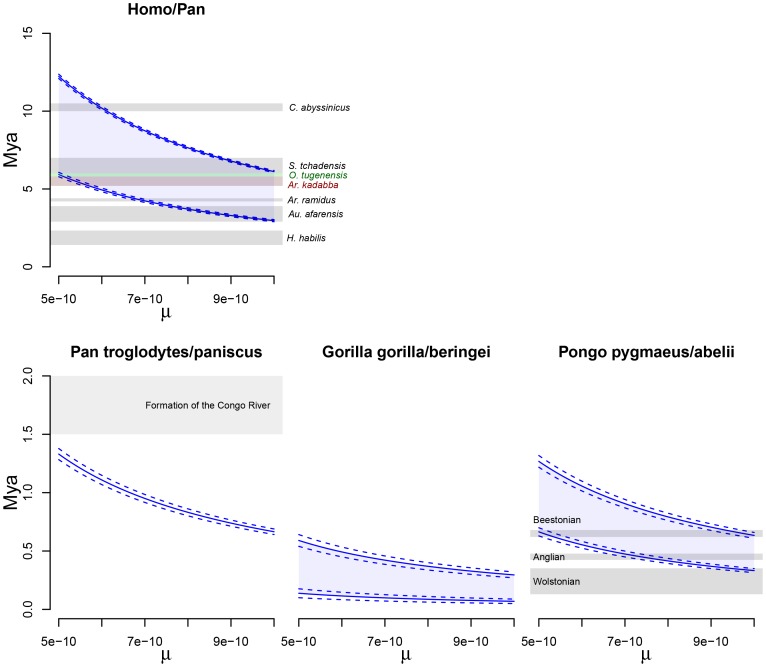
Split times scaled in years. The figure shows the inferred split times when scaled with a mutation rate, 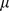, ranging from 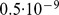 to 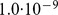. The solid lines show mean estimates while the dashed lines the 95% confidence interval (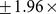
SEM). The Homo/Pan slit is annotated with key fossils, the chimpanzee/bonobo split with the formation of the Congo River, and the orang-utan split with glacial period where sea level was low and migration between orang-utans possible.

Recent measurements of *de novo* mutations in modern humans, however, combined with studies of the generation time in humans and African apes, have revealed a mutation rate of around 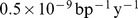 [22]–[24]. This rate is significantly lower than estimates based on fossil calibration. However there are constraints on how far back this can be extrapolated, given fossil evidence for earlier evolutionary events (for example the divergence of orang-utan from other apes seems incompatible with dates older than 15–20 Mya) [20]. It may also be that the per-generation rate differs in other apes. For these reasons, in Figure 12 we show how the estimated timescales for the speciation processes investigated depend on the mutation rate assumed. For comparison with previous analyses, we show similar plots annotated with mutation rates and time estimates in other studies in Text S2, Section 3.

### Speciation processes within the non-human great apes

Our application of the model to three closely related great ape species pairs revealed different speciation processes between chimpanzees and bonobos on one hand and the gorilla and orang-utan species on the other.

We estimate that the two gorilla species have experienced a long period of time with a very small amount of gene flow. Evidence for recent gene flow between these species was also found by Thalmann et al. (2007) [25] and by Scally *et al.* (2012) [20]. Thalmann *et al.* (2007) [25], using a mutation rate just below 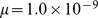, estimated an initial population divergence 0.9 Mya to 1.6 Mya, with continued gene flow ceasing 80 kya to 200 kya. Under the same scaling we estimate a much more recent population split, later than 0.5 Mya, with gene flow continuing until quite recently. Scally et al. (2012) [20] also presented evidence for recent gene flow, but using a model that assumed initial divergence followed by gene flow continuing to the present day, and with 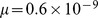 found a much more recent divergence time corresponding to 300 kya with their scaling (see Text S2, Figure 8).

For the two orang-utan species we also find evidence for an extended period of limited gene flow but with a more ancient cessation of gene flow than is observed for the Gorillas. This is in agreement with the DaDi analysis presented by Locke et al. (2011) [21] which also posits a gradual divergence process. Other studies estimated much more ancient divergence times; for example Steiper (2006) [26] estimated a divergence time between 3 and 5 Mya and Becquet and Przeworski (2007) [8] a divergence around 1.4 Mya (although with a wide confidence interval that overlaps other estimates) using also 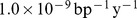 (see Text S2, Figure 9). Our inference falls within these extremes, with the 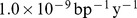 scaling we estimate initial divergence almost 600 kya and had an moderate level of gene flow over a period of 300 thousand years.

Finally, for the chimpanzees and bonobos we find no evidence against an allopatric separation. This is in agreement with Won and Hey (2005) [27] and Hey (2010) [28], who also used an IM model to study the separation between these two species. Indeed, the chimpanzee-bonobo speciation process has previously been suggested as an example of allopatric speciation in which the Congo River acted as a barrier to gene flow [29], since the present-day ranges of bonobos and chimpanzees are separated by that river. Fluvial drainage patterns in Central Africa may well have changed substantially in response to geological and climatological events over the last 20 million years, and could have triggered speciation. However the formation of the Congo River itself may have occurred substantially more than 2 million years ago [30], in which case it would predate most estimated divergence times for chimpanzee and bonobo, including those presented here. Prüfer er al. (2012) [19] used an isolation model and patterns of incomplete lineage sorting between the two *Pan* species and humans, and estimated the split time to be about 990 kya. Using the same scaling factor (mutation rate 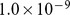 per year) we find a split time estimate of around 800 kya. Our estimate is consistent with previous estimates based on IM models from Won and Hey (2005) [27] and Hey (2010) [28], which also inferred no migration between bonobos and common chimpanzees. It is also close to the estimate of Becquet and Przeworski (2007) [8]. This study detected a weak signal of gene flow between eastern chimpanzees and bonobos, but the whole-genome analysis of Prüfer *et al.* (2012) [19] does not indicate gene flow between bonobos and any of the common chimpanzee sub-species. (see Text S2, Figure 7).

Thus, most estimates for the bonobo-chimpanzee separation are largely consistent with our results and there is little evidence against allopatry. If we use recent estimates of present-day human mutation rate of about 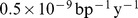 instead of 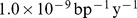, the above time estimates should be multiplied by 2 (see Figure 12), putting the bonobo-chimpanzee separation around 1.5 Mya, closer to but still likely postdating the formation of the Congo River.

### Speciation of humans and chimpanzees

The human-chimpanzee speciation has been the focus of considerable attention in previous studies, most of which have assumed a simple (allopatric) speciation model. However, as shown in Figure 11, we find evidence favouring a non-allopatric model, in which the initial divergence was followed by gene flow for an extended period.

Considering the evidence reported here and previously for gene flow between species within the great ape genera [20], [21] and between modern humans and archaic humans [31]–[35], it appears that non-allopatric speciation is not uncommon within the great apes, and it is plausible that a similar scenario may have applied to the split between humans and our closest relatives. Patterson *et al.* (2006) [36] proposed a complex scenario involving an initial split, followed by isolation, then an admixture event and finally an isolation between the species. Several subsequent analyses concluded that an allopatric speciation could not be rejected but did not conclusively rule out more complex scenarios [5], [7], [20], [37]–[40]. Our method does not explicitly model admixture so we cannot directly test the hypothesis presented by Patterson *et al.* In particular the model does not distinguish between gene flow occurring as a period of limited ongoing exchange between diverging populations or in the form of one or more admixture/hybridisation events. This is a key question to explore in future extensions.

### Future perspectives

The exploitation of whole genome data in a demographic inference model is made computationally efficient by the Markov assumption underlying CoalHMMs, and should increase the statistical power for parameter estimation and for comparing different demographic scenarios. However the construction of complex demographic models with CoalHMMs is complicated by the mathematics involved in specifying transition probabilities between local genealogies.

The model we have presented in this paper is an initial attempt at building a speciation model using a CoalHMM, but the underlying framework, using a continuous time Markov chain to model transitions between genealogies, generalises straightforwardly to other demographic scenarios (see [41] for initial work in this direction). By varying the coalescence rate in different time intervals, the model captures variation in the effective population size in the past in essentially the same way as the pairwise sequential Markov coalescence (PSMC) model of Li and Durbin [1]. Varying the migration rate in a similar manner, rather than assuming a constant rate of migration during an extended speciation event, could provide information about the timing of admixture events and could also model scenarios such as a gradual speciation or the complex speciation between humans and chimpanzees suggested by Patterson et al. (2006) [36]. Adding further populations and genomes is also feasible but is limited by the state space of the continuous time Markov chain.

Using a hidden Markov model also enables us, via posterior decoding, to investigate variation in coalescent times, recombination and gene exchange along the genome. See Text S1, Section 10 and Text S2, Section 1, for initial results using posterior decoding to estimate the time to the most recent common ancestor and to detect signals of selection. Such variation is expected to be an important aspect of speciation [42], and is seen in studies of hybridisation between closely related species [43], [44]. The ability to explore it in genome-wide comparisons between populations at various stages of divergence will be important in understanding the range of evolutionary processes involved in speciation.

## Methods

### Constructing the hidden Markov model

The crux of constructing a coalescent hidden Markov model is deriving transition and emission probability matrices from the coalescence process parameters of interest. For computing the transition probabilities we take the approach from Mailund et al. (2011) [15] and construct continuous time Markov chains (CTMCs) that explicitly track the ancestry of pairs of neighboring nucleotides. From these we can compute the the transition probabilities exactly. For emission probabilities we compute the coalescence de§nsities in the models from similar CTMCs from which we compute the mean coalescence time in each time interval. Based on the mean coalescent time, we then compute the distribution of alignment columns and use these for the emission probabilities.

#### Constructing the CTMCs

The coalescence process with recombination can be formulated as a CTMC running back in time from a present day sample of genomes back until all nucleotides have found their most recent common ancestor (MRCA) [45]. In an isolation with migration model, the states corresponds to a number of lineages carrying ancestral material, distributed across the two populations, and the events that can occur are *i)* coalescence, *ii)* recombination, and *iii)* migration, each with different rates.

When constructing our coalescent hidden Markov model, we consider the case for genome segments only two nucleotides long. In this case, we get a system of a relatively small number of states where we can explicitly construct the rate matrix and compute transition probabilities exactly. The process is very similar to the isolation model in Mailund et al. (2011) [15] and was also used by Simonsen and Churchill (1997) [46] and Slatkin and Pollack (2006) [47]. Although manageable, constructing the state space and rate matrix manually is still tedious and error prone, so we instead construct a generative model from which we can enumerate the complete state space of all states and transitions, and from this easily construct the rate matrix.

In this model, we represent lineages at a single nucleotide as sets. The sets 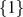 and 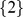 denote sequences 1 and 2 before they have found their MRCA, while 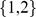 denote their MRCA. We then model two neighboring nucleotides as pairs of such states, so e.g. 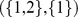 denote a lineage where the left nucleotide has found its MRCA and is linked on the right to a nucleotide from the first sequence that has not found its MRCA with sequence 2, and 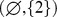 denotes a lineage where sequence two on the right nucleotide has not found its MRCA with sequence 1 and is not linked with ancestral material on the left.

To assign lineages to species, we pair them again, and let 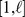 denote that lineage 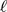 is in species 1 and let 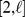 denote that lineage 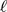 is in species 2. A state in the CTMC corresponds to a set of such lineages assigned to species.

**Figure 13:**
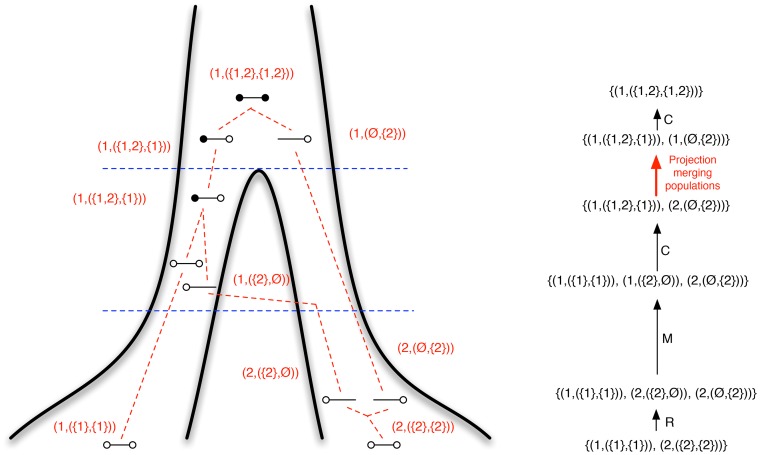
Ancestral recombination graph and state space. On the left is shown an ancestral recombination graph for two genomes with two nucleotides. Lineages, in the notation we use for constructing the CTMCs, are shown in red. On the right is shown the corresponding list of transitions int he CTMC with the type of transitions on the arrows: recombination (R), migration (M) and coalescence (C). The transition from the two separate populations to the ancestral population is a special transition – the projection matrix in the CTMC – shown in red.

We define the following transitions of states:

1. Coalescence: 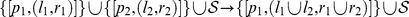 if 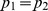

2. Recombination: 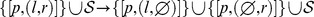

3. Migration: 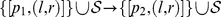 if 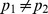.

where 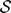 denotes the set of other lineages in the state. Figure 13 illustrates this state space notation with an example ancestral recombination graph.

As the initial state of the system, we use the state where sequence 1 is in species 1, sequence 2 is in species 2, and both sequences have their left and right nucleotides linked, 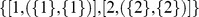, and we then compute a graph of all states reachable from this state through the transitions above, labeling each edge with the kind of transformation (coalescence, recombination or migration). From this state space we construct a rate matrix by first assigning a number to each state, and then setting rates in the matrix in entries corresponding to edges in the graph.

When constructing the rate matrix from the state space, we set migration rates to zero for the time period where we do not allow gene flow, from 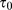 to 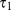, and for the ancestral population we only consider the first population and do not allow migrations to the second. When moving to that CTMC we then first project all lineages into the first population. To reflect that the effective population size can be different in the different populations, we allow different coalescence rates in the different populations, and a different coalescence rate in the ancestral population than in the two present day populations. In the following, we let 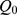 denote the rate matrix for the time interval 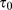 to 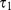, 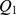 denote the rate matrix for the time interval 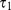 to 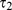, and 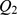 denote the rate matrix for the time interval above 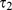.

#### Computing the transition probability matrix

Let 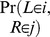 denote the probability that the left nucleotides finds their MRCA in time interval 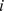 and that the right nucleotides find their MRCA in time interval 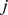. Our goal now is to compute these probabilities, and from these obtain the transition probabilities for the hidden Markov model. We do this by summing over all paths of states in the CTMC where the left nucleotide coalesces in interval 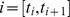 and where the right nucleotide coalesces in interval 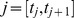.

First, we compute CTMC transition probability matrices for individual time intervals. Let 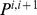 denote such a matrix, i.e. let 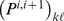 denote the probability of moving from state 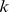 at the beginning of interval 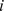 to state 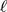 at the end of interval 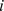. When the same CTMC is used in interval 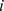 and 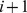 this is obtained from CTMC theory as 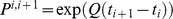 where 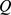 is the rate matrix for the CTMC. When we use a different CTMC in interval 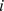 and 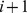, i.e. when 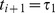 or 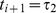, the probability matrix is complicated by having different sets of states in interval 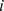 and 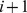. To deal with this, we define an injection matrix, 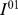, that injects the smaller state space of the first CTMC, where no gene flow is possible, into the larger state space where it is, and a projection matrix, 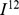, that projects the separate populations in the second CTMC into the third CTMC with panmictic mating.

Let
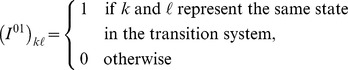
and
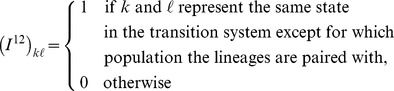
Using these injection and projection matrices, the transition probability matrices for individual intervals are computed as
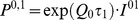
for the interval between the present and the time where gene flow is allowed,
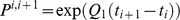
for 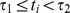, the intervals where the ancestral species/populations are separated but where gene flow is allowed,
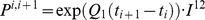
for 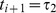, the final interval with gene flow, and
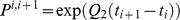
for 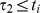, the time intervals with panmictic mating (using 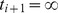 for the final interval).

Second, we compute the CTMC transition probability matrices across several intervals. These can be computed by simple matrix multiplication, since the probability of being in state 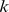 at time 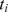 and being in state 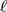 at time 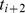 is
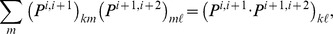
so we define 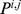 for 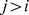 as
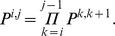
Now, let 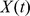 denote the two-nucleotide state of the CTMC at time 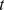 and let 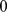 denote the initial state, where we assume that the initial state is always 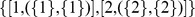 and that this has index 0 and 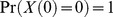. In all analyses, we have assumed that the initial state is the state where the nucleotides have not found their MRCA, and where the left and right nucleotides in the two sequences are linked. Let 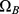 denote all CTMC states where neither left nor right nucleotides have found their MRCA, let 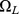 denote the states where the left but not the right nucleotides have found their MRCA, let 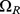 denote the states where the right but not the left nucleotides have found their MRCA, and finally let 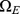 denote the states where both left and right nucleotides have found their MRCA.

If 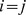 we obtain the joint probability 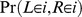 from
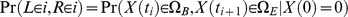


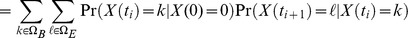


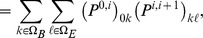
if 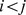 we get
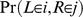


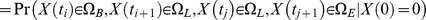


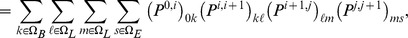
and symmetrically, if 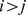 we get
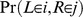


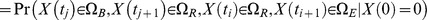


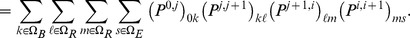



To reduce the computation time for calculating the sums, we use a dynamic programming algorithm.

Given the joint probabilities of having the left nucleotide coalesce in time interval 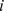 and the right in interval 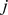, the transition probability matrix for the hidden Markov model is computed simply as the conditional probability 
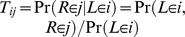
 where 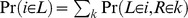.

#### Computing the emission probability matrix

For computing the emission probabilities, we use the same approach as in our previous work [13]–[15] and compute the probability of each alignment column conditional on a coalescence time at the mean of a time interval. Conditional on the divergence of the two sequences, the alignment column probabilities can be computed using common phylogenetic methods, and in this model we use the simple Jukes-Cantor model.

For computing the mean coalescence time in each time interval, we can use the truncated exponential density as in our previous models for the intervals further back in time than 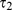, but for the time intervals between 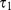 and 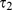 then coalescence time density is complicated by having to deal with both migration and coalescence events [48]. Below, we show how we can compute the mean coalescence time in each interval between 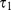 and 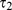.

To deal with this, we set up a CTMC similar to those for the transition probabilities, but considering only a single nucleotide per genome. In the following, 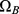 will denote states for this CTMC where the nucleotides from the two genomes have not coalesced, and 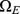 will denote states where the nucleotides have found their MRCA.

From a CTMC we can compute the mean time until an absorbing state (i.e. coalescence) using standard CTMC theory (see e.g. Tavaré (1979) [49]), but in our application we need to condition on coalescing within the given time interval, i.e. we want to compute the end-conditioned expectation
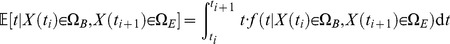
where 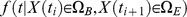 is the conditional coalescence density
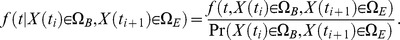
We assume implicitly, as for the transition probabilities, that the state at time 0 is known, i.e. 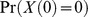 with probability 1, so
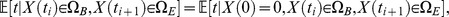
thus, we need to compute
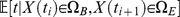


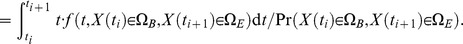



The probability 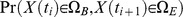 is just the probability of coalescing in interval 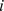, which we computed as 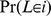 as part of the transition probability matrix.

Let 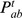 denote the probability of going from state 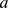 to state 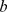 in time 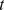, computed as 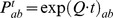, where 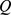 is the rate matrix for the CTMC. For the coalescence density we then have
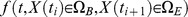


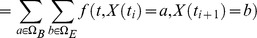


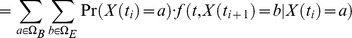
and so
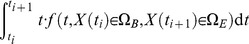


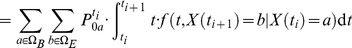



For the integral, we now use a change of variable, 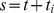, set 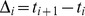 and get
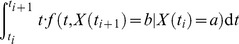


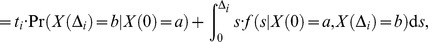
using that the CTMC is time homogeneous and that the conditional density 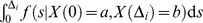 integrates to one, so
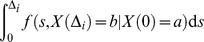


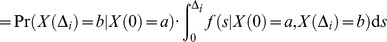


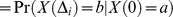
and we get
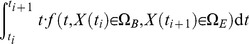


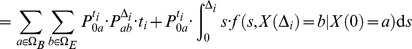


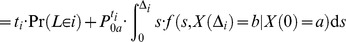
where the last equality follows from
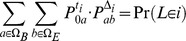
and is the probability of coalescing in interval 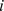.

The density, 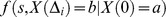 is computed by summing over all state transitions from 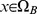 to 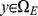 at time 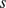


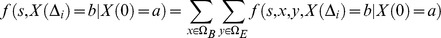
where the density 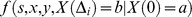 is given by
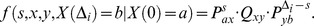
We then use an eigen-value decomposition of the rate matrix 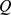. Let 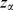 and 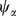 denote the eigen-vectors and eigen-values, respectively, of 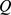, and let 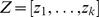 denote the matrix of eigen-vectors. We then have
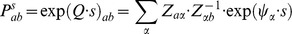
and so
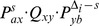


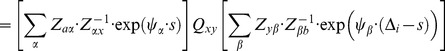


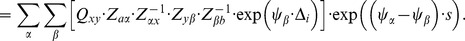



Now, let
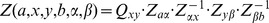
and note that 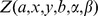 does not depend on either 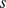, 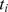 or 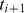. Let 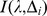 denote the integral
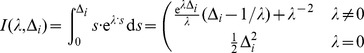
We then have
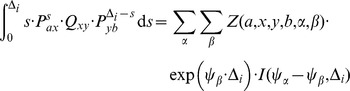
which is a function of 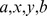 and 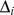, and we denote it 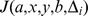 and get
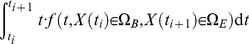


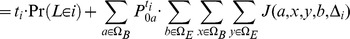


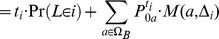
where
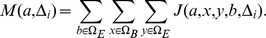
Putting it all together, we get
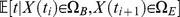


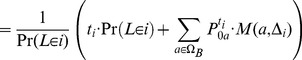


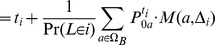



For computational efficiency, we first compute all 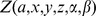, which are independent of the time interval. This can be done efficiently using dynamic programming. We then use equally spaced time break points, so 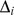 is the same for all intervals, and then only need to compute 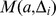 for all 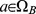 once, and not for each time interval.

### Parameter estimation

The model was implemented in Python, and we used the numerical optimization functionality from the scipy optimize module, function fmin to find the maximum likelihood parameters and HMMLib [50] to compute the likelihood for the hidden Markov model. The implementation is available as Dataset S2.

### Simulation setup

For our simulation experiments, we simulated ancestral recombination graphs from the coalescent with recombination process using the CoaSim tool [51]. From this we extracted local (tree) genealogies and simulated sequences over these using the Bio++ suite [52] with the Jukes-Cantor JC69 substitution model.

### Genome alignments

Genome sequence alignments between eastern and western gorillas and between Bornean and Sumatran orang-utans were generated as follows. Illumina paired-end reads for Mukisi, an eastern lowland gorilla (*Gorilla beringei graueri*) [20] were aligned using BWA [53] to the gorilla reference assembly (UCSC identifier gorGor3.1), which represents the genome sequence of a western lowland individual (*Gorilla gorilla gorilla*). Similarly, Illumina reads from kb5404, a Bornean orang-utan (*Pongo pygmaeus*) [21] were mapped using Stampy version 1.0.13 [54] to the (orang-utan reference assembly (ponAbe2), which represents the Sumatran species (*Pongo abelii*).

In both cases mapped reads were merged using Picard (http://picard.sourceforge.net), and duplicate reads were removed and pileup information generated using Samtools [55]. Consensus sequences were called at every position on each reference based on the majority vote of aligned bases, with positions having no aligned reads represented by ‘N’. This produced two consensus genome sequences, each the same length as the corresponding reference, one representing eastern lowland gorilla and the other Bornean orang-utan. These sequences were used in subsequent analyses.

Genome alignments for the analysis of the chimpanzee-bonobo, human-chimpanzee and human-bonobo splits were produced as described in Prüfer *et al.* [19, Supplementary Information 3]. Briefly, pairwise lastz [56] alignments were generated from bonobo (scaffolds, i7) to human, chimpanzee (panTro2) to human and orang-utan (ponAbe2) to human. These alignments were processed using the programs of the UCSC genome browser pipeline [57], [58] and joined on the human reference using the multiz package [59]. Bonobo and chimpanzee bases with a base quality lower than 30 were masked in the resulting multiple genome alignment.

## Supporting Information

Dataset S1 Analysis results for all ape analyses.
(XLSX)

Dataset S2 Source code for the CoalHMM.
(GZ) Click here for additional data file.

Text S1 Report describing simulation experiments and results.
(PDF)

Text S2 Report describing data analysis results not included in the main manuscript.
(PDF)

## References

[1] Li H, Durbin R (2011) Inference of human population history from individual whole-genome sequences. Nature 475: 493–496. 21753753 10.1038/nature10231 PMC3154645

[2] Burgess R, Yang Z (2008) Estimation of hominoid ancestral population sizes under bayesian coalescent models incorporating mutation rate variation and sequencing errors. Mol Biol Evol 25: 1979–1994. 18603620 10.1093/molbev/msn148

[3] Wang Y, Hey J (2010) Estimating divergence parameters with small samples from a large number of loci. Genetics 184: 363–379. 19917765 10.1534/genetics.109.110528 PMC2828718

[4] Hey J (2010) Isolation with migration models for more than two populations. Mol Biol Evol 27: 905–920. 19955477 10.1093/molbev/msp296 PMC2877539

[5] Yang Z (2010) A likelihood ratio test of speciation with gene ow using genomic sequence data. Genome Biology and Evolution 2: 200–211. 20624726 10.1093/gbe/evq011 PMC2997537

[6] Gronau I, Hubisz MJ, Gulko B, Danko CG, Siepel A (2011) Bayesian inference of ancient human demography from individual genome sequences. Nature Genetics 43: 1031–1034. 21926973 10.1038/ng.937 PMC3245873

[7] Zhu T, Yang Z (2012) Maximum Likelihood Implementation of an Isolation-with-Migration Model with Three Species for Testing Speciation with Gene Flow. Mol Biol Evol 10.1093/molbev/mss118 22504520

[8] Becquet C, Przeworski M (2007) A new approach to estimate parameters of speciation models with application to apes. Genome Res 17: 1505–1519. 17712021 10.1101/gr.6409707 PMC1987350

[9] Wiuf C, Hein J (1999) Recombination as a point process along sequences. Theor Popul Biol 55: 248–59. 10366550 10.1006/tpbi.1998.1403

[10] McVean GAT, Cardin NJ (2005) Approximating the coalescent with recombination. Philosophical transactions of the Royal Society of London Series B, Biological sciences 360: 1387–1393. 16048782 10.1098/rstb.2005.1673 PMC1569517

[11] Marjoram P, Wall JD (2006) Fast “coalescent” simulation. BMC genetics 7: 16. 16539698 10.1186/1471-2156-7-16 PMC1458357

[12] Chen GK, Marjoram P, Wall JD (2009) Fast and exible simulation of DNA sequence data. Genome Research 19: 136–142. 19029539 10.1101/gr.083634.108 PMC2612967

[13] Hobolth A, Christensen OF, Mailund T, Schierup MH (2007) Genomic relationships and speciation times of human, chimpanzee, and gorilla inferred from a coalescent hidden markov model. PLoS Genet 3: e7. 17319744 10.1371/journal.pgen.0030007 PMC1802818

[14] Dutheil JY, Ganapathy G, Hobolth A, Mailund T, Uyenoyama MK, et al (2009) Ancestral population genomics: The coalescent hidden markov model approach. Genetics 183: 259–274. 19581452 10.1534/genetics.109.103010 PMC2746150

[15] Mailund T, Dutheil JY, Hobolth A, Lunter G, Schierup MH (2011) Estimating divergence time and ancestral effective population size of bornean and sumatran orangutan subspecies using a coalescent hidden markov model. PLoS Genet 7: e1001319. 21408205 10.1371/journal.pgen.1001319 PMC3048369

[16] Paul JS, Steinrucken M, Song YS (2011) An accurate sequentially Markov conditional sampling distribution for the coalescent with recombination. Genetics 187: 1115–1128. 21270390 10.1534/genetics.110.125534 PMC3070520

[17] Hobolth A, Dutheil JY, Hawks J, Schierup MH, Mailund T (2011) Incomplete lineage sorting patterns among human, chimpanzee, and orangutan suggest recent orangutan speciation and widespread selection. Genome Research 21: 349–356. 21270173 10.1101/gr.114751.110 PMC3044849

[18] Kong A, Thorleifsson G, Gudbjartsson DF, Masson G, Sigurdsson A, et al (2010) Fine-scale recombination rate differences between sexes, populations and individuals. Nature 467: 1099–1103. 20981099 10.1038/nature09525

[19] Prüfer K, Munch K, Hellmann I, Akagi K, Miller JR, et al (2012) The bonobo genome compared with the chimpanzee and human genomes. Nature 486: 527–531. 22722832 10.1038/nature11128 PMC3498939

[20] Scally A, Dutheil JY, Hillier LW, Jordan GE, Goodhead I, et al (2012) Insights into hominid evolution from the gorilla genome sequence. Nature 483: 169–175. 22398555 10.1038/nature10842 PMC3303130

[21] Locke DP, Hillier LW, Warren WC, Worley KC, Nazareth LV, et al (2011) Comparative and demographic analysis of orang-utan genomes. Nature 469: 529–533. 21270892 10.1038/nature09687 PMC3060778

[22] Scally A, Durbin R (2012) Revising the human mutation rate: implications for understanding human evolution. Nat Rev Genet 13: 745–753. 22965354 10.1038/nrg3295

[23] Langergraber KEK, Prüfer KK, Rowney CC, Boesch CC, Crockford CC, et al (2012) Generation times in wild chimpanzees and gorillas suggest earlier divergence times in great ape and human evolution. PNAS 109: 15716–15721. 22891323 10.1073/pnas.1211740109 PMC3465451

[24] Kong A, Frigge ML, Masson G, Besenbacher S, Sulem P, et al (2012) Rate of de novo mutations and the importance of father's age to disease risk. Nature 488: 471–475. 22914163 10.1038/nature11396 PMC3548427

[25] Thalmann O, Fischer A, Lankester F, Pääbo S, Vigilant L (2007) The complex evolutionary history of gorillas: insights from genomic data. Mol Biol Evol 24: 146–58. 17065595 10.1093/molbev/msl160

[26] Steiper ME (2006) Population history, biogeography, and taxonomy of orangutans (genus: Pongo) based on a population genetic meta-analysis of multiple loci. J Hum Evol 50: 509–22. 16472840 10.1016/j.jhevol.2005.12.005

[27] Won YJ, Hey J (2005) Divergence population genetics of chimpanzees. Mol Biol Evol 22: 297–307. 15483319 10.1093/molbev/msi017

[28] Hey J (2010) The divergence of chimpanzee species and subspecies as revealed in multipopulation isolation-with-migration analyses. Mol Biol Evol 27: 921–933. 19955478 10.1093/molbev/msp298 PMC2877540

[29] Myers Thompson JA (2003) A model of the biogeographical journey from Proto-pan to Pan paniscus. Primates; journal of primatology 44: 191–197. 12687485 10.1007/s10329-002-0029-1

[30] Stankiewicz J, de Wit MJ (2006) A proposed drainage evolution model for Central Africa–Did the Congo ow east? Journal of African Earth Sciences 44: 75–84.

[31] Green RE, Krause J, Briggs AW, Maricic T, Stenzel U, et al (2010) A draft sequence of the Neandertal genome. Science (New York, NY) 328: 710–722. 10.1126/science.1188021 PMC5100745 20448178

[32] Reich D, Green RE, Kircher M, Krause J, Patterson N, et al (2010) Genetic history of an archaic hominin group from Denisova Cave in Siberia. Nature 468: 1053–1060. 21179161 10.1038/nature09710 PMC4306417

[33] Hammer MF, Woerner AE, Mendez FL, Watkins JC, Wall JD (2011) Genetic evidence for archaic admixture in Africa. Proceedings Of The National Academy Of Sciences Of The United States Of America 108: 15123–15128. 21896735 10.1073/pnas.1109300108 PMC3174671

[34] Reich D, Patterson N, Kircher M, Delfin F, Nandineni MR, et al (2011) Denisova admixture and the first modern human dispersals into southeast Asia and oceania. American journal of human genetics 89: 516–528. 21944045 10.1016/j.ajhg.2011.09.005 PMC3188841

[35] Sankararaman SS, Patterson NN, Li HH, Pääbo SS, Reich DD (2012) The Date of Interbreeding between Neandertals and Modern Humans. PLoS Genet 8: e1002947. 23055938 10.1371/journal.pgen.1002947 PMC3464203

[36] Patterson N, Richter DJ, Gnerre S, Lander ES, Reich D (2006) Genetic evidence for complex speciation of humans and chimpanzees. Nature 441: 1103–1108. 16710306 10.1038/nature04789

[37] Wakeley J (2008) Complex speciation of humans and chimpanzees. Nature 452: E3–4; discussion E4. 18337768 10.1038/nature06805

[38] Presgraves DC, Yi SV (2009) Doubts about complex speciation between humans and chimpanzees. Trends in ecology and evolution 24: 533–540. 19664844 10.1016/j.tree.2009.04.007 PMC2743777

[39] Webster MT (2009) Patterns of autosomal divergence between the human and chimpanzee genomes support an allopatric model of speciation. Gene 443: 70–75. 19463924 10.1016/j.gene.2009.05.006

[40] Yamamichi MM, Gojobori JJ, Innan HH (2011) An autosomal analysis gives no genetic evidence for complex speciation of humans and chimpanzees. Mol Biol Evol 29: 145–156. 21903679 10.1093/molbev/msr172 PMC3299331

[41] Mailund T, Halager A, Westergaard M (2012) Using Colored Petri Nets to Construct Coalescent Hidden Markov Models: Automatic Translation from Demographic Specifications to Efficient Inference Methods. In: Haddad S, Pomello L, editors, Application and Theory of Petri Nets. Springer Berlin/Heidelberg, pp. 32–50.

[42] Nachman MW, Payseur BA (2012) Recombination rate variation and speciation: theoretical predictions and empirical results from rabbits and mice. Philosophical transactions of the Royal Society of London Series B, Biological sciences 367: 409–421. 22201170 10.1098/rstb.2011.0249 PMC3233716

[43] Nadeau NJ, Whibley A, Jones RT, Davey JW, Dasmahapatra KK, et al (2012) Genomic islands of divergence in hybridizing Heliconius butteries identified by large-scale targeted sequencing. Philosophical transactions of the Royal Society of London Series B, Biological sciences 367: 343–353. 22201164 10.1098/rstb.2011.0198 PMC3233711

[44] Janoušek V, Wang L, Luzynski K, Dufková P, Vyskočilová MM, et al (2012) Genome-wide architecture of reproductive isolation in a naturally occurring hybrid zone between Mus musculus musculus and M. m. domesticus. Molecular Ecology 21: 3032–3047. 22582810 10.1111/j.1365-294X.2012.05583.x PMC3872452

[45] Hein J, Schierup MH, Wiuf C (2005) Gene genealogies, variation and evolution: A primer in coalescent theory. Oxford university press.

[46] Simonsen K, Churchill G (1997) A Markov Chain Model of Coalescence with Recombination. Theor Popul Biol 52: 43–59. 9356323 10.1006/tpbi.1997.1307

[47] Slatkin M, Pollack JL (2006) The concordance of gene trees and species trees at two linked loci. Genetics 172: 1979–1984. 16361238 10.1534/genetics.105.049593 PMC1456312

[48] Hobolth A, Andersen LN, Mailund T (2011) On computing the coalescence time density in an isolation-with-migration model with few samples. Genetics 187: 1241–1243. 21321131 10.1534/genetics.110.124164 PMC3070532

[49] Tavare S (1979) Note on finite homogeneous continuous-time Markov-chains. Biometrics 35: 831–834.

[50] Sand A, Pedersen C, Mailund T, Brask A (2010) HMMlib: A C++ library for general hidden Markov models exploiting modern CPUs. In: Proceedings of the 2nd International Workshop on High Performance Computational Systems Biology. IEEE, pp. 126–134.

[51] Mailund T, Schierup MH, Pedersen CNS, Mechlenborg PJM, Madsen JN, et al (2005) CoaSim: a exible environment for simulating genetic data under coalescent models. BMC Bioinformatics 6: 252. 16225674 10.1186/1471-2105-6-252 PMC1274299

[52] Dutheil J, Boussau B (2008) Non-homogeneous models of sequence evolution in the Bio++ suite of libraries and programs. BMC Evol Biol 8: 255. 18808672 10.1186/1471-2148-8-255 PMC2559849

[53] Li H, Durbin R (2009) Fast and accurate short read alignment with Burrows-Wheeler transform. Bioinformatics 25: 1754–1760. 19451168 10.1093/bioinformatics/btp324 PMC2705234

[54] Lunter G, Goodson M (2011) Stampy: a statistical algorithm for sensitive and fast mapping of Illumina sequence reads. Genome Research 21: 936–939. 20980556 10.1101/gr.111120.110 PMC3106326

[55] Li H, Handsaker B, Wysoker A, Fennell T, Ruan J, et al (2009) The Sequence Alignment/Map format and SAMtools. Bioinformatics 25: 2078–2079. 19505943 10.1093/bioinformatics/btp352 PMC2723002

[56] Harris RS (2007) Improved Pairwise Alignment of Genomic DNA. Dissertation, Pennsylvania State University.

[57] Kent WJ, Baertsch R, Hinrichs A, Miller W, Haussler D (2003) Evolution's cauldron: duplication, deletion, and rearrangement in the mouse and human genomes. Proceedings of the National Academy of Sciences of the United States of America 100: 11484–9. 14500911 10.1073/pnas.1932072100 PMC208784

[58] Dreszer TR, Karolchik D, Zweig AS, Hinrichs AS, Raney BJ, et al (2012) The UCSC genome browser database: extensions and updates 2011. Nucleic acids research 40: D918–923. 22086951 10.1093/nar/gkr1055 PMC3245018

[59] Blanchette M, Kent WJ, Riemer C, Elnitski L, Smit AFA, et al (2004) Aligning multiple genomic sequences with the threaded blockset aligner. Genome Research 14: 708–715. 15060014 10.1101/gr.1933104 PMC383317

