# Supplementary material for: A New Isolation with Migration Model along Complete Genomes Infers Very Different Divergence Processes among Closely Related Great Ape Species
Source: PLoS Genet. 2012 Dec 20;8(12):e1003125. doi: 10.1371/journal.pgen.1003125 (PMC3527290; doi:10.1371/journal.pgen.1003125)
Supplement: Text S1 — Report describing simulation experiments and results. (PDF) [file pgen.1003125.s003.pdf]

Supplemental Text 1: Simulation Experiments

Thomas Mailund[1,*], Anders E. Halager[1], and Mikkel H. Schierup[1,2]
1 Bioinformatics Research Center, Aarhus University, Aarhus, Denmark
2 Department of Biology, Aarhus University, Aarhus, Denmark
∗ **E-mail: mailund@birc.au.dk**

Contents

1 Simulation setup **2**

2 Model likelihood **2**

3 Parameter estimation **2**
3.1 Number of HMM states 2
3.2 Estimation accuracy as a function of simulated parameters 5
3.3 Estimation accuracy as a function of data size 13

4 Different coalescence rates **25**

5 Asymmetric migration **28**

6 Robustness to model assumptions **31**
6.1 Variation in mutation rate 31
6.2 Variation in recombination rate 33

7 Known and unknown genotype phase **34**

8 Model checking **37**
8.1 Likelihood ratio test 37
8.2 Akaike's information criteria 43

9 Older speciation times **45**

10 Posterior decoding **49**

1 Simulation setup

For our simulation experiments, we simulated ancestral recombination graphs from the coalescent with recombination process using the CoaSim tool [1]. From this we extracted local (tree) genealogies and simulated sequences over these using the Bio++ suite [2] with the Jukes-Cantor JC69 substitution model.

To validate the model, we simulated sequence data from the coalescence with recombination process and used the isolation-with-migration CoalHMM to infer the parameters. Based on experiments done with the isolation CoalHMM from Mailund *et al.* [3] we didn't expect to be able to infer C_1 and C_2 so we simulated data with $C_1 = C_2 = C_a$ (but see Section 4).

To keep the number of parameters to explore down, we produced simulated data with symmetric migration rates $M_{12} = M_{21}$ (but see Section 5).

2 Model likelihood

We first simulated coalescence times and sequences from the coalescence with recombination and plotted the CoalHMM likelihoods. To keep the complexity of looking at likelihoods of the model to a minimum we only allowed one or two parameters to vary, keeping the other parameters at their simulated value. We plotted likelihood curves for single parameters (see Figure 1) and for all pairs of parameters (see Figure 2).

In general, we find that the maximum likelihood is close to the simulated values. However, we do see some linearity in some of the pairs of parameters, mainly M and τ_1 and C and τ_2, that could potentially complicate maximizing the likelihood for all parameters simultaneously. It is not immediately obvious how to re-parameterize the model to avoid this linearity, so we did not explore this further.

3 Parameter estimation

The main goal of our model is to estimate parameters of the isolation-migration model, so this was the focus of our experiments.

We first generated 10 independent 1Mbp segments and analyzed them jointly. For all simulations we used a coalescence rate of $C = 2,500$ – corresponding to an effective population size of $N_e = 10,000$ assuming a substitution rate of $\mu = 10^{-9}$ substitution per year and 20 years per generation – and a recombination rate of $R = 0.4$ – corresponding to 0.8 cM/Mb with the assumed mutation rate and generation time. We simulated 10 independent data sets for each combination of parameters $\tau_1 \in \{0.00025, 0.00050\}$ (250 and 500 thousand years ago), $\tau_2 \in \{0.001, 0.002\}$ (1 and 2 million years ago), and $M \in \{62.5, 125.0, 250.0\}$.

For maximum likelihood parameter estimation, we used the numerical optimization functionality from the scipy optimize module and HMMLib [4] to compute the likelihood for the hidden Markov model.

3.1 Number of HMM states

While not exactly a model parameter, the number of states to use in the hidden Markov model, given by the number of time intervals used, is a model choice. To test the effect of this, we estimated parameters with 5, 10, 15, and 20 time intervals in the gene-flow period (from τ_1 to τ_2) and in the ancestral population (above τ_2).

Figure 3 shows the estimation accuracy of the coalescence rate. For all configurations, the parameter seems to be well recovered. Figure 4 show the estimation accuracy of the recombination rate. For all configurations, this rate is under-estimated. This is consistent with the bias in recombination rate estimates in the isolation-model CoalHMM [3].

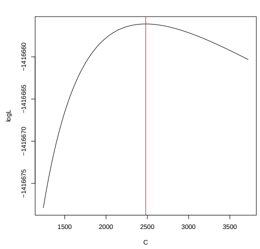

(a) Likelihood for C

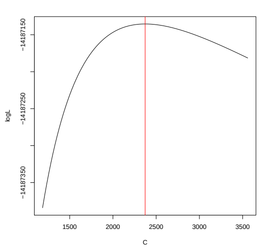

(b) Likelihood for C

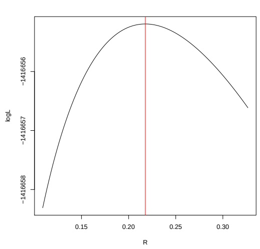

(c) Likelihood for R

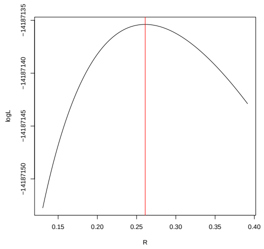

(d) Likelihood for R

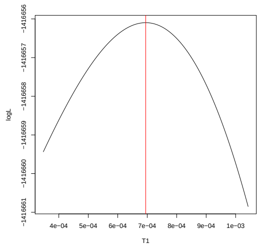

(e) Likelihood for τ_1

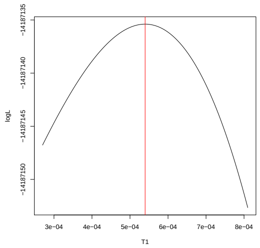

(f) Likelihood for τ_1

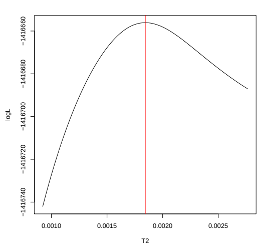

(g) Likelihood for τ_2

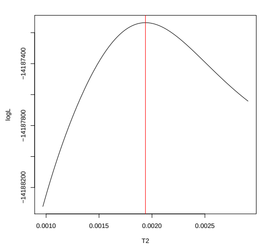

(h) Likelihood for τ_2

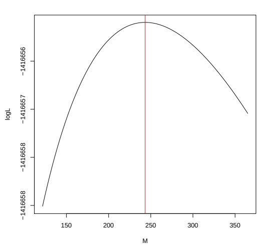

(i) Likelihood for M

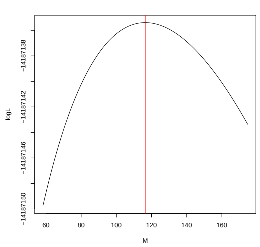

(j) Likelihood for M

Figure 1. Likelihood curves for individual parameters. Each sub-figure shows the likelihood for a single parameter, where all other parameters are kept at their simulated value. Each parameter likelihood is shown for two different simulated datasets. The true value is shown in the middle of the plot as a vertical red line.

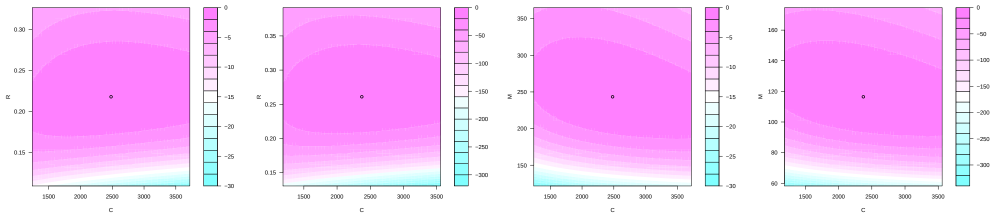

(a) Likelihood for C and R (b) Likelihood for C and R (c) Likelihood for C and M (d) Likelihood for C and M

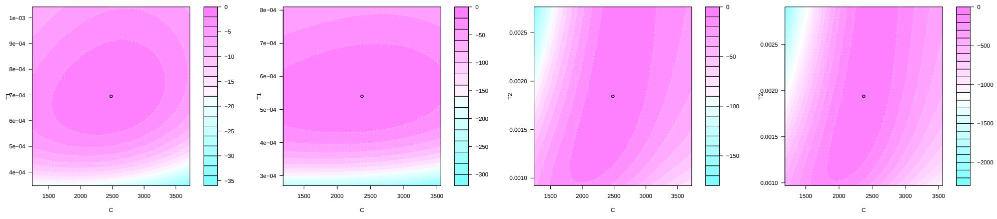

(e) Likelihood for C and τ_1 (f) Likelihood for C and τ_1 (g) Likelihood for C and τ_2 (h) Likelihood for C and τ_2

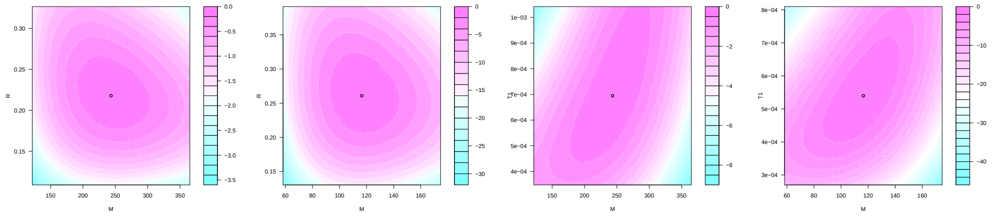

(i) Likelihood for M and R (j) Likelihood for M and R (k) Likelihood for M and τ_1 (l) Likelihood for M and τ_1

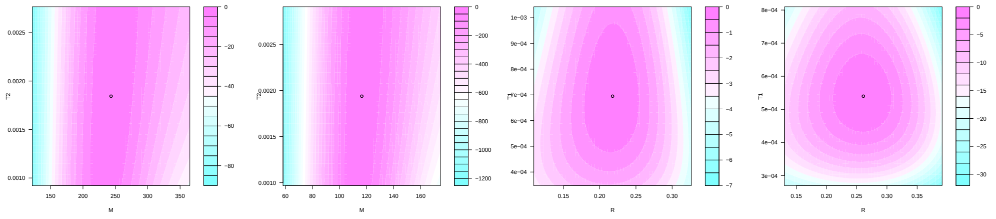

(m) Likelihood for M and τ_2 (n) Likelihood for M and τ_2 (o) Likelihood for R and τ_1 (p) Likelihood for R and τ_1

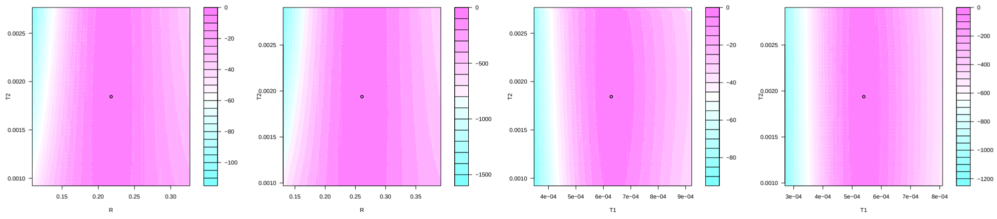

(q) Likelihood for R and τ_2 (r) Likelihood for R and τ_2 (s) Likelihood for τ_1 and τ_2 (t) Likelihood for τ_1 and τ_2

Figure 2. Likelihood curves for pairs of parameters. Each sub-figure shows the likelihood surface for pair parameters, where all other parameters are kept at their simulated value. Each likelihood is shown for two different simulated datasets. The true values are shown in the center of the plot as a circle.

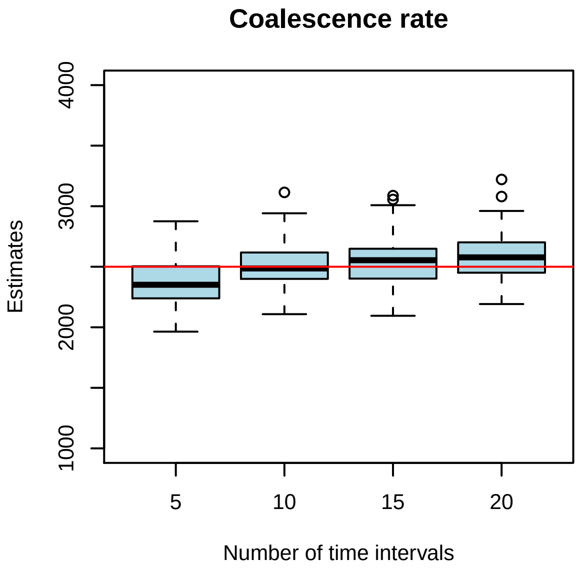

Figure 3. Coalescence rate estimates. Box-plots showing the accuracy of estimated coalescence rates as a function of the number of time intervals used in the CoalHMM. Each box-plot contains results for all combination of parameters. The horizontal red line indicates the true simulated value.

Figure 5 shows the estimation accuracy of the time where gene-flow stops completely (τ_1 on the left) and when the ancestral population split in two (τ_2 on the right). The latter is generally well recovered, while the former is recovered but with a larger uncertainty and possibly a slight up-wards bias.

Figure 6 shows the estimation accuracy of the migration rate. The median estimate seems to recover the true value, with a possible slight over-estimation bias, but the distribution of estimates has a very wide right-tail, see Figure 7.

For most parameter estimates, the number of HMM states does not seem to have a large impact on the estimation accuracy, but the variance in estimates of migration rates is large for 5 time intervals and is reduced when the number of states is increased. It is acceptable with 10 time intervals, however, and since the HMM algorithms are quadratic in the number of states, we use 10 time intervals in all analyses unless otherwise stated.

3.2 Estimation accuracy as a function of simulated parameters

We would expect the estimation accuracy of migration rates and split times to depend on the true values of these; if the interval where migration is short, and the migration rate low, we expect very few coalescence events in this interval to make inference from, and if this interval is recent, we will have very few mutations in the alignment to infer the coalescence time from. We explored this by plotting estimated parameters for each combination of τ_1, τ_2 and M separately.

Figure 8 shows estimates of τ_1 and τ_2 for all combinations of simulated values. We see that τ_2 is less sensitive to the simulated values, although with a larger uncertainty in estimated values for the shortest migration interval. For τ_1 we see a large variance for the two shortest migration intervals, and an over-estimation for the smallest migration rate in the migration interval $[\tau_1 = 0.00025, \tau_2 = 0.001]$. This is perhaps not surprising considering that we expect to have very few coalescence events very recently in

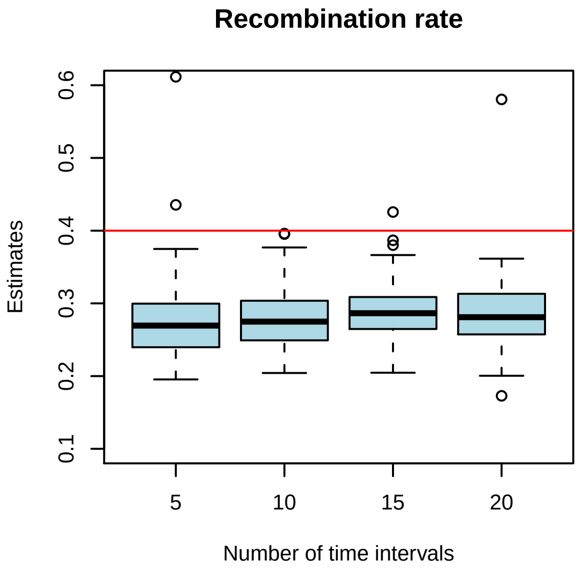

Figure 4. Recombination rate estimates. Box-plots showing the accuracy of estimated recombination rates as a function of the number of time intervals used in the CoalHMM. Each box-plot contains results for all combination of parameters. The horizontal red line indicates the true simulated value.

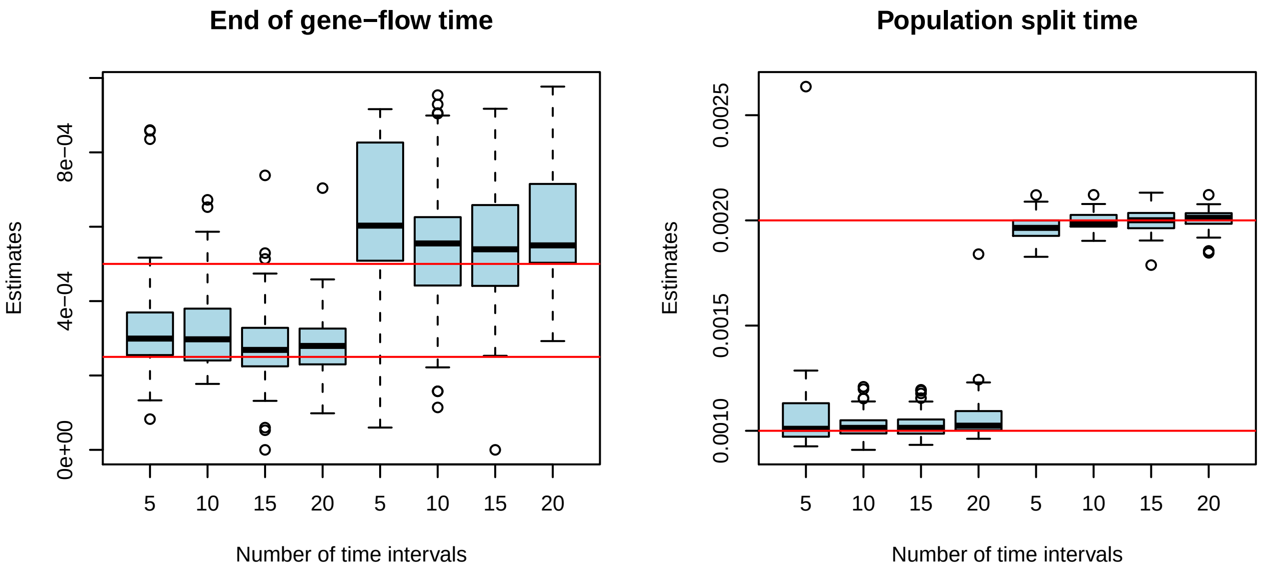

Figure 5. Split time estimates. Box-plots showing the accuracy of estimated split time as a function of the true split times and the number of time intervals used in the CoalHMM. Plots on the left shows the estimates of the time when gene-flow subsides (τ_1) while plots on the right shows the estimates of the time when the ancestral population split in two (τ_2). Each box-plot contains results for all combination of parameters other than τ_1 on the left and τ_2 on the right. The horizontal red lines indicates the true simulated values.

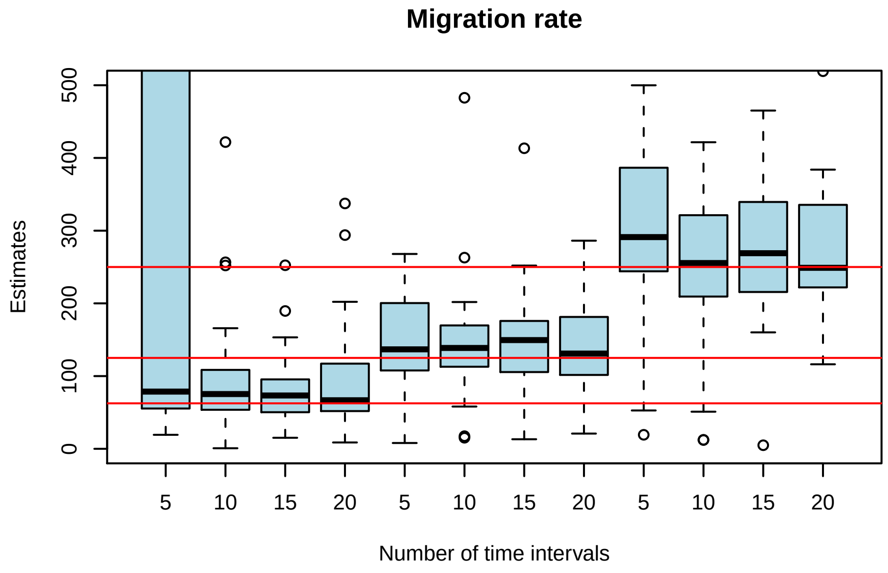

Figure 6. Migration rate estimates. Box-plots showing the accuracy of estimated migration rates as a function of the true migration rates and the number of time intervals used in the CoalHMM. Each box-plot contains results for all combination of parameters other than the migration rate. The horizontal red lines indicates the true simulated values.

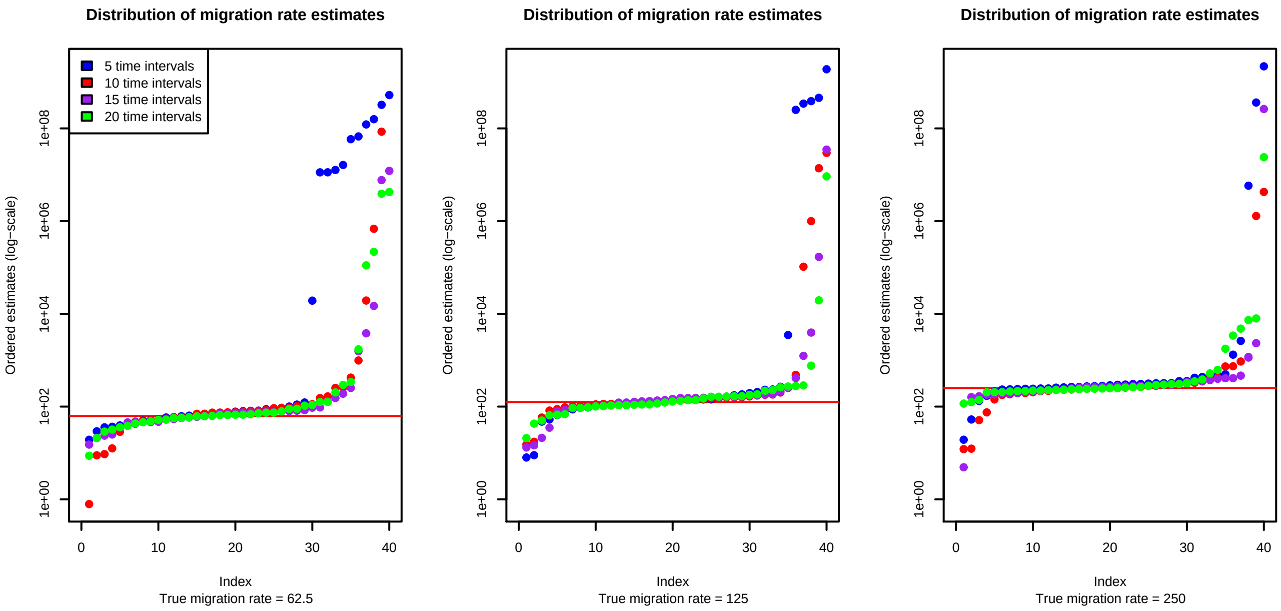

Figure 7. Distribution of migration rate estimates. Ordered estimates of migration rates for the three different simulated rates and for the different configurations of time intervals. The distribution of estimates show a very wide right-tail, a problem that seems to be worse for few time intervals and low migration rates.

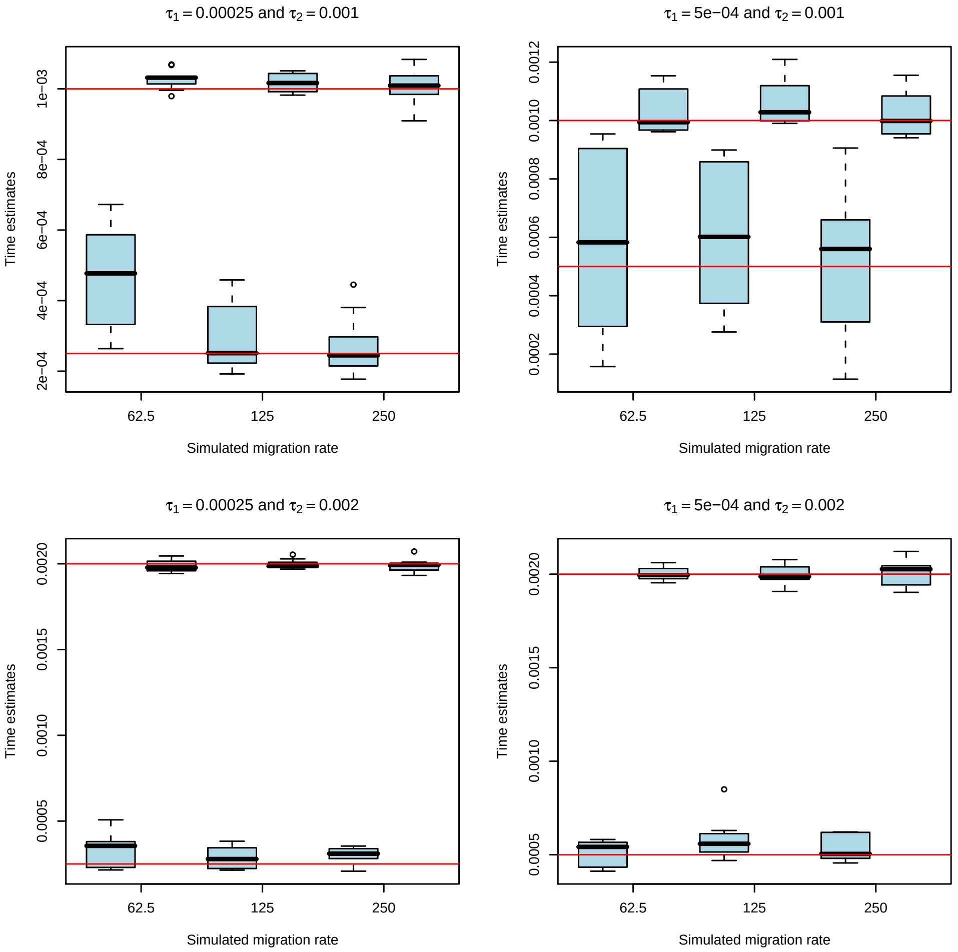

Figure 8. Estimates of split times for combinations of times and migration rates. Boxplots of estimates of split times for each combination of simulated times and migration rates.

the migration period, and those we do see are unlikely to contain mutations. Alignment segments with a coalescence event in the migration period and also containing mutations, will be the later coalescence times, and τ_1 will fit to these rather than the more recent but invisible ones.

Figure 9 shows estimates of M for all combinations of simulated values. As expected, we see larger variance in estimates for shorter migration periods, with the largest uncertainty in the smallest migration interval where few migration events are observed. For the most recent τ_1 the migration rate is overestimated. The explanation for this is the general correlation between estimates of τ_1 and M, see Figure 10. In this interval we overestimate τ_1 as discussed above, and at the same time overestimate M.

Seeing the influence of simulated parameters on M and τ_1 estimates, we also explored possible correlations between M and C and M and R estimates for all combinations of τ_1, τ_2 and M simulated parameters, see Figure 11 and Figure 12. We see no correlations between estimates or simulated values here. Nor do we see correlations between estimates of R and C, Figure 13, between R and τ_1, Figure 14, between R and τ_2, Figure 15, between C and τ_1, Figure 16, but we see a very slight positive correlation

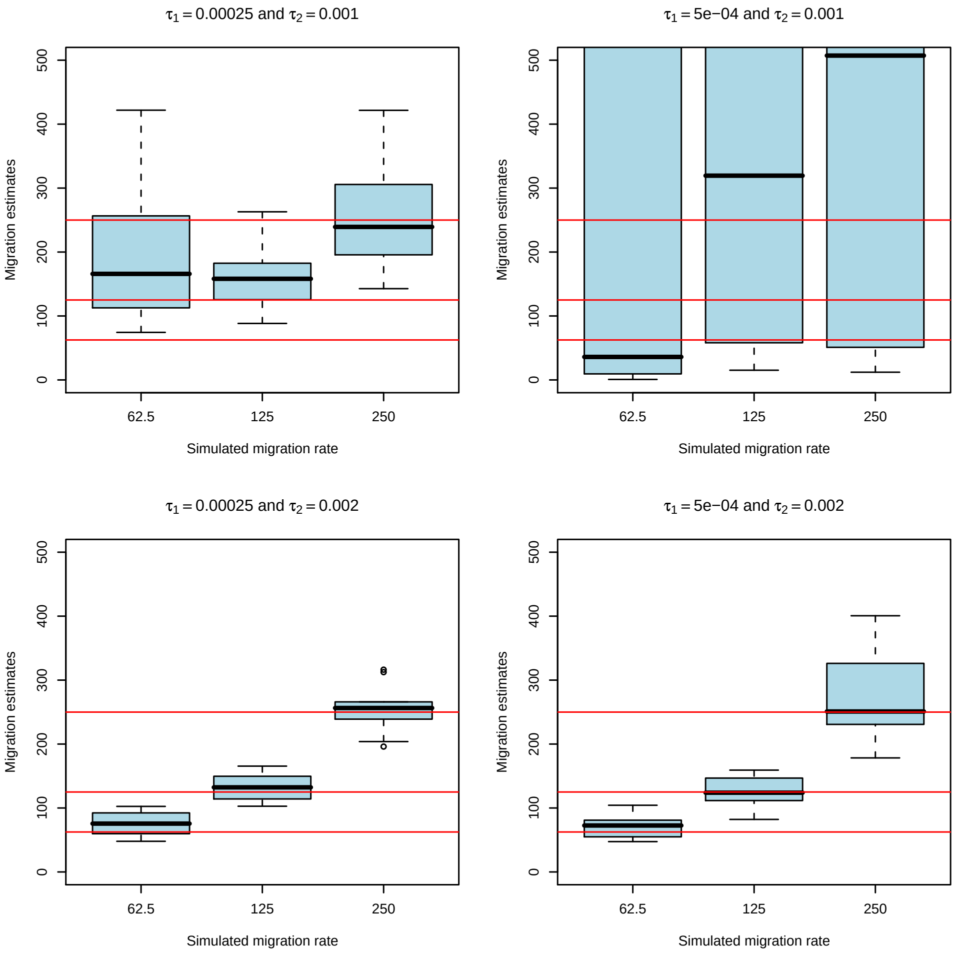

Figure 9. Estimates of migration rates for combinations of times and migration rates. Boxplots of estimates of migration rates for each combination of simulated times and migration rates.

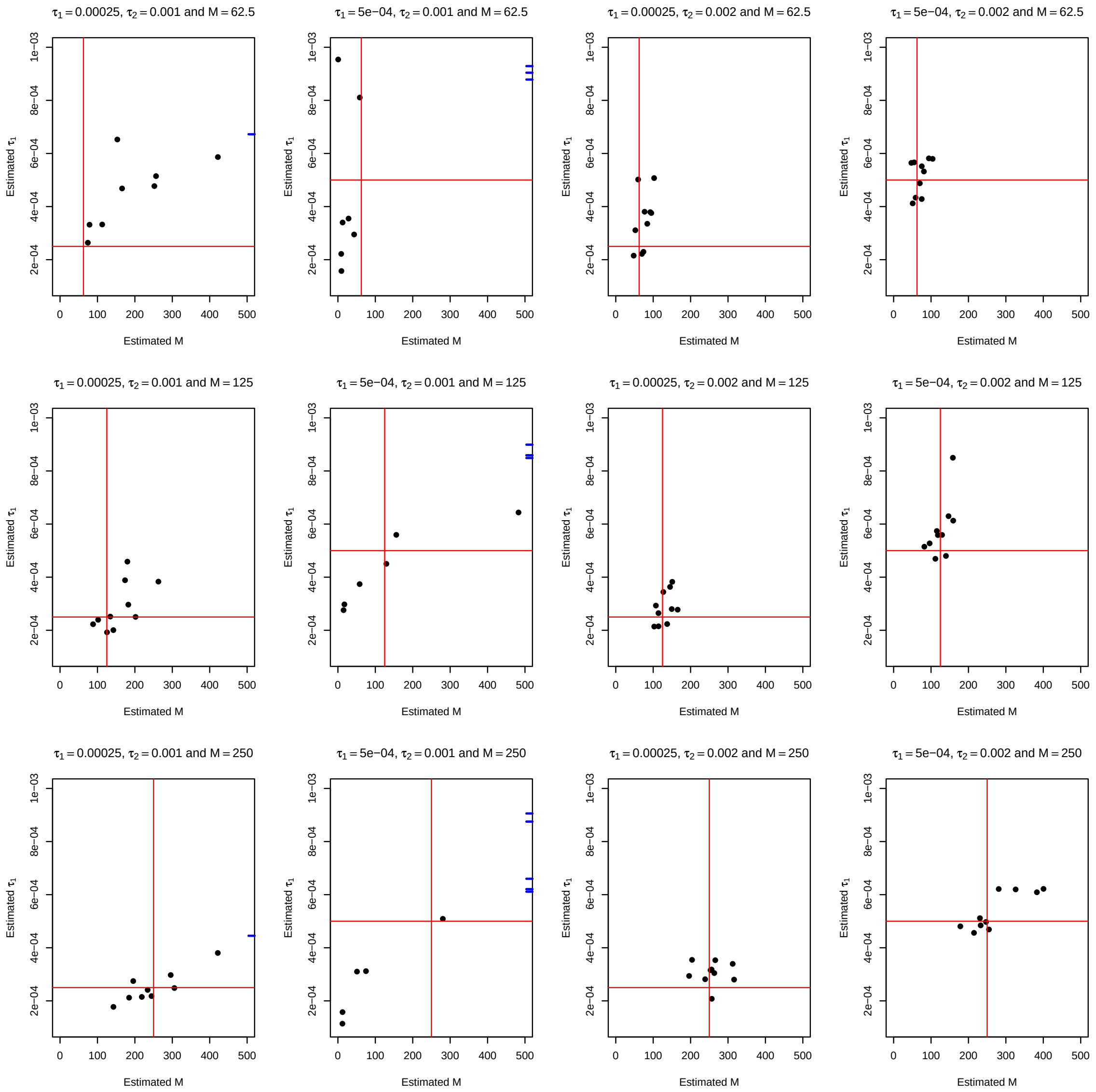

Figure 10. Joint estimates of M and τ_1. For each combination of simulated values for τ_1, τ_2, and M, the plots show the joint estimates of τ_1 and M. The axes are equal on all plots, and when an estimate falls outside of the span of M values show, it is shown as a blue rug.

between C and τ_2, Figure 17.

We then tried varying the coalescence rate, to match an effective population size equal to 10000, 20000 and 30000, see Figure 18. We see a slight effect on the split time estimates, and with the extra data points from this experiment, a positive correlation between the estimates becomes apparent, see Figure 19. We also see a slight effect in the estimation of the recombination rate, and a large effect in the estimation of the migration rate. We do not see a correlation between the recombination rate and migration rate estimation, though, see Figure 20. The underestimation of the migration rate we see for larger effective population sizes can be explained by the relative rate of coalescences and migrations. For larger effective population sizes, the coalescence rate is smaller, and we therefore see fewer migration events causing the model to underestimate this rate, exactly like it underestimates small migration rates for a fixed coalescence rate.

3.3 Estimation accuracy as a function of data size

We would expect the variation in estimates to depend on the data size. To explore this, we simulated data sets of varying alignment lengths: 1, 10 and 20 Mbp, here all with the same fixed simulation parameters. Figure 21 shows the result. The variance in estimators is clearly reduced when going from 1Mbp to 10Mbp, but less so when going from 10Mbp to 20Mbp. With the reduced variance in estimation, the bias in estimating the recombination becomes clear, as does a slight upwards bias in estimating τ_1.

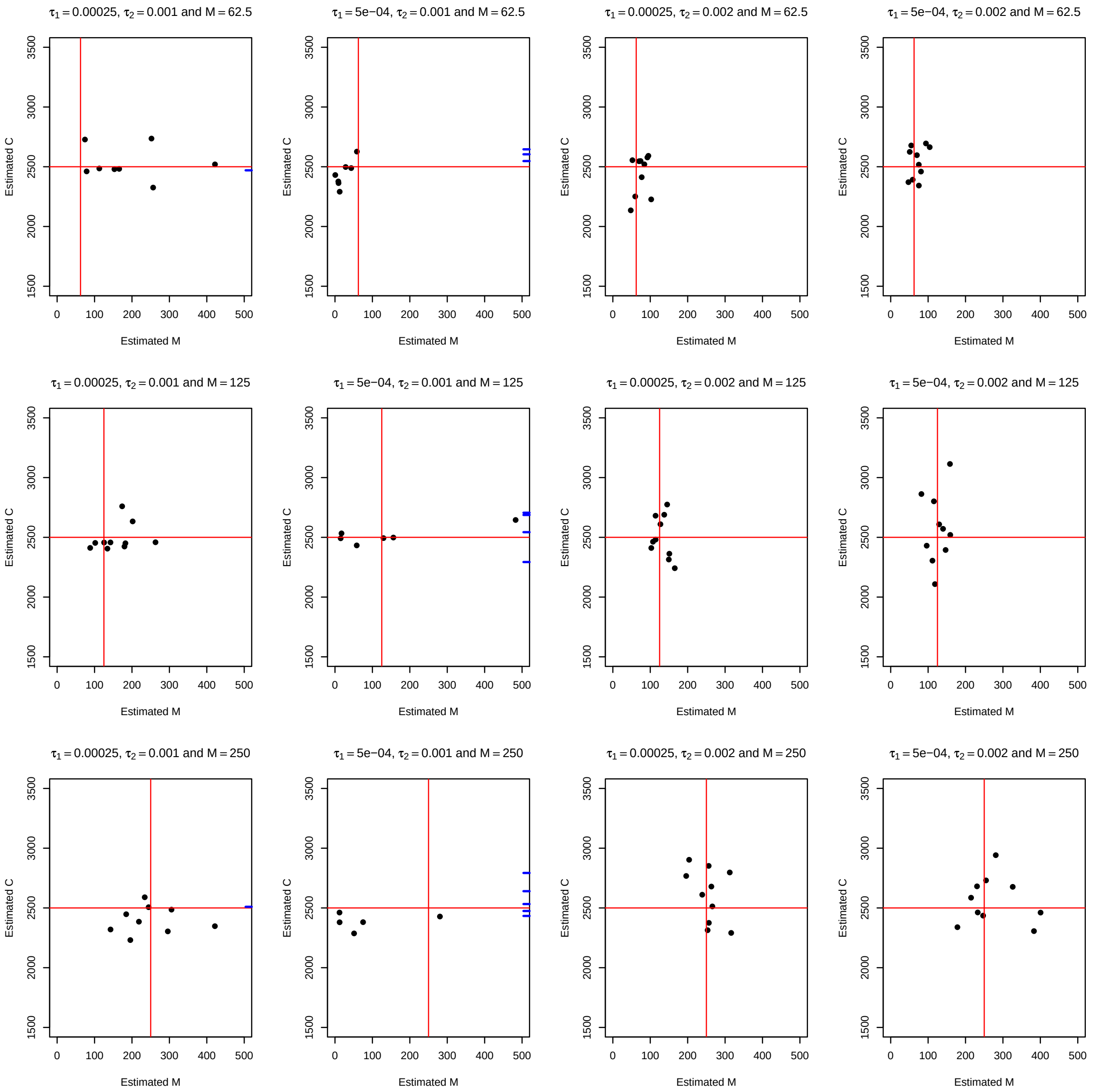

Figure 11. Joint estimates of C and M. For each combination of simulated values for τ_1, τ_2, and M, the plots show the joint estimates of C and M. The axes are equal on all plots, and when an estimate falls outside of the span of M values show, it is shown as a blue rug.

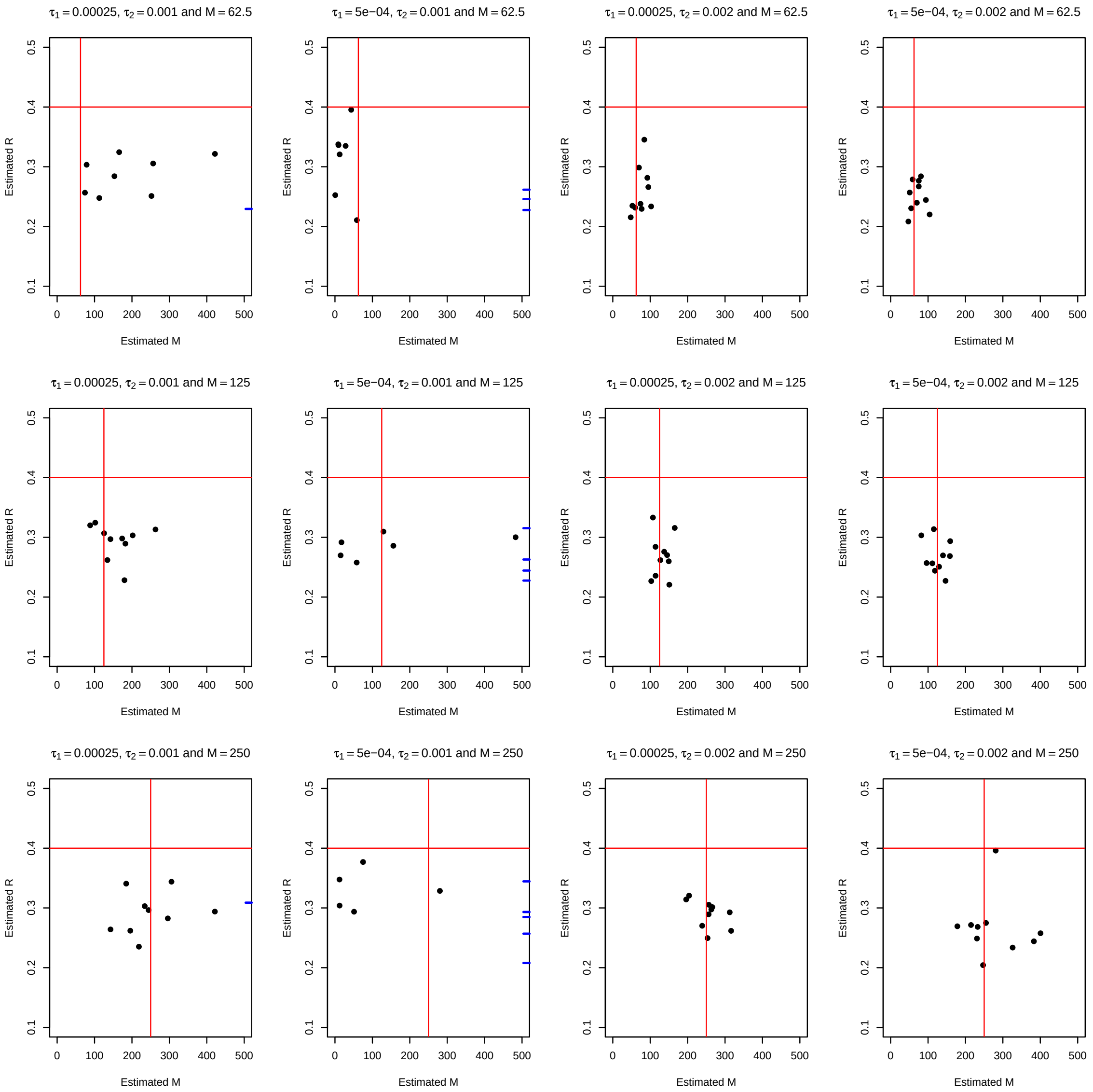

Figure 12. Joint estimates of R and M. For each combination of simulated values for τ_1, τ_2, and M, the plots show the joint estimates of R and M. The axes are equal on all plots, and when an estimate falls outside of the span of M values show, it is shown as a blue rug.

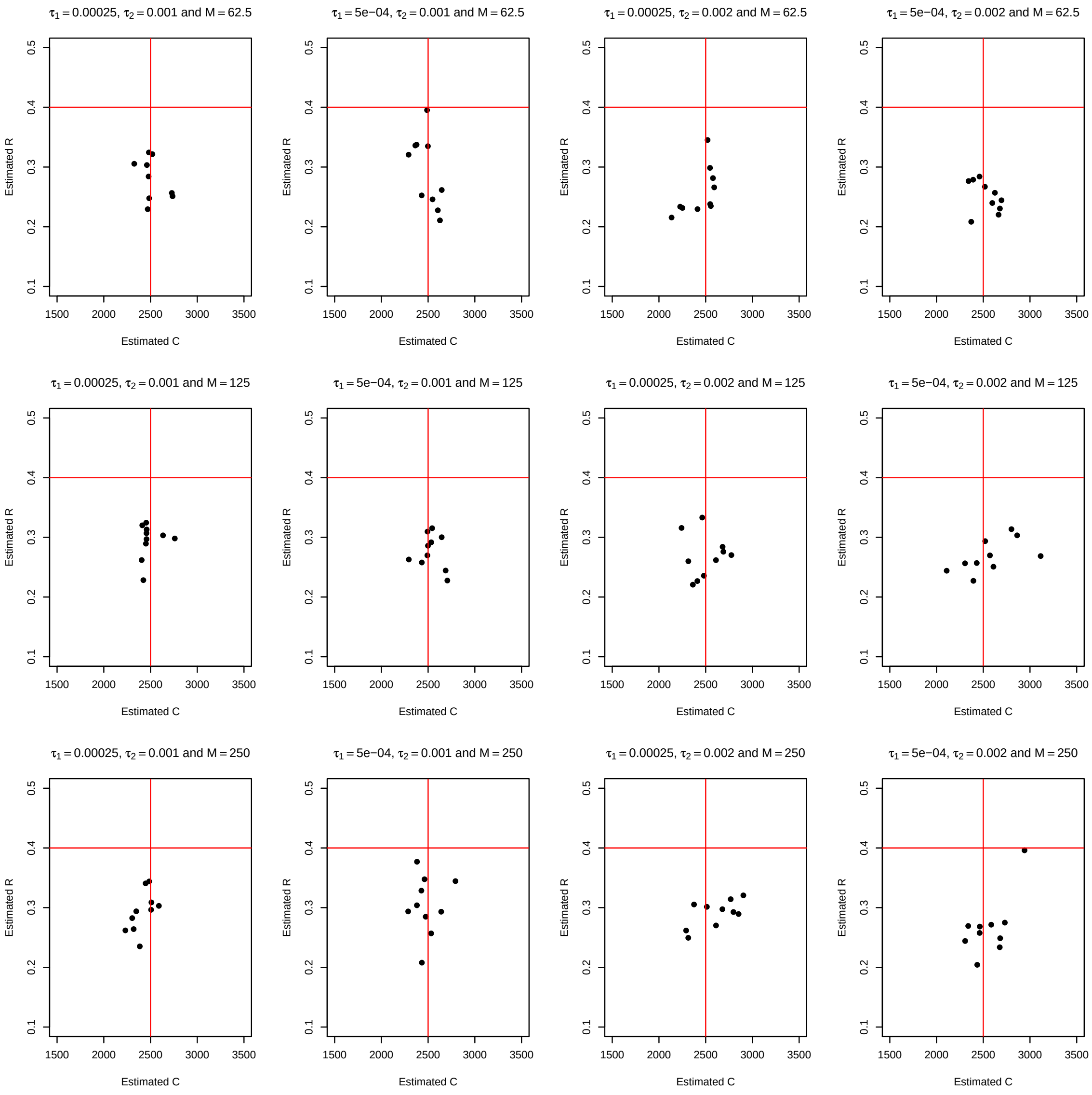

Figure 13. Joint estimates of R and C. For each combination of simulated values for τ_1, τ_2, and M, the plots show the joint estimates of R and C.

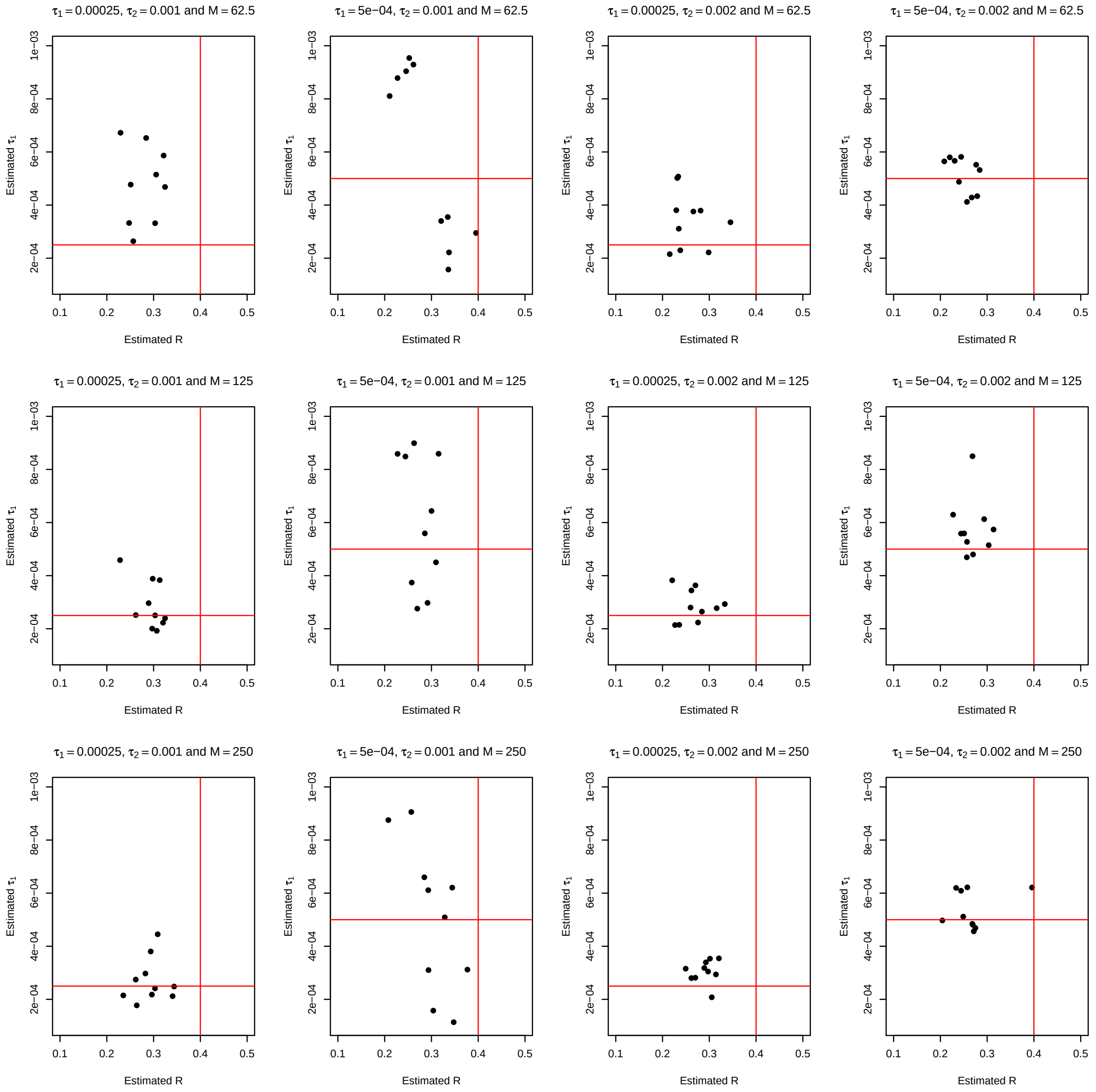

Figure 14. Joint estimates of R and τ_1. For each combination of simulated values for τ_1, τ_2, and M, the plots show the joint estimates of R and τ_1.

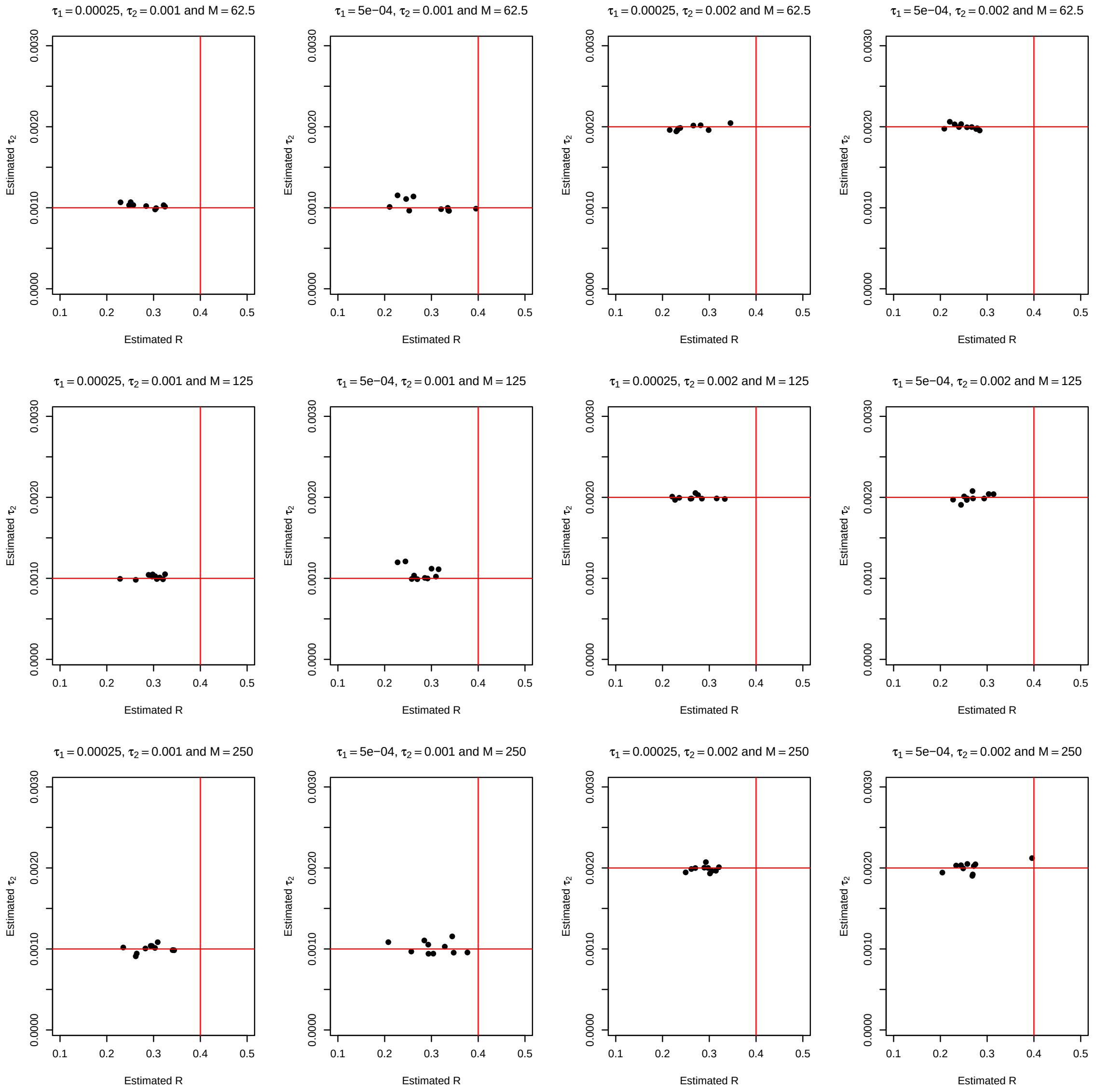

Figure 15. Joint estimates of R and τ_2. For each combination of simulated values for τ_1, τ_2, and M, the plots show the joint estimates of R and τ_2.

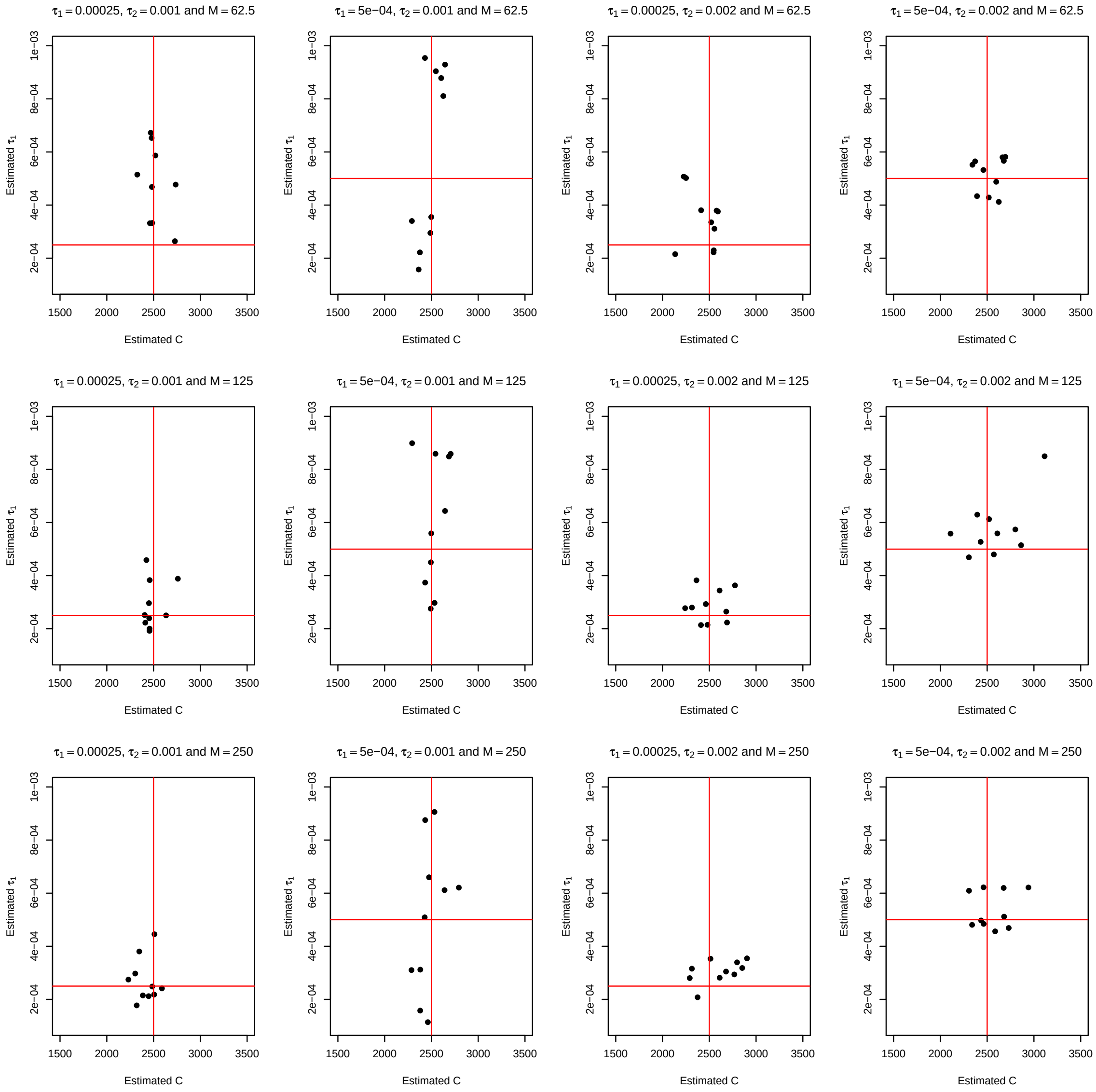

Figure 16. Joint estimates of C and τ_1. For each combination of simulated values for τ_1, τ_2, and M, the plots show the joint estimates of C and τ_1.

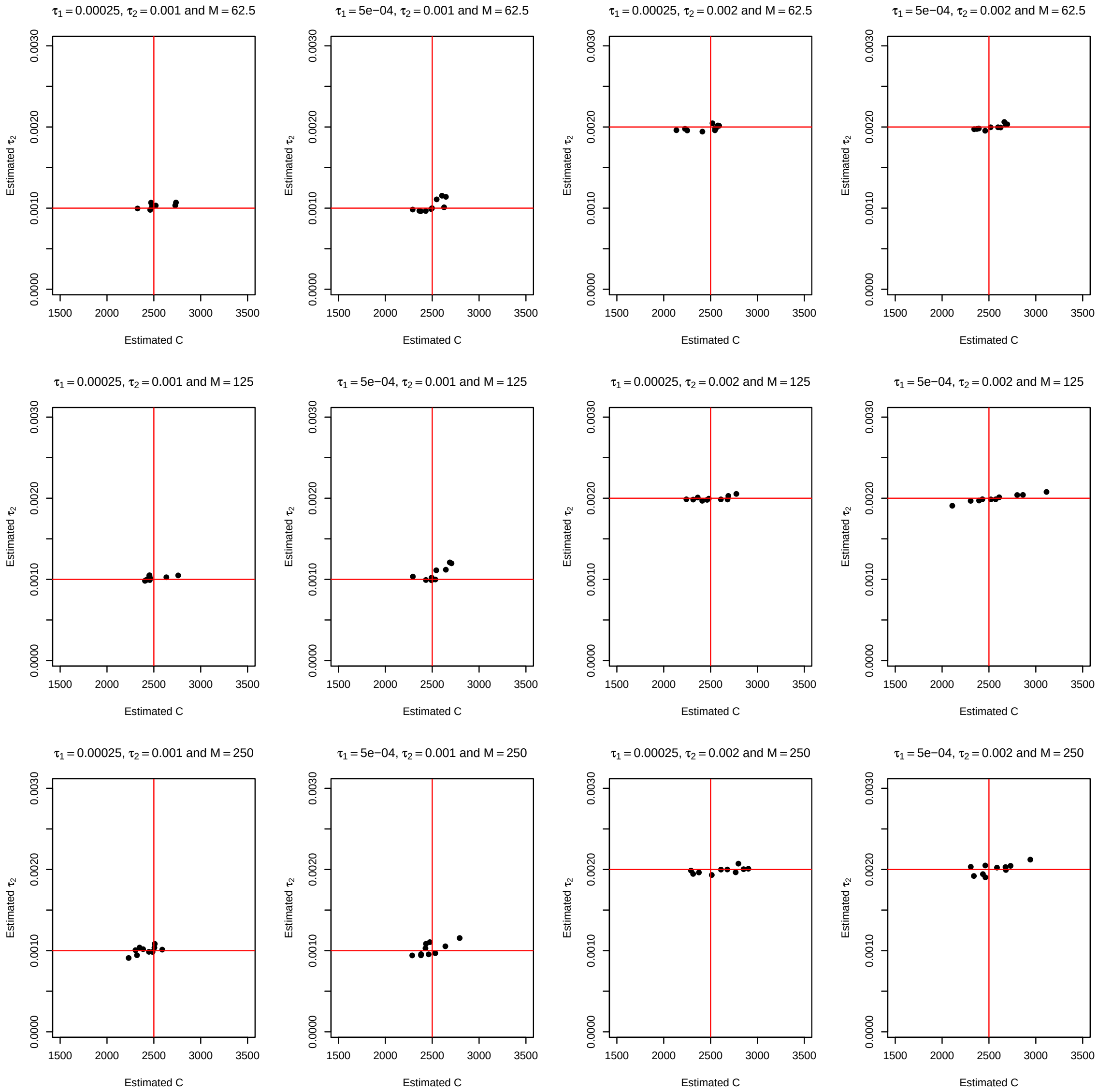

Figure 17. Joint estimates of C and τ_2. For each combination of simulated values for τ_1, τ_2, and M, the plots show the joint estimates of C and τ_2.

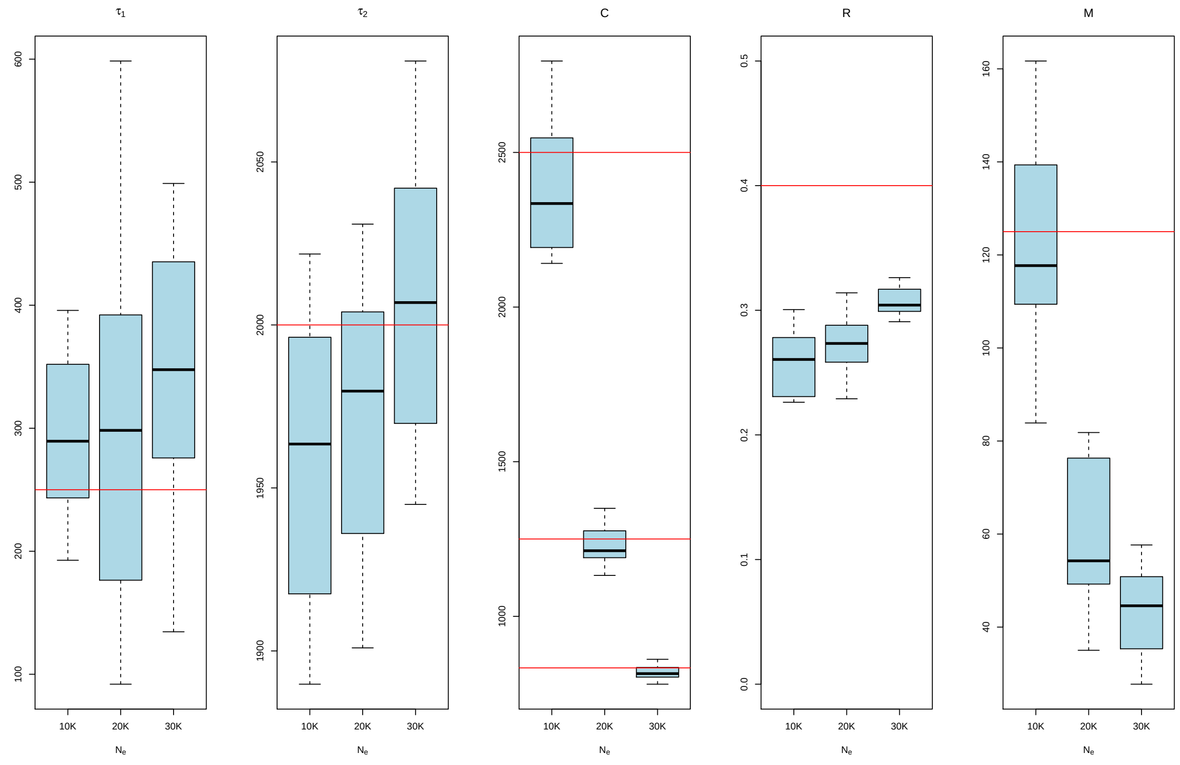

Figure 18. Parameter estimates with varying coalescence rate. Parameter estimation when the coalescence rate varies to match an effective population size equal to 10000, 20000 and 30000.

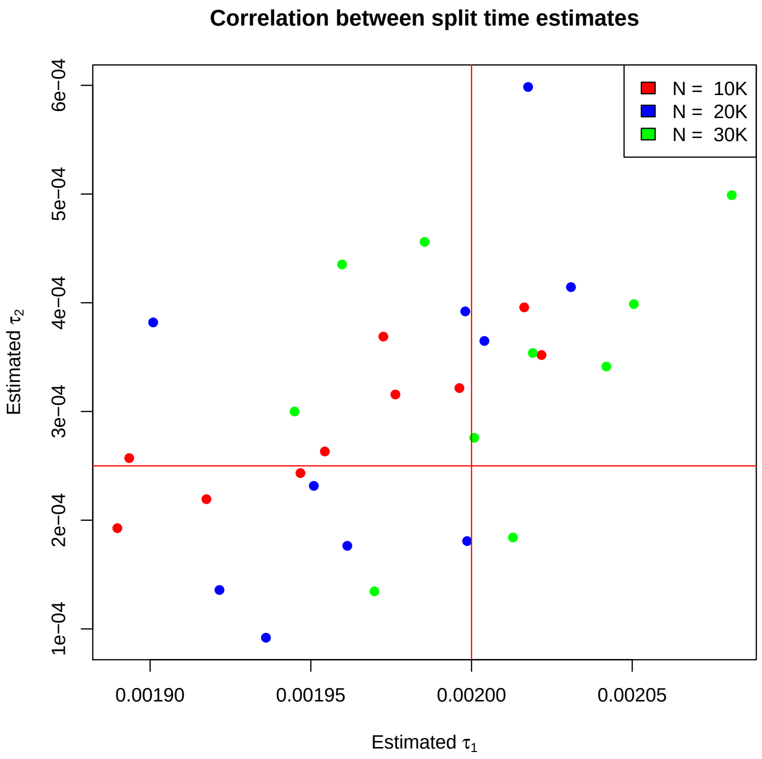

Figure 19. Correlation between split time estimates for varying effective population size. We see a positive correlation between the time split estimates, but this effect does not seem to depend on the effective population size.

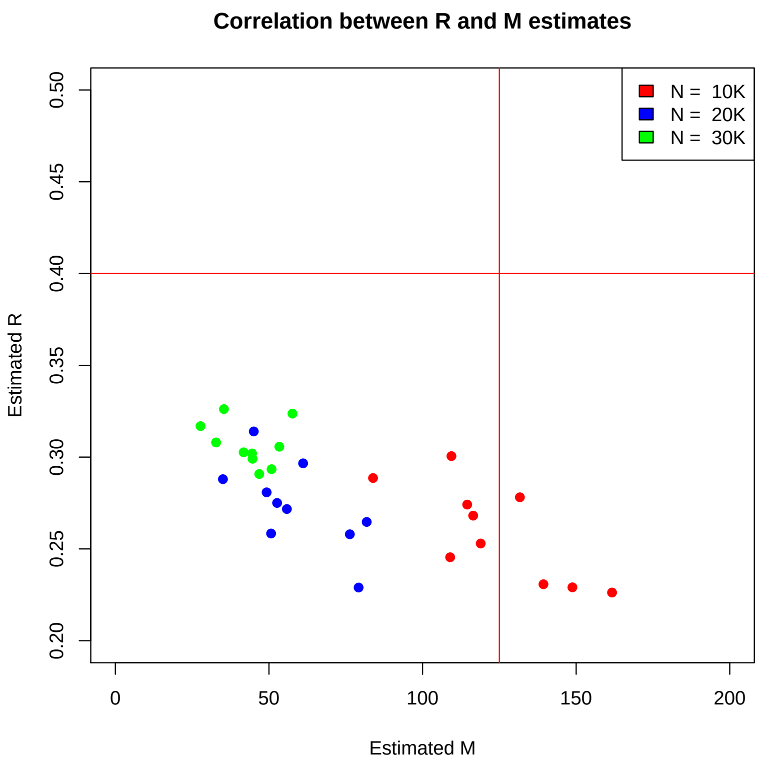

Figure 20. Correlation between migration rate and recombination rate estimates for varying effective population size. Both rate estimates seem to depend on the effective population rate and a slight negative correlation between the two estimates is apparent.

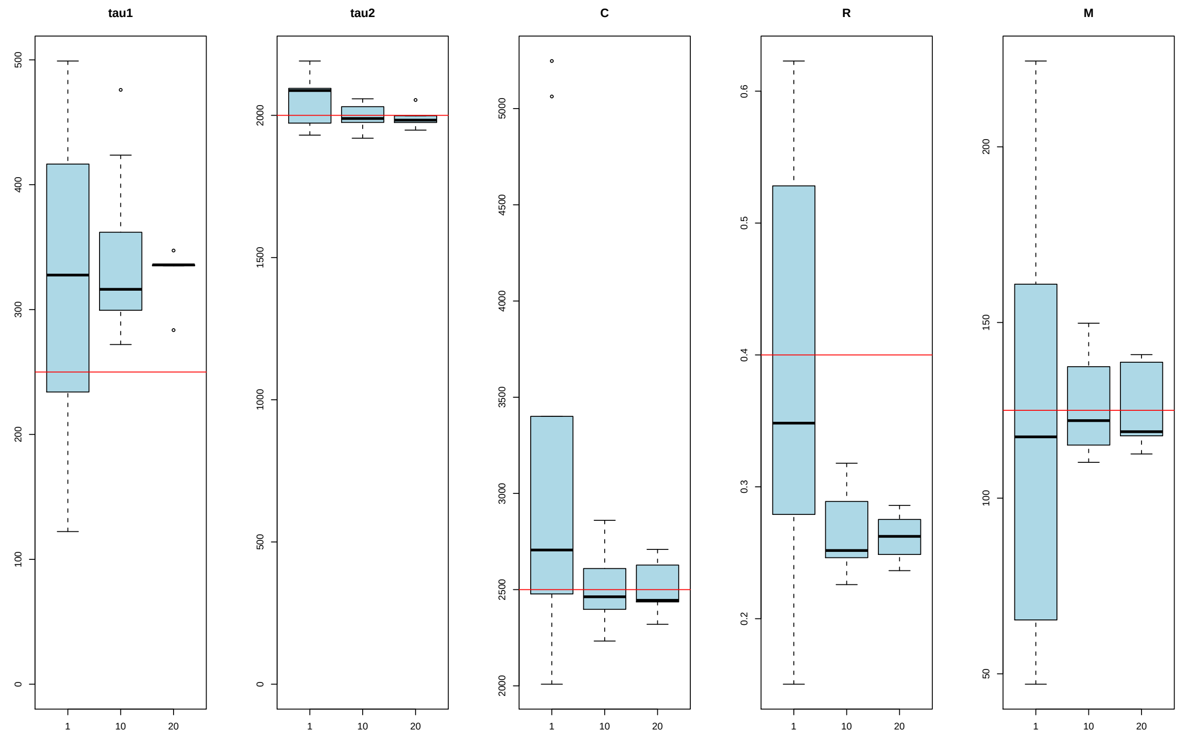

Figure 21. Estimation accuracy as a function of data size. Box plots show the variation in estimates for 10 datasets of length 1, 10 and 20 Mbp.

4 Different coalescence rates

The CTMC underlying the CoalHMM allows for different coalescence times in the different populations, however we found in our previous isolation model that we generally were not able to infer the effective population sizes in the extant populations [3]. For the isolation model, there are no coalescence information about the extant populations and the only information in the data related to the extant effective population sizes is the length of segments with the same coalescence rate that is partly determined by these, and we found that there was not enough information in this to make inference of these parameters.

With the isolation-with-migration model, there *are* coalescence events in the separate populations and thus theoretically more information about the effective population sizes there. An argument from symmetry tells us that we cannot hope to infer these parameters in all cases. The coalescence times and the fragment sizes more recent than the initial population split depends on the two coalescence rates, the recombination rate, and the migration rates. The model is completely symmetric in the populations, so flitting C_1 and C_2 and at the same time M_{12} and M_{21} will give exactly the same coalescence patterns. At best, then, we can hope to make inference of the extant population coalescence rates up to symmetry.

As shown in Figure 22 we generally cannot even do this. The likelihood surface, where an example is shown in the figure, typically shows a linearity between the two coalescence rates, so as a general rule we will likely underestimate one parameter and overestimate another.

Figure 23 shows the result of trying to estimate three free coalescence rate parameters, and clearly shows the linearity problem with C_1 and C_2 plus the difficulty we have with estimating these two parameters. The only reason that the C_1 estimates are higher than C_2 estimates here is that we start the optimization with C_1 lower than C_2, had we reversed this the estimates would have changed as well and we would be even worse at estimating the parameters.

Since we cannot estimate C_1 and C_2 accurately, we prefer to keep the number of parameters down and estimate setting all the coalescence rates to be equal. Even when the actual parameters are simulated to be different, this gives us tighter estimates of C_A as shown in Figure 24. We do have a slightly bias in overestimating the parameter, but this seems to be the case whether the parameters are fixed or free, and is also observed when we have simulated the coalescence rates to be equal.

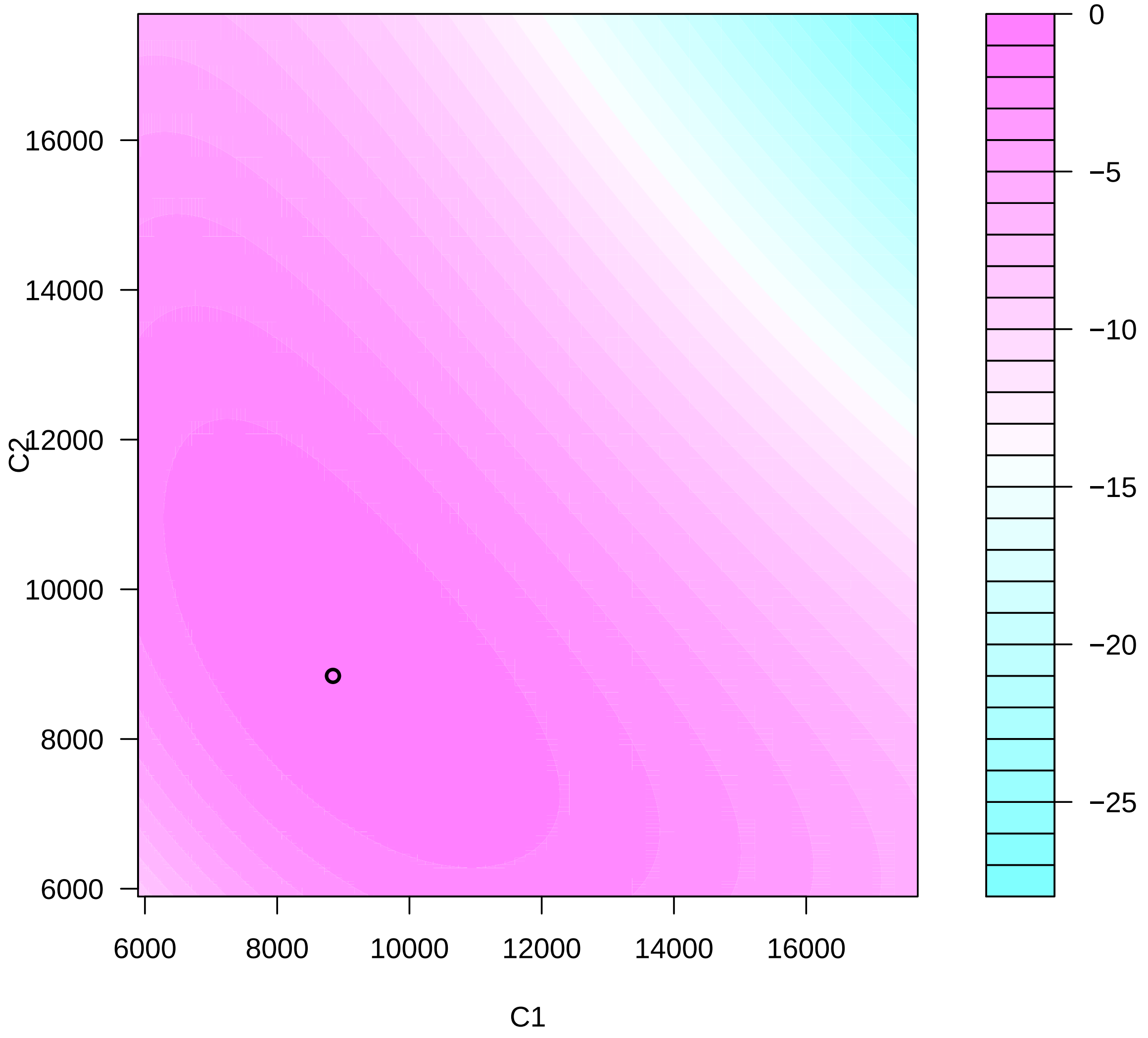

Figure 22. Log likelihood surface for C_1 and C_2. The likelihood surface shows the linearity between C_1 and C_2 in the model that makes it impossible to estimate these parameters independently, all else being equal.

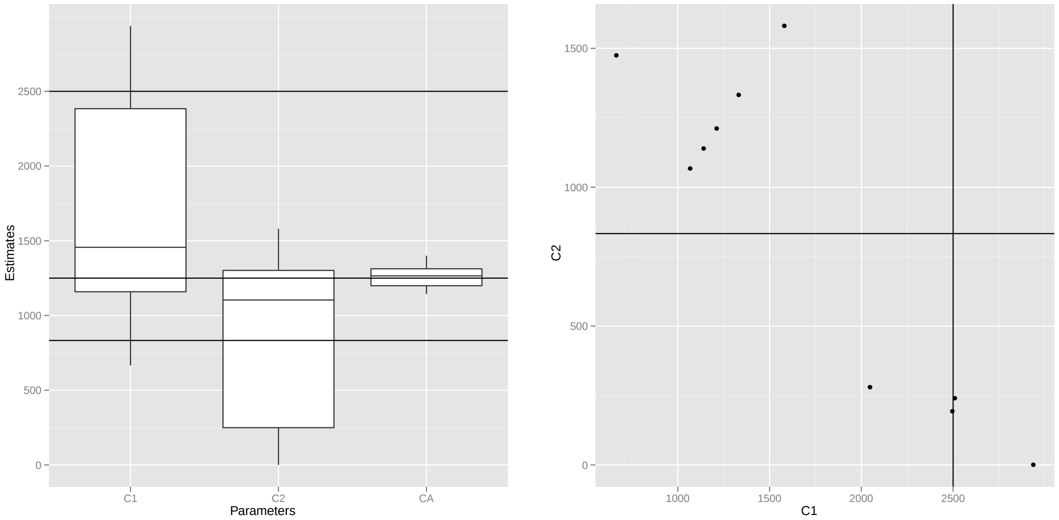

(a) Estimates for free Cs (b) Correlation for estimates of C_1 and C_2

Figure 23. Estimates when all Cs are free to vary. Data was simulated with $C_1 = 2500$, $C_2 = 833$ and $C_A = 1250$. On the left is shown box plots for the parameter estimates and on the right the correlation between estimates for C_1 and C_2. The horizontal and vertical lines show the simulated values.

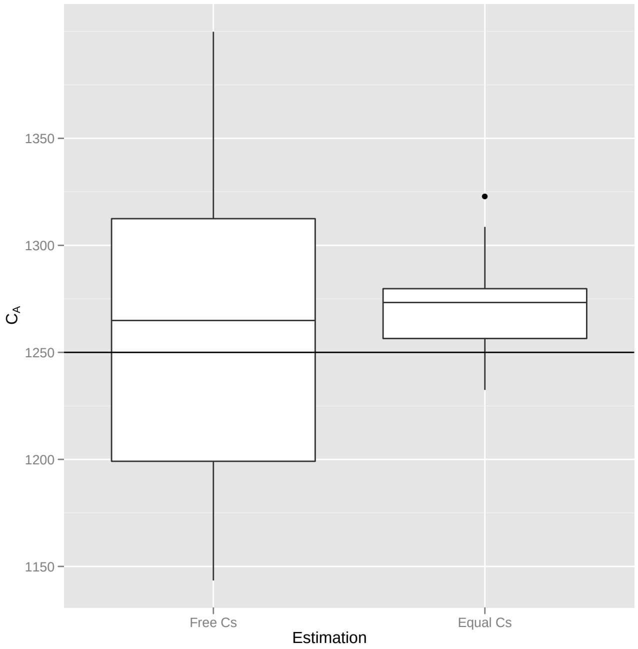

Figure 24. Comparison of estimates of C_A when we let the Cs vary freely or when we fix all Cs to be equal. When we hold all coalescence rates to be equal, we get a tighter estimate for C_A but have the same slight upwards bias in estimates.

5 Asymmetric migration

Our model allows asymmetric migration rates, and to test our estimation accuracy with this we simulated data where we varied the split times as before but kept migration in one direction at 62.5 and the other direction at 250.

Whether we estimate with symmetric or asymmetric migration doesn't seem to have a major impact on the other parameters where we get very similar results from both estimation models, see Figure 25. Therefore, we would not expect biases in those parameters if we use a symmetric migration rate in estimation even if the true migration pattern was asymmetric.

As for the migration parameters, if we estimate using the model with symmetric migration, the estimated migration rate falls between the actual, asymmetric, migration rates, capturing the mean migration rather than the directional migration, see Figure 26. Estimating with asymmetric migration parameters, we have a higher variance on the estimates – not surprising when we increase the number of parameters to estimate – but on average we tend to recover the parameters.

The box plot summary of estimate recover is deceptive, however, as can be seen on Figure 27. Very few estimates are around the right M_{12} and M_{21} at the same time; most of the time one parameter is over-estimated while the other is under-estimated. The problem is symmetry in the model very similar to the symmetry there is in coalescence rates, and in general we do not expect to be able to recover migration

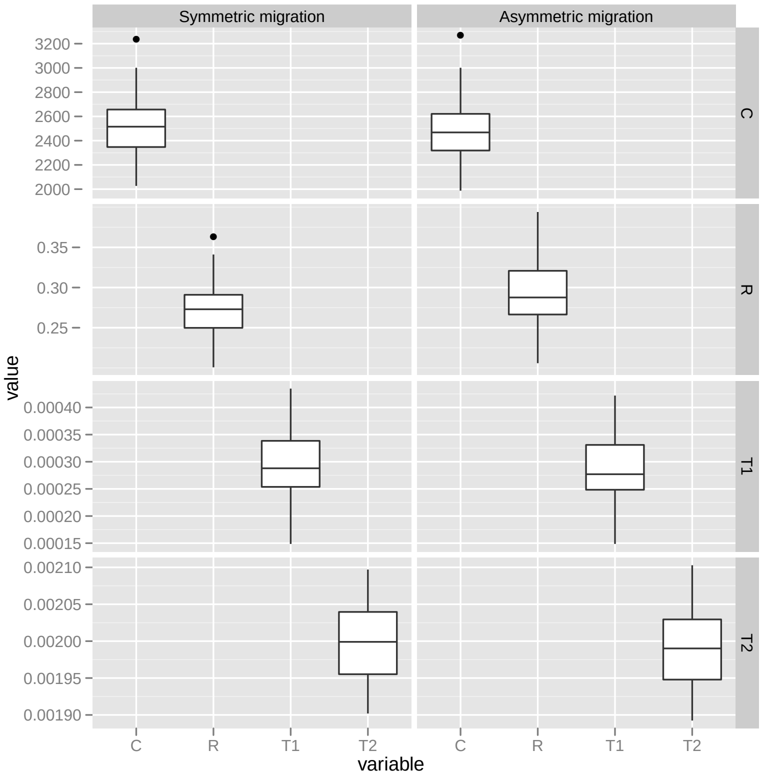

Figure 25. Estimating other parameters with asymmetric migration. Estimates of the other model parameters when data is simulated with asymmetric migration ($M_{12} = 62.5$ and $M_{21} = 250$) and estimation is done with and without asymmetric migration parameters.

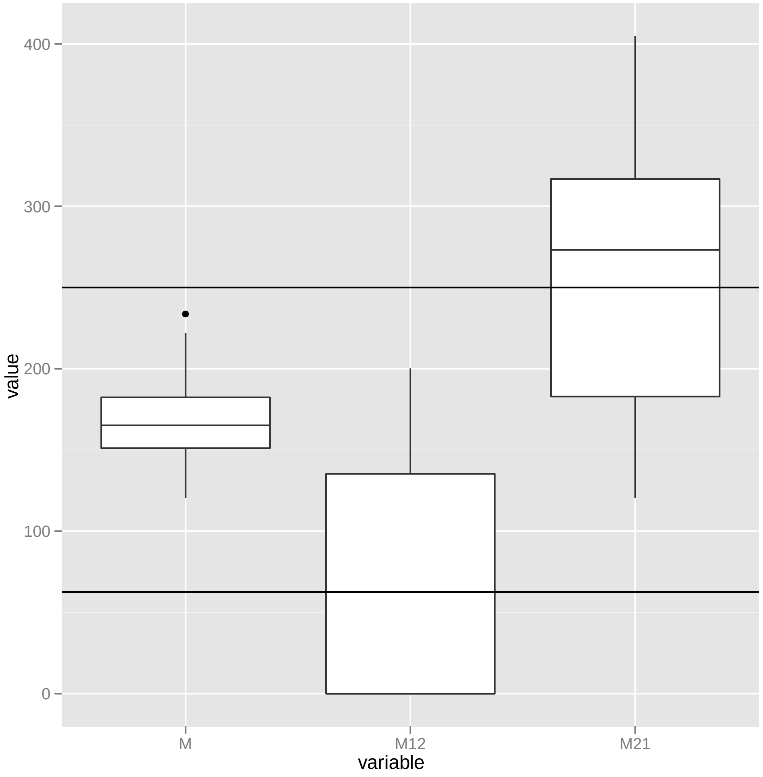

Figure 26. Estimating asymmetric migration parameters. Estimates of migration rates when migration is asymmetric between populations. Boxplots shows the distribution of estimates while the horizontal lines shows the true values for parameters M_{12} and M_{21}. Parameter M shows the estimates when we use the model with symmetric migration to estimate data simulated with the asymmetric migration rates.

rates when migration is asymmetric. The best we get is a mean migration rate when estimating with a symmetric migration parameter, but fortunately estimating with a symmetric migration does not bias the estimates of the remaining parameters.

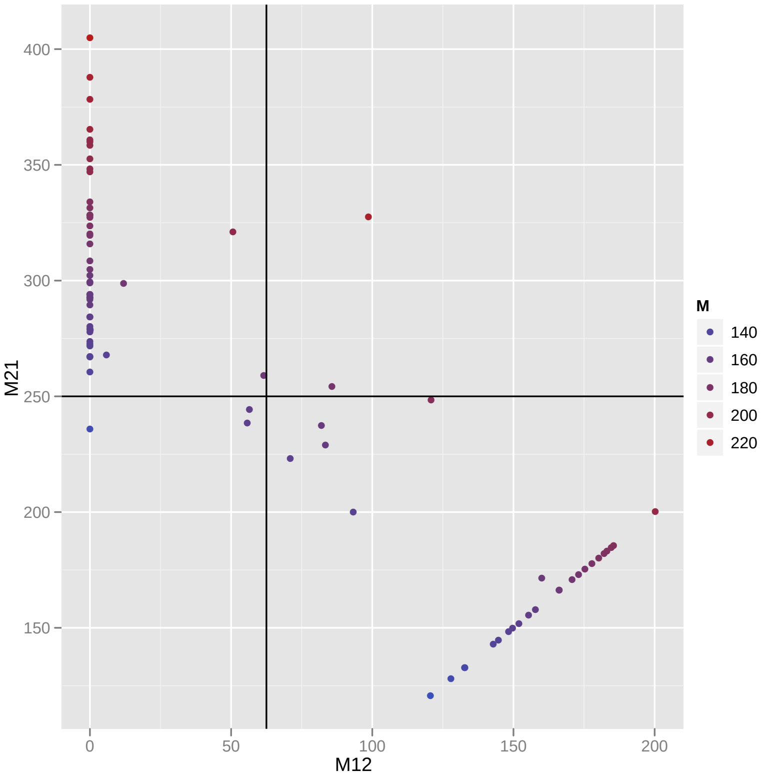

Figure 27. Estimating asymmetric migration parameters. Correlation between M_{12} and M_{21} estimates, with the symmetric M parameter shown color-coded, showing that M in some way captures a mean of the other two with higher values towards the upper right and smaller values towards the lower left.

6 Robustness to model assumptions

The previous experiments show how the HMM approximation to the coalescent process performs for parameter estimation, but it is important to keep in mind that also the coalescence model is a simple, perhaps simplistic, model of the real genetic process behind the ancestry of two genomes.

In this section we explore how real data can be expected to deviate from the model assumptions and how this will affect our estimates. While we believe that our model can be extended to explicitly model many of the evolutionary features we describe in this section, we consider this beyond the scope of this paper, and simply wish to explore how the violations of model assumptions can bias our estimates.

6.1 Variation in mutation rate

Our model assumes that the mutation rate is constant across the alignment, and that variation in divergences is entirely determined by variation in coalescence time and stochasticity in the mutation process. We know, however, that the mutation rate is also determined by genomic features and will vary along a real genome alignment.

The way the mutation rate varies is likely to be a complex process, but we model it in our simulations in a rather simple way: We split our alignment into segments, where each segment has a length geometrically distributed (with mean 500 bp, 1000 bp, 1500 bp or 2000 bp), and then we scale the mutation rate in each segment by a random amount.

Results are shown in Figure 28. The result of varying the mutation rate in this fashion is a reduction in the estimated coalescence rate, corresponding to an over-estimation of the effective population size. This is perhaps as expected, considering that the variation in mutation rate is likely to be seen as an increase in the variation of coalescence times by the model, which it will fit by increasing the effective population size.

When decreasing the coalescence rate, the model also decreases the split time (and to a much smaller degree the end of gene-flow). This is because the variation in mutation rate is only seen as an increase in the coalescence time variance and not the coalescence time mean, so the mean divergence of the species still needs to fit the same value; the model just sees more of the divergence time as within the coalescence process rather than divergence between the species.

Not surprisingly the recombination rate estimates also goes up, when the model sees changes in mutation rate as changes in coalescence times, i.e. it sees more coalescence time fragments and therefore more recombination points.

If we vary the length distribution for segments with the same mutation rate, we see a reduction of the affect of variable mutation rate on the estimated recombination rate. When the segment lengths are longer, the model doesn't see them as changes in coalescence times to the same degree and the recombination rate does not go up since the changes in divergence doesn't change unusually much when the mutation rate is constant for longer stretches. The coalescence rate is still under-estimated due to the larger variance in coalescence times, though, and we see the same effects in divergence time parameters for short and long segment sizes.

We also see the migration rate estimates go up when we vary the mutation rate, with a large effect when we vary it in long segments. This is caused by the segments where the mutation rate is scaled down, where a long stretch of the alignment has no or few divergent sites. These will be interpreted by the model as very recent coalescence events that would require migration, and when we see more of such segments, the model estimates a higher migration rate.

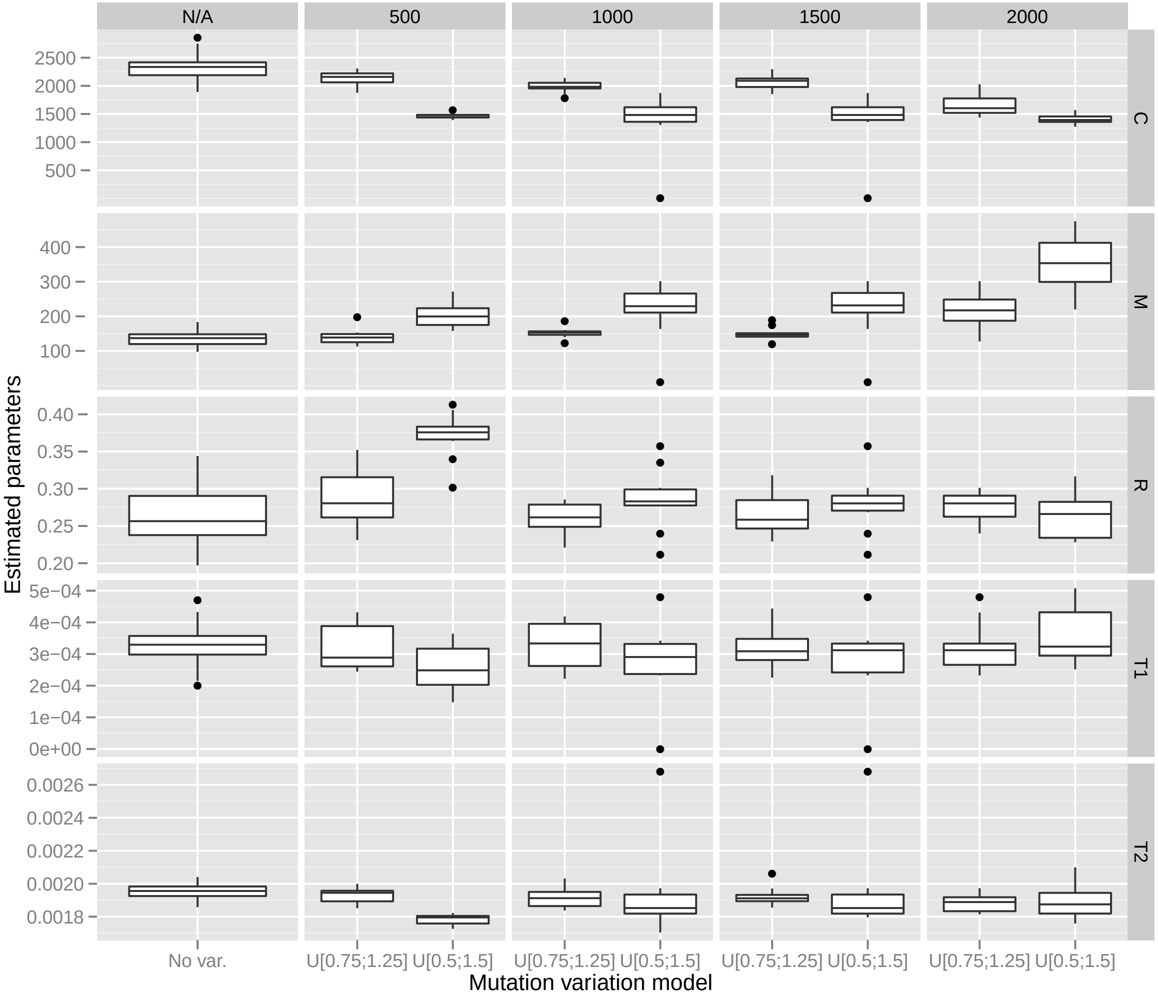

Figure 28. Robustness to variations in the mutation rate across the alignment. The box plots show the consequence of varying the mutation rate in segments along the genome for the various parameter estimates. The parameters used for the simulations were $C = 2500$, $M = 125$, $R = 0.4$, $T1 = 0.00025$, and $T2 = 0.002$. Mutation rates were modified by choosing segments geometrically distributed in length and multiplying the rate with a constant. In this plot, segment lengths had mean 500bp, 1000bp, 1500bp and 2000bp and the multiplication constant was chosen uniformly in $[0.5; 1.5]$ or $[0.75; 1.25]$. The left-most facet shows the estimates with no mutation rate variation.

6.2 Variation in recombination rate

The model assumes that recombination is occurring at a constant rate across the alignment. We know, however, that recombinations primarily occur in hotspots in ape genomes. To test the behavior of the model when recombinations occur in hotspots we simulated data with variable recombination. We picked random 10Mbp segments of the human genome and selected recombination rates corresponding to the recombination rates in the DeCODE recombination map and simulated according to this. A comparison between data with a constant recombination rate and a variable recombination rate is shown in Figure 29.

The main effect we see is that the estimated recombination rate is further underestimated. This is not surprising since the same whole-alignment recombination rate, when isolated to short segments along the sequence, leads to a smaller rate per nucleotide pair, which is what the model estimates. This does mean, however, that one should be careful with the interpretation of this parameter when analyzing alignments where recombination hotspots are present.

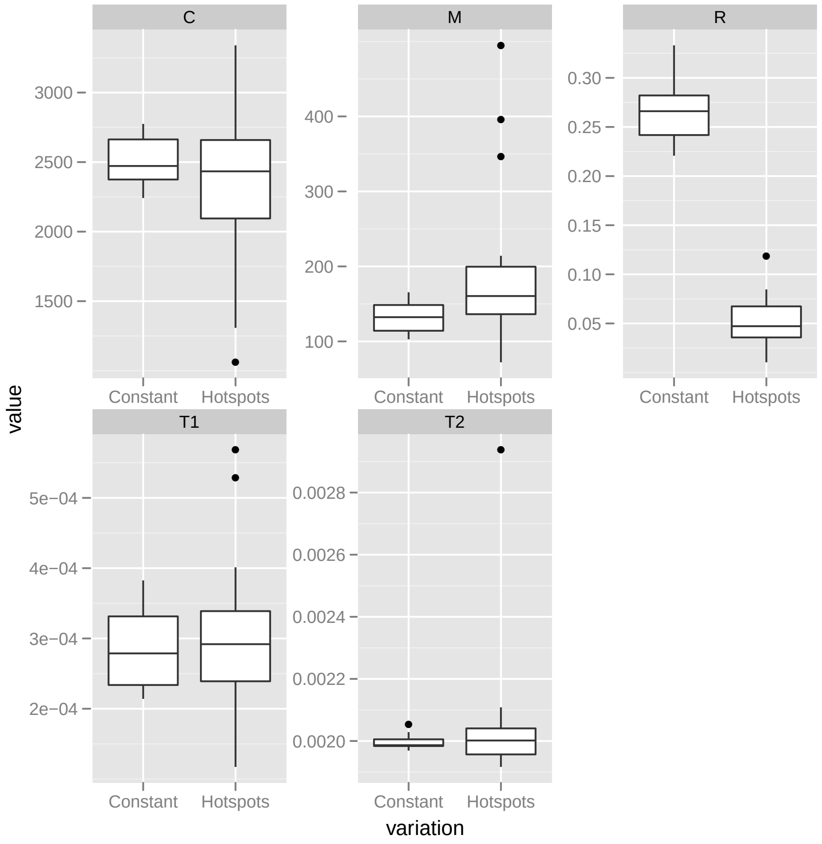

Figure 29. Robustness to variations in the recombination rate across the alignment. The box plots compare parameter estimates when data is simulated with or without recombination rate variation (hotspot structure). For most parameters, we see an increased variance when hotspots are present but no particular bias. The exception is the estimated recombination rate, that is even more underestimated than for the constant rate case.

7 Known and unknown genotype phase

Our model assumes that we analyze a haploid genome, but generally we cannot get such genomes from sequencing data. Instead, we will get a diploid genome with unknown phase. If the species are sufficiently divergence, then we do not expect the phasing of genomes to matter, since both variants will then typically be equally divergent and a random allele will suffice. On the other hand, when some variation is shared between the species, one of the variants could be closer to the other species than the other, and in this case using a random phase could potentially affect the estimates.

To test the effect of this, we simulated two genomes from each species in our coalescence simulations, and for all heterzygotic sites we picked one allele at random to construct a "random" phased genome. In Figure 30 we compare the effect of knowing the phase versus using a random phase for both the isolation and isolation-with-migration model. Overall, we see no major effect in the estimates from not knowing the phase, except for the most recent divergence time, τ_1, in the IM model. This might be because this is the place in the model where using a mix of variants from the two simulated genomes is most seen.

To explore how divergence impacts the effect of not knowing the true phase, we used an isolation model and looked at different levels of species divergence, see Figure 31. As seen, for recently diverged species not knowing the true phase leads us to underestimate the divergence time and overestimate the coalescence rate. When the species are sufficiently diverged, and shared polymorphism is unlikely, this effect disappears.

With an IM model, the effect of a recent end of gene flow, a low τ_1, should be even less influential on the parameter estimates than a low split time in an I model, since even with a recent end of gene flow most coalescences will be further back in time and the amount of shared polymorphism therefore also much smaller. Figure 32 illustrates this, where parameter estimates are shown against the percentage of the polymorphism that is shared between two diploid genomes. Here, only the estimates of the end of gene flow seems much affected, and only by a slight over estimates for the most recent simulated time.

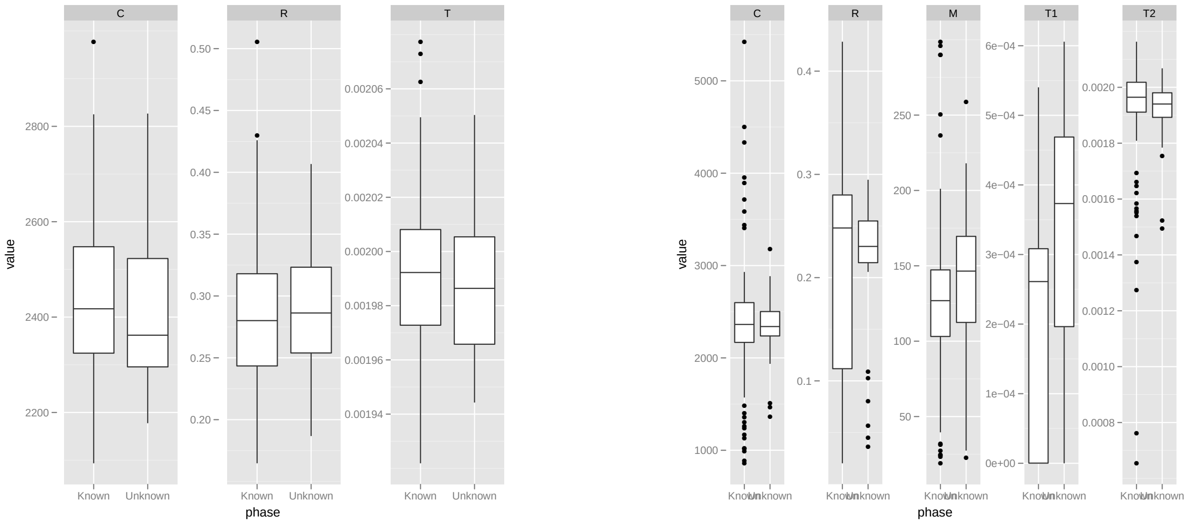

(a) Isolation model

(b) Isolation-with-migration model

Figure 30. Effect of knowing the phase or using a random phase. The plot shows the parameter estimates when we either know the true phase of the genomes, or when we have assigned a random phase to all heterozygotic sites.

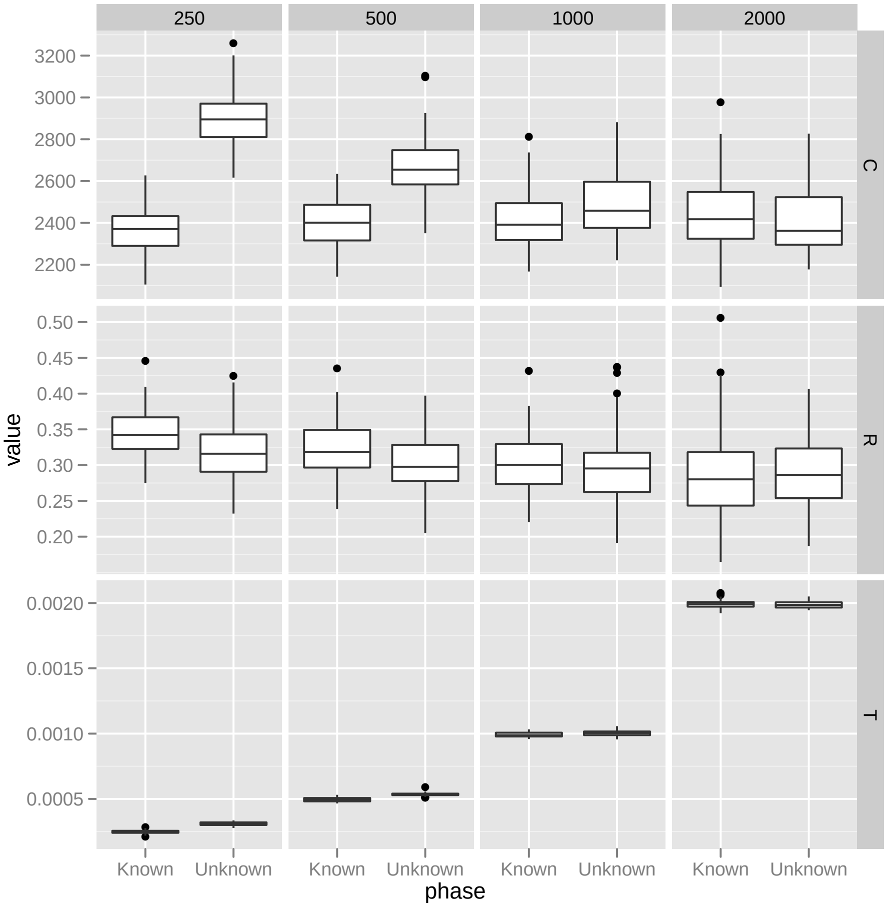

Figure 31. Effect of knowing the phase or using a random phase for different divergence times. The plot shows the parameter estimates when we either know the true phase of the genomes, or when we have assigned a random phase to all heterozygotic sites, for different divergence times in the isolation model. Divergence times are shown on the facets on the x-axis (in thousands of years).

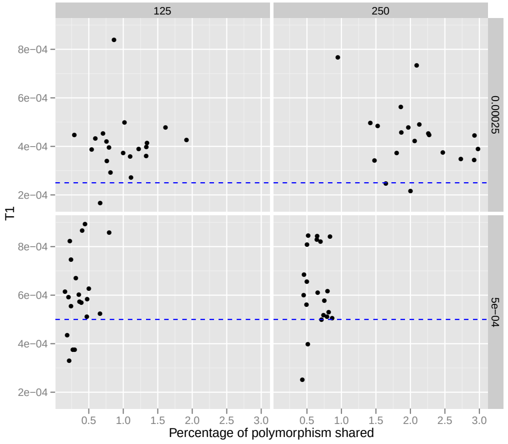

(a) Estimates of τ_1

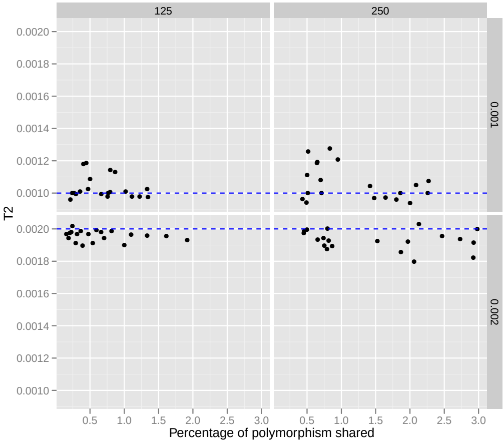

(b) Estimates of τ_2

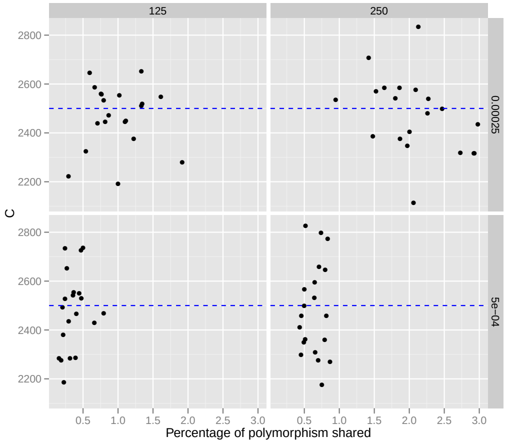

(c) Estimates of C

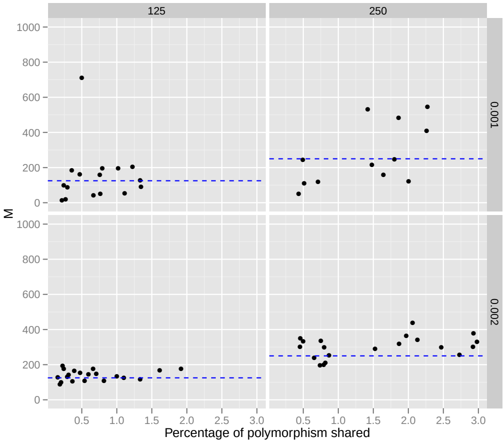

(d) Estimates of M

Figure 32. Effect of shared polymorphism on estimates when using a random phase. The plot shows the parameter estimates as a function of the percentage of polymorphism that is shared between populations, when using a random phase of diploid genomes. Columns of the facets corresponds to the simulated migration rate, $M = 125$ or 250, while rows corresponds to either $\tau_1 = 250$ kya or 500 kya or $\tau_2 = 1$ mya or 2 mya.

8 Model checking

A pure isolation model is obtained by setting the migration rates to zero, and so we can immediately compare the likelihoods of isolation versus isolation-migration models to build hypothesis tests for migration.

To get a feeling for how this would work, we simulated data with and without migration and then performed maximum likelihood estimation using models with and without migration. Figure 33 and Figure 34 show the maximum likelihood in each step of the numerical optimization for data simulated under an isolation model and an isolation-with-migration model, respectively. The y-axis shows the log likelihood in all evaluations of the model and is therefore not monotonically increasing, but the figures give an idea about how we approach the maximum in the optimization. In general, the optimization requires many more steps for the IM model, not surprising as there are more parameters to optimize, but for data simulated under an isolation model, the final maximum likelihood is at roughly the same value for the isolation model and the isolation-with-migration model, while for data simulated under an isolation-with-migration model the isolation-with-migration model reaches a much higher final likelihood.

8.1 Likelihood ratio test

We summarize the likelihood comparisons as $D = -2\ln\left(\frac{L_A}{L_0}\right)$ for different simulation parameters in Figure 37. If the models were nested, we would expect this summary statistics to be χ^2-distributed with two degrees of freedom (since the alternative model, the IM-model, has two parameters more than the null model, M and τ_1). The models are not properly nested since $M = 0$ lies on the border of allowed values for that parameter and as shown in Figure 35 the test statistics clearly does not follow the χ^2 distribution under the null model.

We still find the likelihood ratio summary a useful statistics for comparing the IM model with the I model. In Figure 36 is shown the D statistics plus P-values *assuming* the χ^2 distribution. We here have 7 “significant” results for $\tau = 250$kya and $\tau = 500$kya and zero for the higher split times.

Using the χ^2 test, out of a hundred simulations for each parameter combination, we got the number of significant results shown in the table below.

τ_1 (kya)	τ_2 (kya)	M	No. Significant
250	1000	62.5	94
250	1000	125	100
250	1000	250	100
500	1000	62.5	38
500	1000	125	57
500	1000	250	82
250	2000	62.5	98
250	2000	125	100
250	2000	250	99
500	2000	62.5	100
500	2000	125	100
500	2000	250	100

Except when looking at the shortest migration interval, we seem to generally have a very good power for detecting migration. With the test, but have to keep in mind that we do not have a proper test with the right error rate for a given threshold.

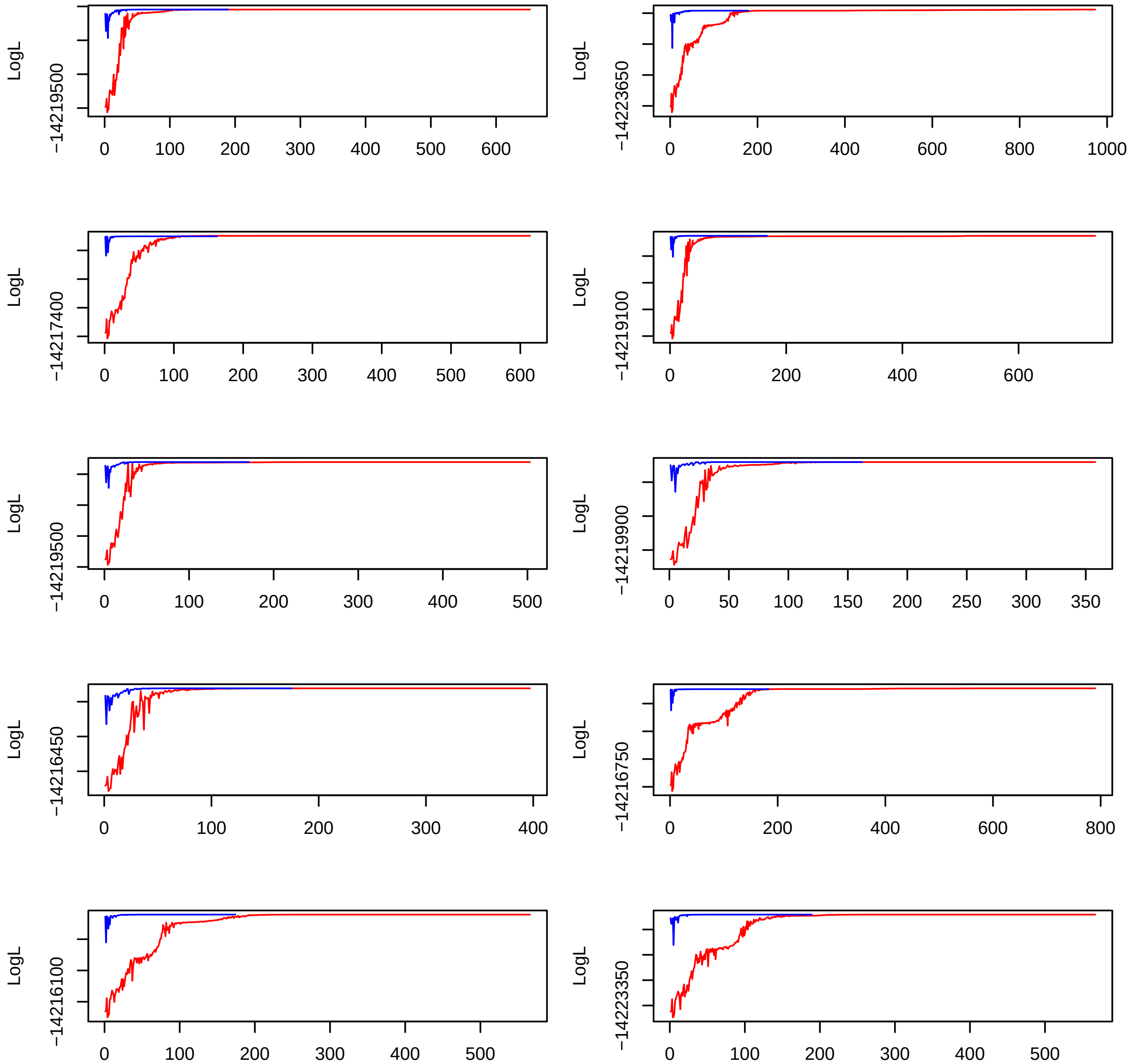

Figure 33. Log likelihoods during the optimization for data simulated under a pure isolation model. The plot shows the log-likelihood at each step in the numerical optimization. Blue lines corresponds to maximum likelihood optimization under the isolation model, while red lines corresponds to optimization with the IM model.

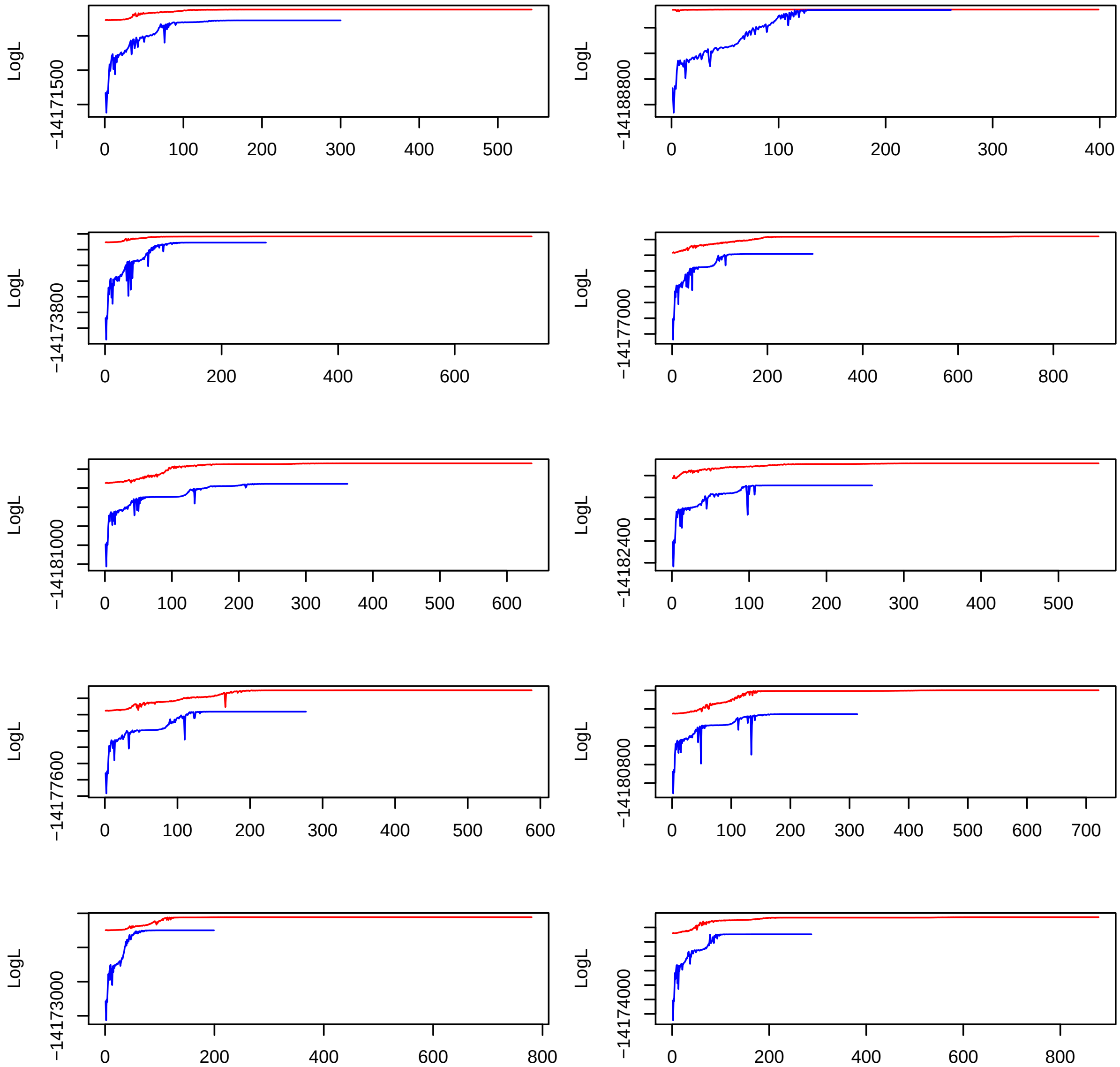

Figure 34. Log likelihoods during the optimization for data simulated under an isolation-with-migration model. The plot shows the log-likelihood at each step in the numerical optimization. Blue lines corresponds to maximum likelihood optimization under the isolation model, while red lines corresponds to optimization with the IM model.

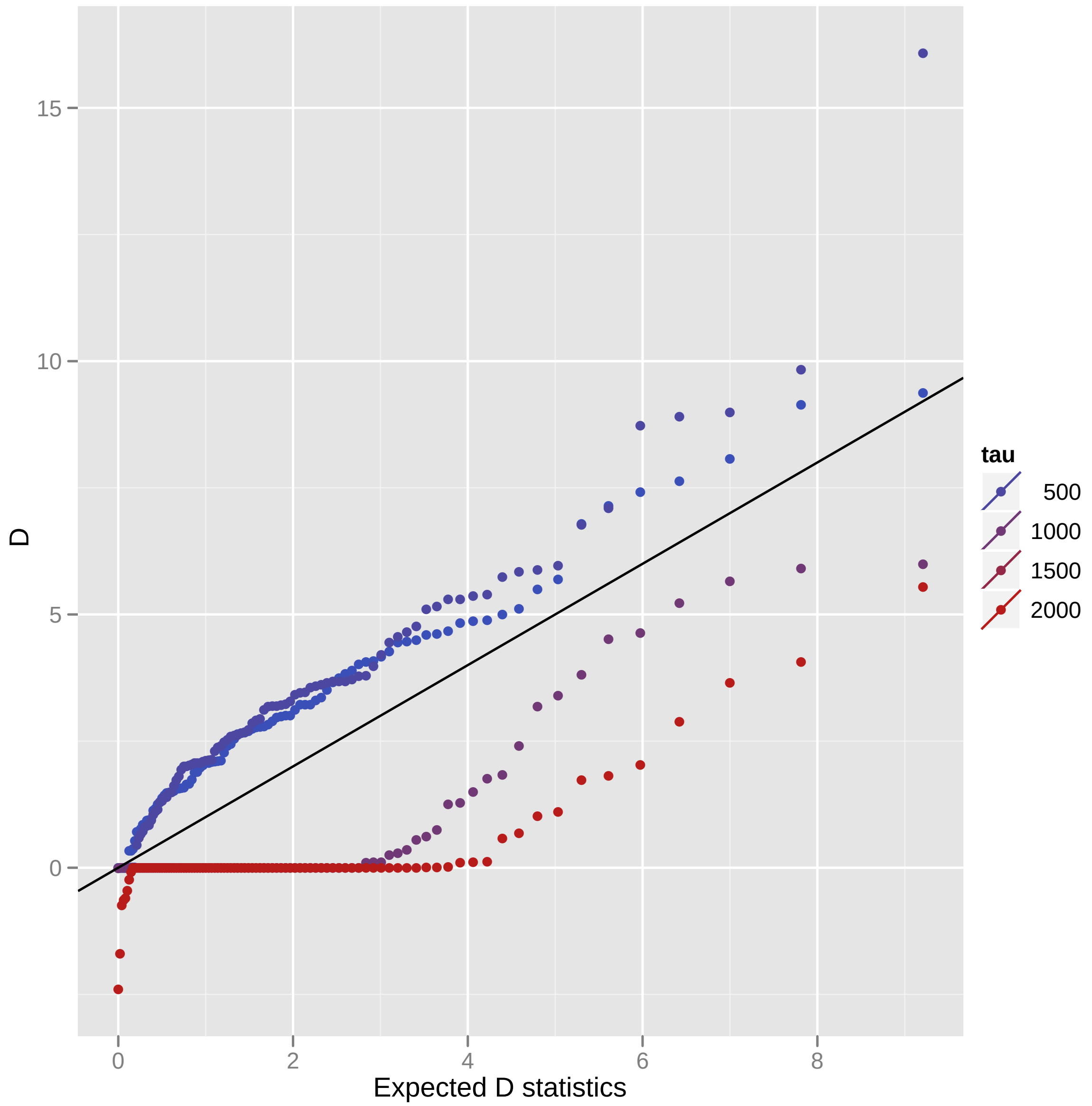

Figure 35. QQ-plot for the migration test under a pure isolation model. If the likelihood ratio test was χ^2 distributed, the D statistics would lie on the $x = y$ line, which they clearly do not. For a recent population split in a pure isolation model, the test would seem to have a higher error rate than 5% while for a more ancient split we would have a much lower error rate. The negative D values for some of the runs are an artifact of the maximum likelihood optimization.

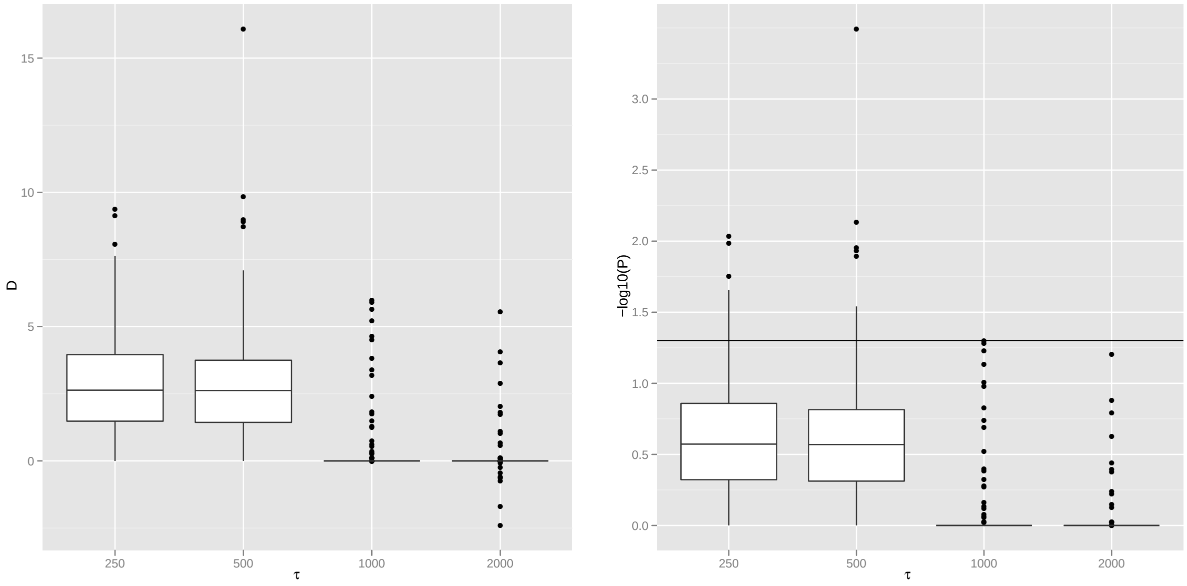

(a) Likelihood ratio statistics

(b) P-values, assuming a χ^2 distribution

Figure 36. Likelihood ratio tests for migration under a pure isolation model. Plots showing likelihood ratio comparisons for testing migration for varying τ (the pure split time). On the left is shown the likelihood ratio statistics $D = -2\ln(L_A/L_0)$ and on the right the corresponding P-values assuming that the D statistics is χ^2 distributed with 2 degrees of freedom under the null-model. Here, the 5% significance threshold is shown as horizontal lines.

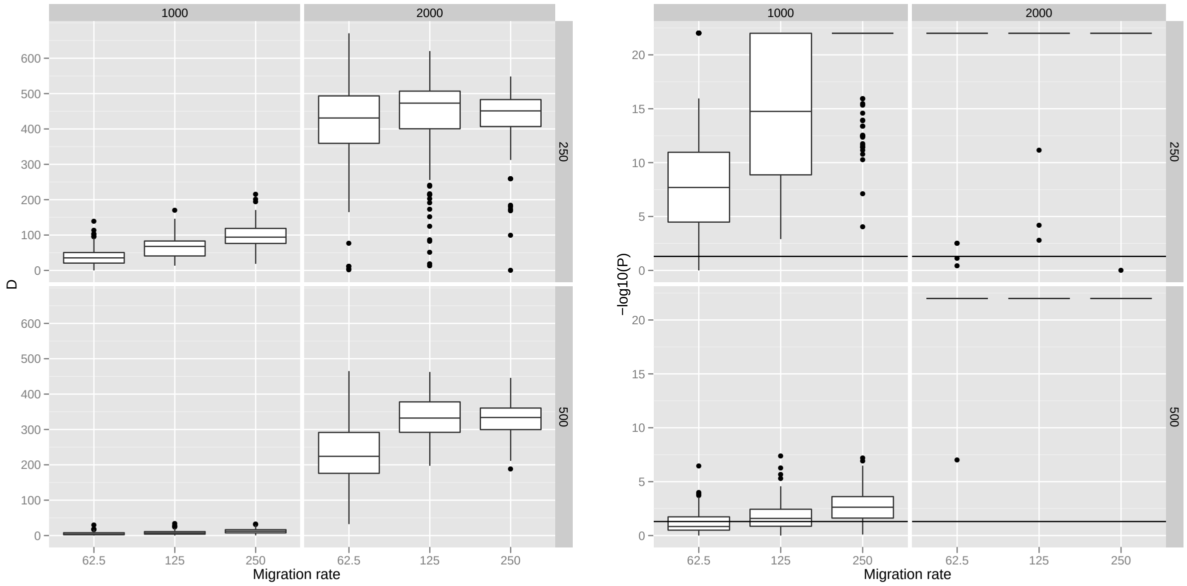

(a) Likelihood ratio statistics

(b) P-values, assuming a χ^2 distribution

Figure 37. Likelihood ratio tests for migration. Plots showing likelihood ratio comparisons for testing migration for varying τ_1 (250 kya and 500 kya), τ_2 (1 mya and 2 mya), and migration rate (62.5, 125, and 250). On the left is shown the likelihood ratio statistics $D = -2\ln(L_A/L_0)$ and on the right the corresponding P-values assuming that the D statistics is χ^2 distributed with 2 degrees of freedom under the null-model. Here, the 5% significance threshold is shown as horizontal lines.

8.2 Akaike's information criteria

Unable to do a likelihood ratio test to differentiate between isolation and isolation-with-migration models, we tried AIC which penalizes the number of parameters used in fitting models but does not require nested models.

For AIC, the model with the smallest score should be preferred, so we looked at the AIC for the isolation model minus the AIC for the isolation-with-migration model. When this is positive, the IM model is preferred while when it is negative the I model is preferred. Figure 38 shows these results for the data presented in Figures 36 and 37. The AIC approach generally picks the right model, i.e. shows value below zero when data was simulated under the isolation model and above zero when data was simulated under the isolation-with-migration model.

When simulating under the isolation model, the AIC clearly prefers the isolation model stronger when the split time is higher and is more likely to prefer the wrong model when the split time is smaller.

τ (kya)	I preferred	IM preferred
250	74	26
500	77	23
1000	93	7
2000	97	3

When data is simulated under the isolation-with-migration model, the AIC prefers this. The larger the separation between τ_1 and τ_2 and the larger the migration rate, the stronger the model selection signal seems to be.

τ_1 (kya)	τ_2 (kya)	M (per subst)	I preferred	IM preferred
250	1000	62.5	4	96
250	1000	125	0	100
250	1000	250	0	100
500	1000	62.5	51	49
500	1000	125	26	74
500	1000	250	10	90
250	2000	62.5	1	99
250	2000	125	0	100
250	2000	250	1	99
500	2000	62.5	0	100
500	2000	125	0	100
500	2000	250	0	100

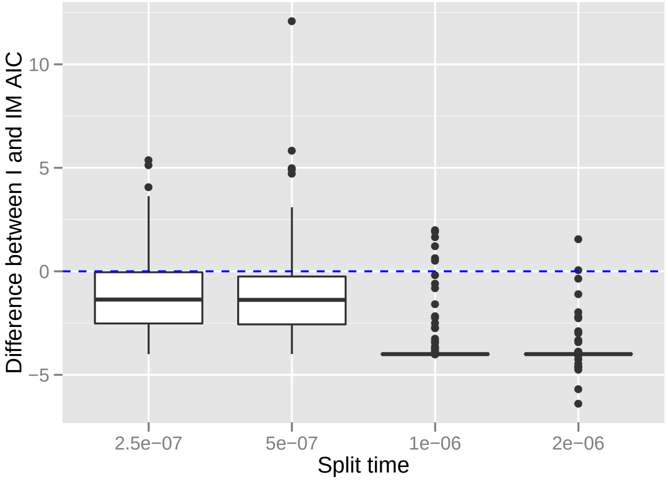

(a) Simulations under the I model

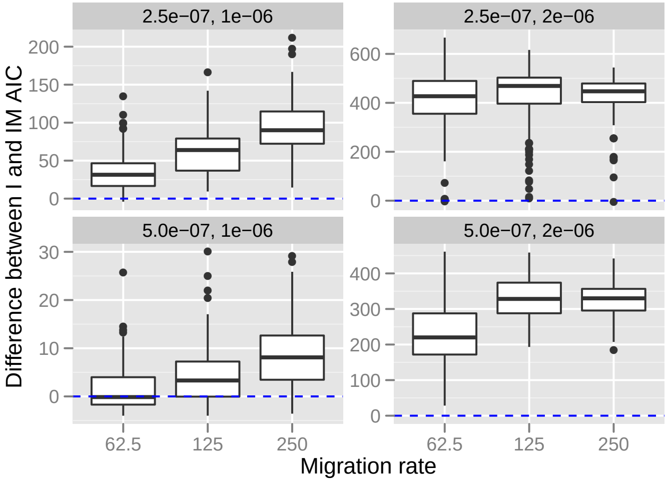

(b) Simulations under the IM model

Figure 38. AIC tests for migration. The plots show AIC for the isolation model minus AIC for the isolation-with-migration model. Values above zero means that the migration model is preferred. In (a) values are shown for simulations under the isolation model, with varying τ, while in (b) values are shown for the simulation model with τ_1 varying between 250 kya and 500 kya (assuming $\mu = 10^{-9}$), τ_2 varying between 1 mya and 2 mya, and M varying between 62.5, 125 and 250.

9 Older speciation times

We performed all the simulation studies with speciation times within the last two million years, which is the time period where we expect the speciations within the great ape genera to have occurred. While the model is likely to fail when considering very old splits, where the variation in divergence along the genome is insignificant compared to the divergence time between species, we *do* expect the model to be applicable further back in time than the last few million years.

We performed a few simulations to validate this, simulating a split time at 6 Mya with or without gene-flow continuing till 4 Mya. We analysed both isolation and isolation-with-migration data with both the isolation and the isolation-with-migration model to see the parameter estimation accuracy both when analysing with the correct and the incorrect model. Results are shown in Fig. 39.

When the analysis model matches the simulation model, the results for this deeper split are as we would expect from the results at more recent splits: The time parameters, migration rate and coalescence rate are reasonably well received, while the recombination rate is underestimated. When an isolation-with-migration model is used to analyse data simulated under a clean isolation model, it estimates a small migration rate and a short migration interval, while if an isolation model is used to analyse data simulated under an isolation-with-migration model the main effects seem to be estimating a more recent split and a smaller coalescence rate (consistent with seeing a larger variance, and thus N_e, in the coalescence times in the ancestral species).

For model checking, the situation is also similar to data with a more recent split time, see Fig. 40. When data is simulated under the isolation model, the AIC slightly prefers the isolation model (the difference in AIC is negative), while for data simulated under the isolation-with-migration model, that AIC difference prefers the IM model.

Since many estimates of the effective population size for older splits among the great apes see larger effective population sizes there, we also ran simulations with a smaller C (in this case using only an isolation model for faster computations). Results are shown in Figure 41. We do not see any noticeable different on estimation accuracy on C or T between these combinations.

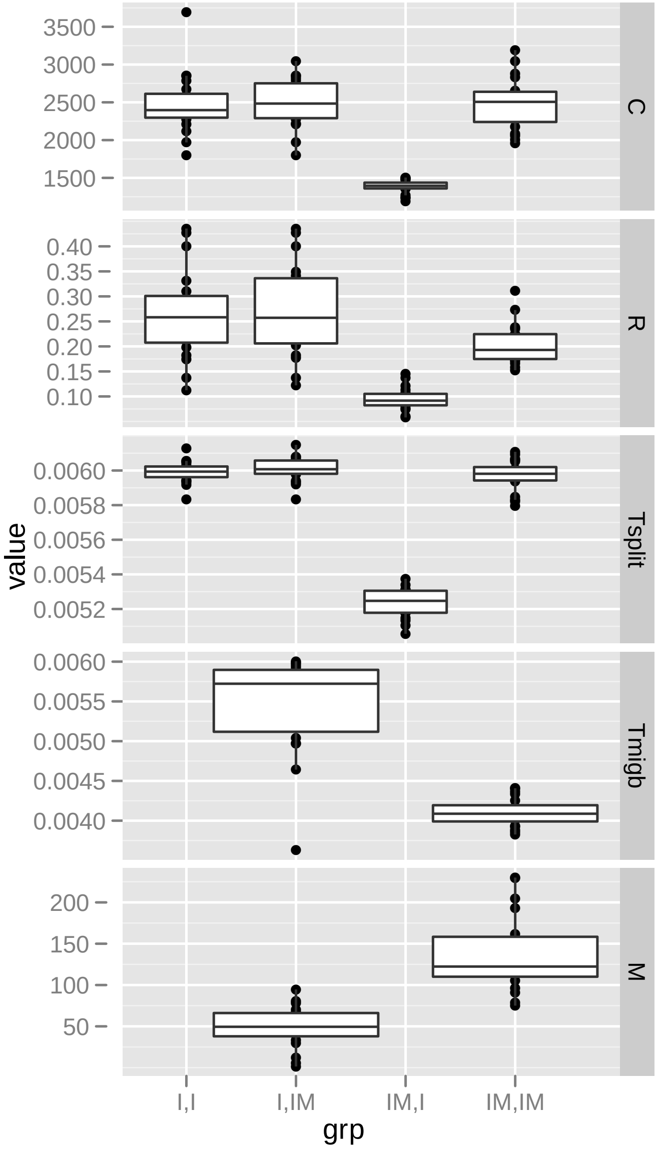

Figure 39. Estimation accuracy for deeper speciation times. Box plots show parameter estimates when the speciation time was simulated to be 6 Mya (with gene-flow continuing until 4 Mya for the IM model). The "groups" (grp) on the x-axis denote the combinations of simulated versus analysis model, so I,I indicates that the data was simulated under an isolation model and analysed under the isolation model as well, while I,IM means that it was simulated under an isolation model but analysed using the isolation with migration model, and so on. Simulated parameters were coalescence rate $C = 2500$, recombination rate $R = 0.40$, split time (Tsplit) 0.006, and for the IM model the end of gene-flow (Tmigb) 0.004 and migration rate $M = 125$.

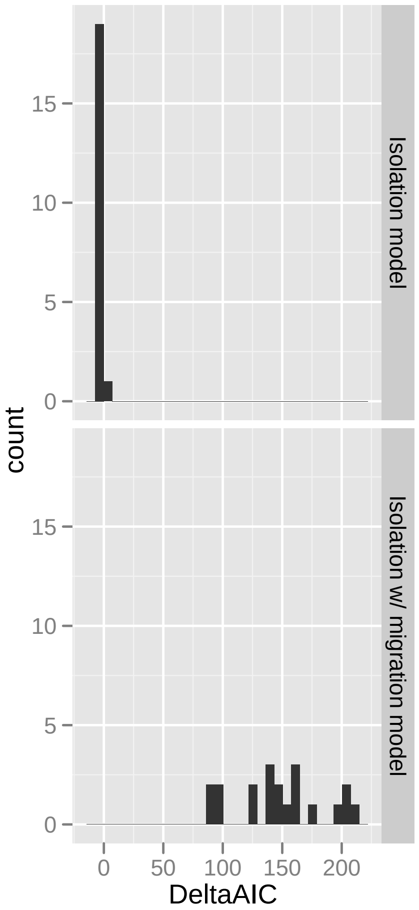

Figure 40. Model checking for deeper speciation times. Histograms of the differences in AIC for the isolation versus the isolation-with-migration model for a split time at 6 Mya.

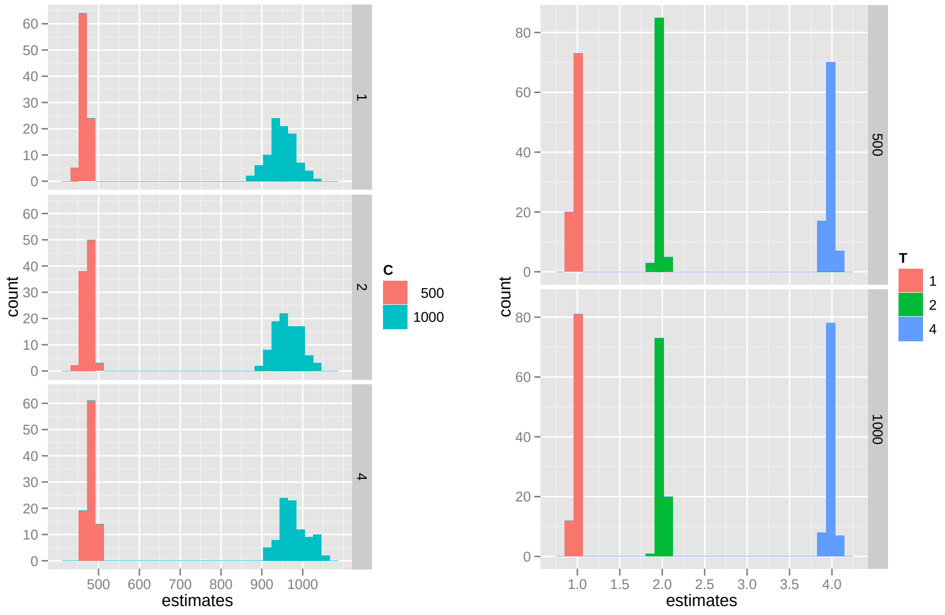

(a) C estimates

(b) T estimates

Figure 41. Varying split time and coalescence rate for older splits Histograms of estimates of C (left) and split time (right) for older split when varying the split time as 1 Mya, 2 Mya and 4 Mya and C as 500 or 1000.

10 Posterior decoding

From the hidden Markov model it is possible to not only estimate parameters, but also make predictions about the coalescence time interval for each nucleotide in an alignment. We do this by computing the posterior probability of being in any of the coalescence time intervals, for each alignment position. In the following, to easy visualization, we show analysis with only four states in the migration interval and four states in the ancestral population, although we do not recommend using so few states for parameter estimation.

Figure 42 plots the mean posterior probability for each state, conditional on the true state, as box plots based on 10 simulated datasets. If there was no information about the true state in the posterior decoding, we would expect the box plots to be similar for all (true) states, reflecting only the prior probabilities of the model. This is clearly not the case. However, there does not appear to be a very strong signal for the true state in the posterior probabilities either. If this was the case, we would expect the HMM state corresponding to the simulated coalescence time interval, to have a high posterior probability and all other states to have a low posterior probability. While each state does have a higher posterior probability when it matches the true state, in general neighboring states tends to have similar posteriors. For the most recent and most ancient time intervals, there does appear some differences that means that we might be able to distinguish those, but the middle intervals do not appear to be distinguishable from each other.

These plots, however, do not capture the spatial patterns of coalescence times and posteriors; they show us the mean posterior of a state over all nucleotide position in the data (with the true coalescence time in the relevant time interval). They thus give the same weight to positions just around a recombination point that changes the coalescence time as they give to positions in the center of regions with the same coalescence time. We would expect this to matter, however. Analyzing closely related species, as is the intention with the model we are developing here, we expect few substitutions along the alignment and extract information for the model from how these sites are distributed along the alignment.

Only when a segment coalesce very recently do we expect it to be both long and with few substitutions, explaining why we are better at predicting the most recent time intervals. At the most ancient coalescence times we find substitutions close together, something we don't expect at more recent coalescence intervals. These intervals are expected to be short, however, most likely the high main posterior is dominated by a few intervals where we do have such close substitutions, while for many ancient coalescence times we see one or zero substitutions in which case the most ancient state is unlikely to have a high posterior probability.

For a more global view at the posterior decoding, we looked at the correlation between the true coalescence times and inferred coalescence times from posterior decoding. Taking the mean coalescence time in the maximum posterior interval as the predicted coalescence time, we found a correlation of 0.68 between predicted and true coalescence time. Since we cannot predict continuous coalescence times the best possible correlation is not 1 but the correlation between true time and the mean of the interval it falls within, which we found to be 0.94.

One way of predicting coalescence times, taking the uncertainty of the posterior decoding into account, is to weigh the predicted coalescence times with their posterior. Let m_i denote the mean coalescence time in interval/state i and let p_i denote the posterior probability of being in state i. The we can use $\sum_i m_i p_i$ as our prediction for the coalescence time for each alignment position. Figure 43 show the correlation between predicted values and true values with this approach.

As the focus on this paper is on using the HMM to estimate parameters of the IM model, not analyzing the posterior decoding of the HMM, we did not explore posterior decoding prediction further.

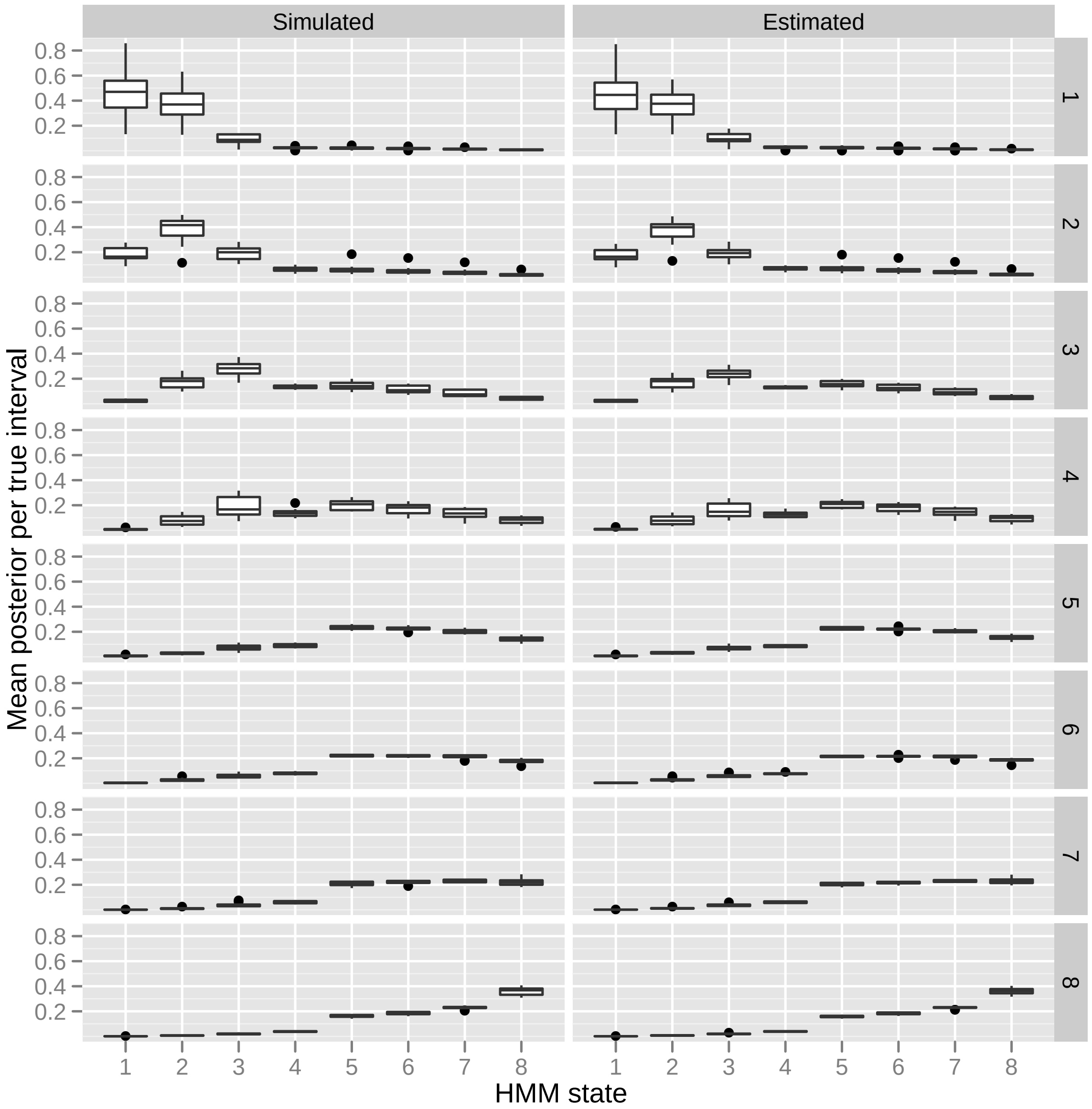

Figure 42. Mean posterior probability for each HMM state for each true state. Boxplots (based on 10 simulated datasets) of the posterior probability for each HMM state (x-axis) versus the true coalescence interval (y-axis). Posteriors were calculated in a model where all parameters were set to their simulated value (on the left) or estimated from the data (on the right). The HMM used here has four states, state 1 to 4, in the gene flow interval, and four states, state 5 to 8, in the ancestral population.

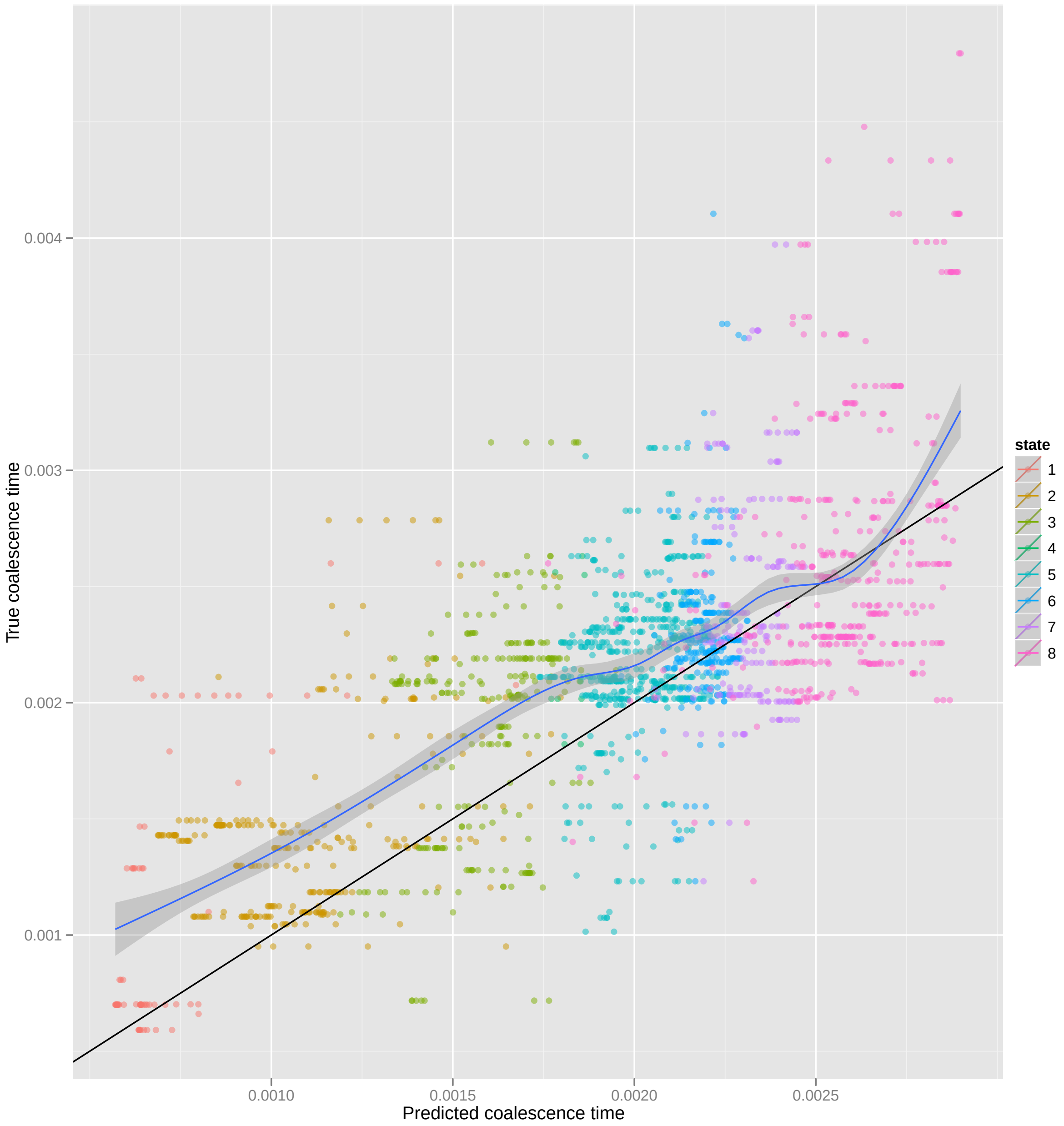

Figure 43. Predicted versus true coalescence times. Predicted coalescence times – mean coalescence times per interval weighted by the posterior probability of the intervals – versus true coalescence times. To avoid over-plotting only every 100th point is plotted. The points are colored according to their maximum posterior state. The black lines shows the $x = y$ line while the blue line shows the best fit of predicted versus true values.

References

1. Mailund T, Schierup MH, Pedersen CNS, Mechlenborg PJM, Madsen JN, et al. (2005) CoaSim: a flexible environment for simulating genetic data under coalescent models. BMC Bioinformatics 6: 252.

2. Dutheil J, Boussau B (2008) Non-homogeneous models of sequence evolution in the Bio++ suite of libraries and programs. BMC Evol Biol 8: 255.

3. Mailund T, Dutheil JY, Hobolth A, Lunter G, Schierup MH (2011) Estimating divergence time and ancestral effective population size of bornean and sumatran orangutan subspecies using a coalescent hidden markov model. PLoS Genet 7.

4. Sand A, Brask AT, Pedersen CNS, Mailund T (2010) HMMlib: A C++ library for general hidden Markov models exploiting modern CPUs. In: Proceedings of HiBi 2010.
