# Supplementary material for: A New Isolation with Migration Model along Complete Genomes Infers Very Different Divergence Processes among Closely Related Great Ape Species
Source: PLoS Genet. 2012 Dec 20;8(12):e1003125. doi: 10.1371/journal.pgen.1003125 (PMC3527290; doi:10.1371/journal.pgen.1003125)
Supplement: Text S2 — Report describing data analysis results not included in the main manuscript. (PDF) [file pgen.1003125.s004.pdf]

Supplemental Text 2: Additional analyses

Thomas Mailund[1,*], Anders E. Halager[1], Asger Hobolth[1], and Mikkel H. Schierup[1,2]
1 Bioinformatics Research Center, Aarhus University, Aarhus, Denmark
2 Department of Biology, Aarhus University, Aarhus, Denmark
∗ **E-mail: mailund@birc.au.dk**

1 Background selection

McVicker *et al.* [1] showed that background selection is widespread in great ape genomes. We would expect background selection to locally decrease the effective population size (i.e. increase the local coalescence rate) but otherwise not affect our model parameters. Further, we wouldn't necessarily expect this to be seen at the 10 Mbp scale where we estimate parameters.

To test this we downloaded the genome wide B statistics from the McVicker *et al.* paper and compared with the human/chimpanzee/bonobo alignment where we have the alignment in hg18 coordinates similar to the B statistics. We plotted estimates for each 10 Mbp chunk against the mean B value for the 10 Mbp chunks (see Figures 1 and 2 for the isolation model and isolation-with-migration model, respectively, for the bonobo-chimpanzee split; the results against human are similar).

We do not expect these parameters to be completely independent, and they are not, but the correlation is very weak.

We would expect that background selection instead would affect the local coalescence time within the segments, something that might be gleaned from posterior decoding of the HMM states, and in one 10 Mbp segment we explored. We computed the expected time to the most recent common ancestor (TMRCA) as $\sum_i m_i p_i$ (see Supplemental Text S1 Section 10) and saw that the mean coalescence time in bins of B values increases with the B value, as expected since high B values indicates neutrality which means higher N_e compared to contained regions (see Figure 3). However, full exploration of how to make inference of such patterns from posterior decoding we find beyond the scope of this paper, but an interesting avenue for future research.

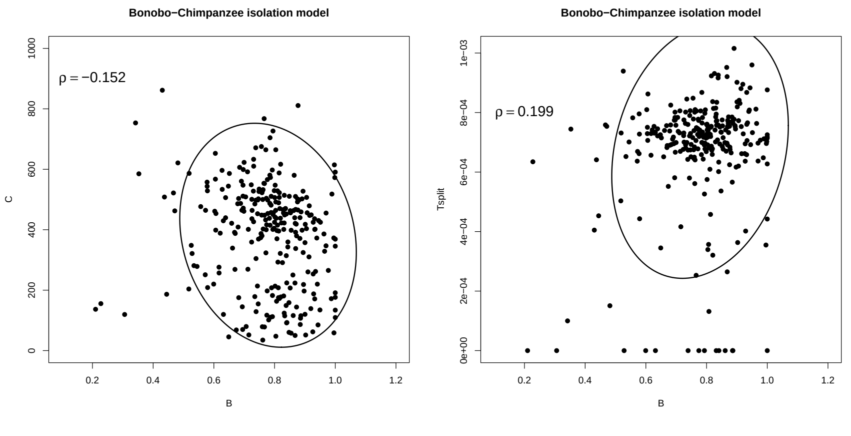

Figure 1. Correlation between split time/coalescence rates and B for the isolation model. The figure shows the correlation between the coalescence rate and the mean B value (left) and the correlation between the split time and mean B value (right). Ellipses show the 95% probability mass for a normal distribution fitted to the data.

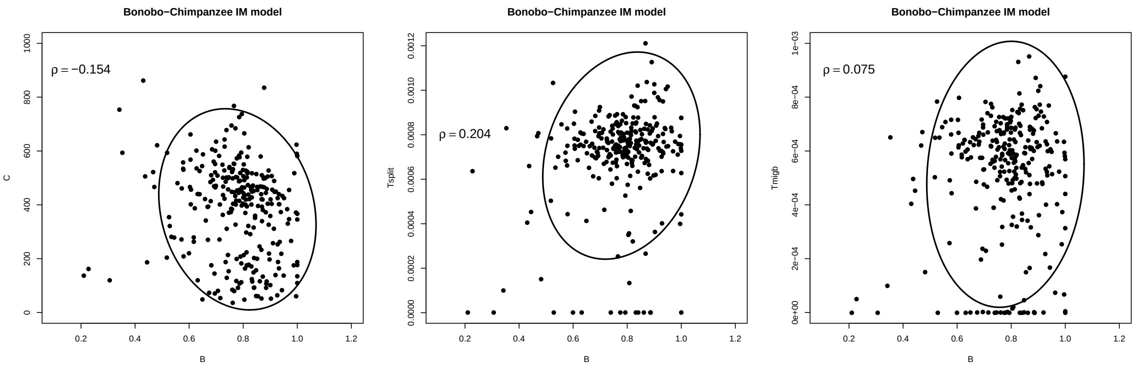

Figure 2. Correlation between split time/coalescence rates and B for the isolation-with-migration model. The figure shows the correlation between the coalescence rate and the mean B value (left) and the correlation between the split time and mean B value (middle and right). Ellipses show the 95% probability mass for a normal distribution fitted to the data.

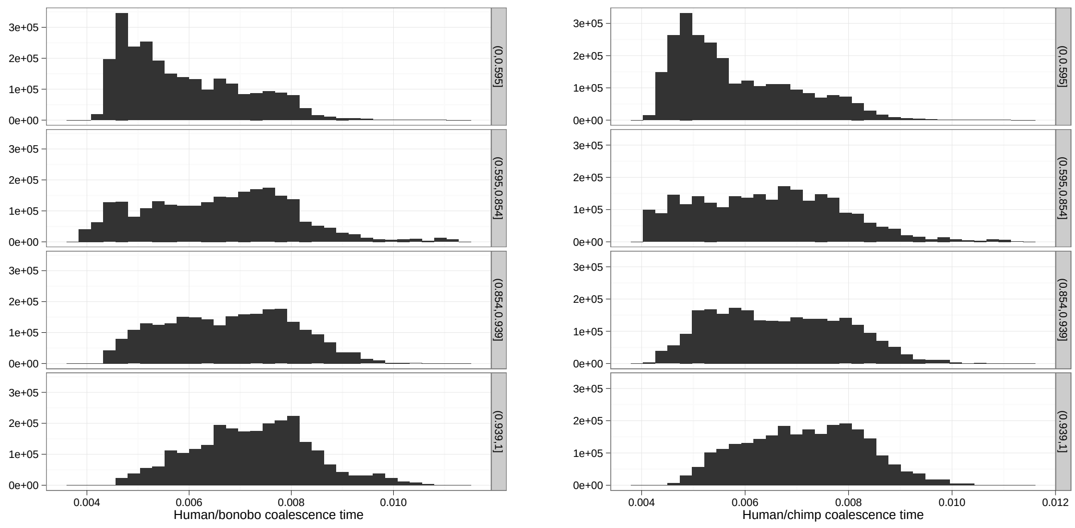

(a) Human/bonobo

(b) Human/chimpanzee

Figure 3. Correlation between B and the local N_e. We split one 10 Mbp chunk into four bins based on the B statistics assigned to each site in the alignment. We then plot the histogram of predicted coalescence times (TMRCA) in each of the four bins. The histograms reflect the local effective population size in the sense that higher values indicate deeper coalescences and thus higher local N_e.

2 Effects of filtering the alignment

Assembly and alignment errors can potentiality lead our model to make incorrect parameter estimates and conclusions about the mode of speciation. Repetitive parts of the genome are more likely to contain assembly or alignment artefacts, so to test if our results are likely to be influenced by this we compared results with the full genome to results where we masked repeats for the human/bonobo and human/chimpanzee results (where we immediately had repeat masks in the alignment).

Effects on parameter estimates are shown in Figures 4 and 5 and results for AIC model checking are shown in Figure 6. The main effect of including repetitive parts of the genome appears to be a slightly smaller coalescence rate (and consequently larger M/C) plus a slight increase in the split time.

The decrease in coalescence rate is expected even *without* alignment artefacts since selection effects – enriched for in non-repetitive areas – would have a smaller effective population size. Alignment artefacts would increase the variance in divergence along the genome which is also consistent with the reduction in C. Still, the contrast between results with and without masked repeats does not appear drastic.

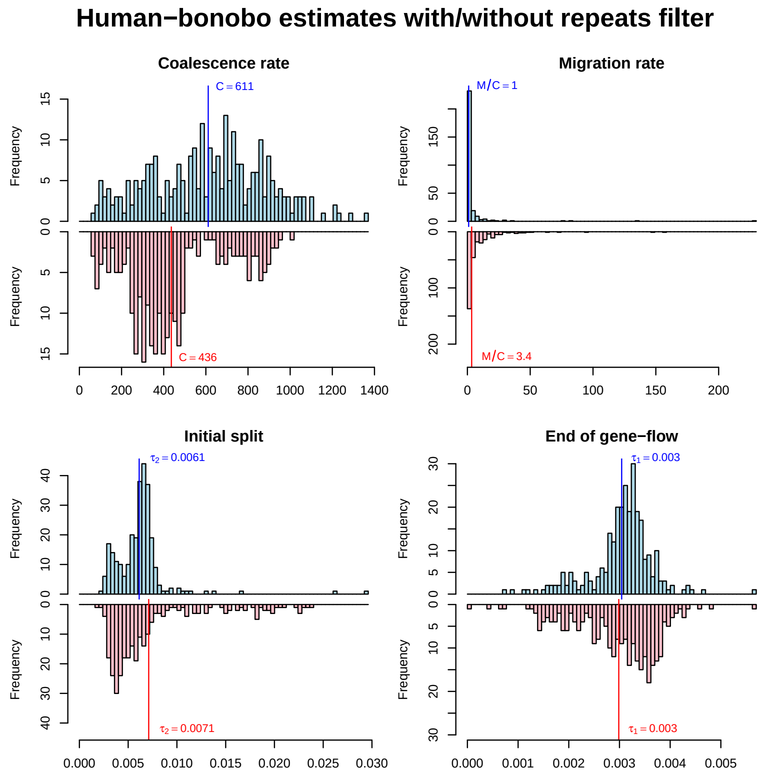

Figure 4. Human/bonobo estimates with and without repeat filter. Top most (blue) histograms show parameter estimates for data where repeats are filtered out while bottom most (red) histogram show estimates when repeats are included.

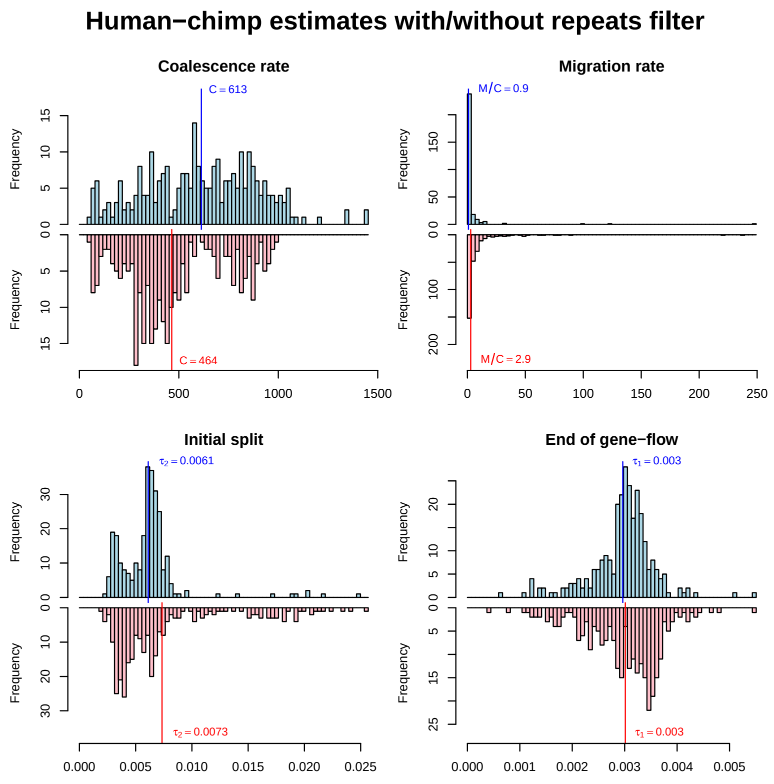

Figure 5. Human/chimpanzee estimates with and without repeat filter. Top most (blue) histograms show parameter estimates for data where repeats are filtered out while bottom most (red) histogram show estimates when repeats are included.

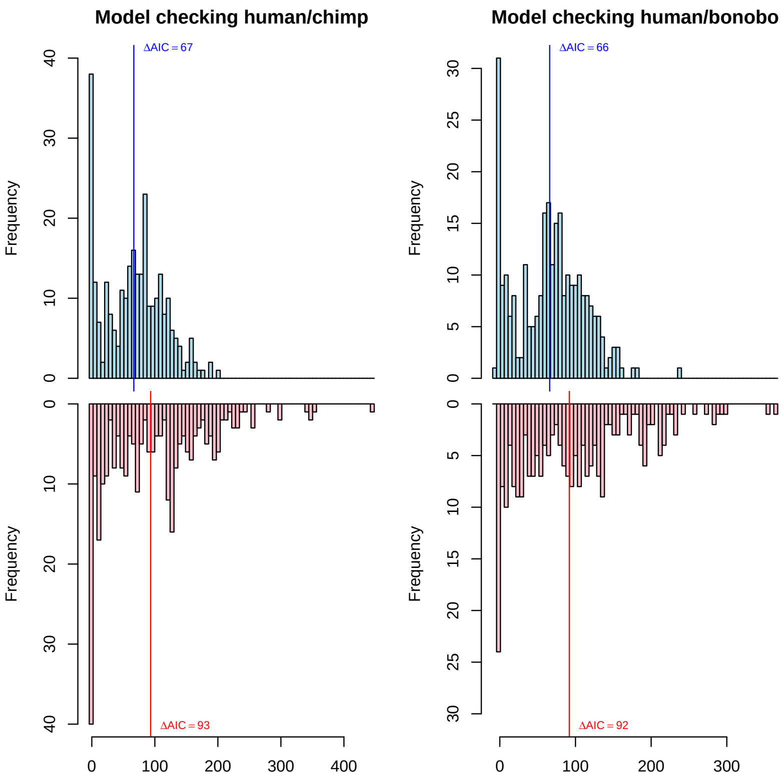

Figure 6. AIC differences with and without repeats masking. Top most (blue) histograms show parameter estimates for data where repeats are filtered out while bottom most (red) histogram show estimates when repeats are included.

3 Comparisons with previous estimates

3.1 Bonobo/chimpanzee split

Our estimate for the chimpanzee/bonobo split, scaled with different mutation rates, is shown in Figure 7, together with point estimates from the literature.

Won and Hey (2005) [2] scaled the mutation rate assuming a divergence between human and chimpanzees of 7 Mya, scaling roughly to $\mu = 0.86 \times 10^{-9}\,\mathrm{bp}^{-1}\,\mathrm{y}^{-1}$. Comparing bonobos and western chimpanzees they obtained a mode estimate (and 95% CI) of 859 kya [589–1309 kya] and comparing bonobos and central chimpanzees they obtained 890 kya [638–1332 kya].

Becquet and Przeworski (2007) [3] scaled the mutation rate assuming a generation time of 20 years per generation and a per-generation mutation rate of 2×10^{-8} corresponding to $\mu = 1.0 \times 10^{-9}\,\mathrm{bp}^{-1}\,\mathrm{y}^{-1}$. Depending on which chimpanzee subspecies they used in their analysis they obtained mode estimates (and 95% CI) of 873 kya [681–1070 kya] (bonobo/western), 918 kya [759–1170 kya] (bonobo/central) and 785 kya [616–1350 kya] (bonobo/eastern).

Caswell *et al.* (2008) [4] scaled the mutation rate assuming a human/chimpanzee divergence of 6 Mya corresponding roughly to $\mu = 1.0 \times 10^{-9}\,\mathrm{bp}^{-1}\,\mathrm{y}^{-1}$, obtaining a mode estimate (and 90% CI) of 1.29 Mya [1.14–1.45 Mya].

Hey (2010) [5] used the same scaling as Won and Hey (2005) obtaining for pairwise comparisons

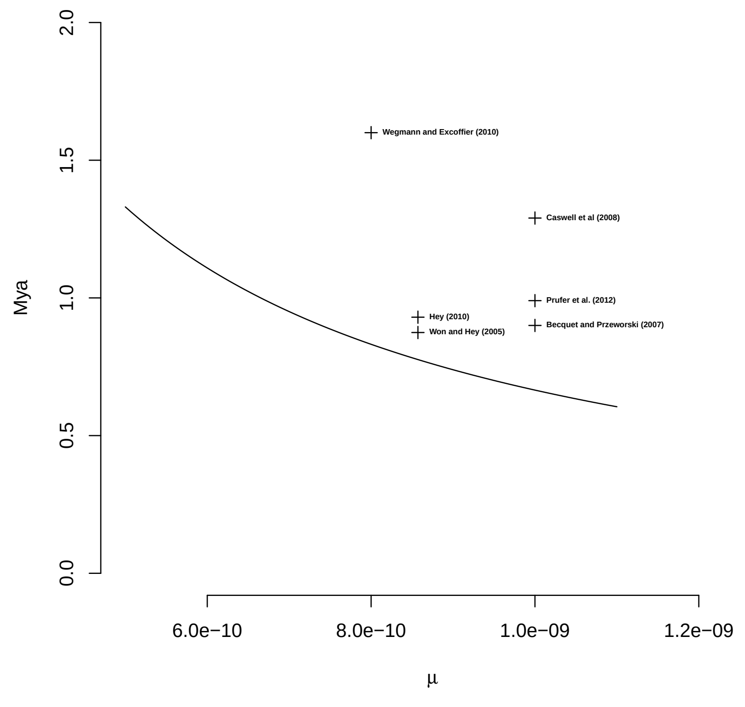

Figure 7. Scaled split time between chimpanzees and bonobos. The line shows our estimate scaled with different mutation rates. Crosses show estimates from previous studies.

estimates of 730 kya [491–1177 kya] (bonobo/eastern), 936 kya [683–1275 kya] (bonobo/central), 845 kya [621–1240 kya] (bonobo/western) and combining all genomes: 930 kya [680–1540 kya].

Wegmann and Excoffier (2010) [6] used a scaling of 1.6×10^{-8} per generation and a generation time of 20 years per generation, corresponding to $\mu = 0.8 \times 10^{-9}\,\text{bp}^{-1}\,\text{y}^{-1}$, obtaining an estimate of 1.6 Mya [1.0–2.0 Mya].

3.2 Eastern and western gorilla split

Our estimate for the gene-flow period for the speciation of the gorillas, for different mutation rates, is shown in Figure 8.

Thalmann *et al.* [7] used a per-generation mutation rate of 1.44×10^{-8} and a generation time of 15 years per generation corresponding to $\mu = 0.96 \times 10^{-9}\,\text{bp}^{-1}\,\text{y}^{-1}$. They used two different methods to estimate the split: MDS (seeing an initial split 0.9-1.05 Mya and an end of gene-flow 164–230 kya) and IM (with gene-flow to the present where they saw two modes, one at 1.6 Mya and one at 77 kya).

Becquet and Przeworski (2007) [3] scaled the mutation rate assuming a generation time of 15 years per generation and a per-generation mutation rate of 2×10^{-8} corresponding to $\mu = 1.3 \times 10^{-9}\,\text{bp}^{-1}\,\text{y}^{-1}$, and estimated an initial split at 91 kya [84–1440 kya] with gene flow to the present.

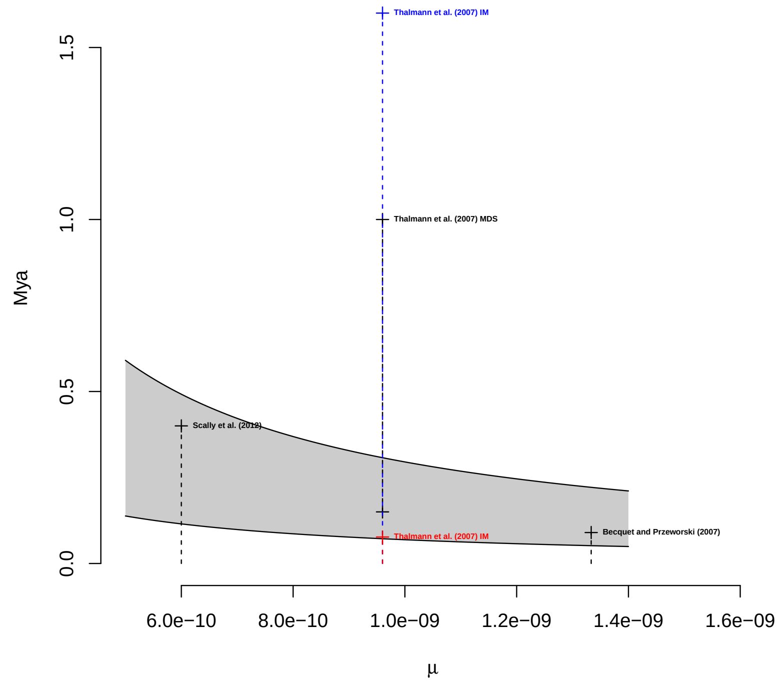

Figure 8. Scaled split time between eastern and western gorilla. The shaded area shows our estimate scaled with different mutation rates. Dashed lines show migration intervals estimated, with a cross at the initial population split (and with a cross at the bottom where it does not extend all the way down to the present).

Scally *et al.* (2012) [8] used a mutation rate of $\mu = 0.6 \times 10^{-9}\,\mathrm{bp}^{-1}\,\mathrm{y}^{-1}$ and saw an initial population split at 0.5 Mya with moderate gene flow to the present.

3.3 Bornean and Sumatran orang-utan split

Our estimate for the gene-flow period for the speciation of the orang-utans, for different mutation rates, is shown in Figure 9.

Becquet and Przeworski (2007) [3] scaled the mutation rate assuming a generation time of 20 years per generation and a per-generation mutation rate of 2×10^{-8} corresponding to $\mu = 1.0 \times 10^{-9}\,\mathrm{bp}^{-1}\,\mathrm{y}^{-1}$, estimating an initial population split at 1.4 Mya [0.2–1.9 Mya] with gene flow to the present.

Mailund *et al.* (2011) [9], assuming a clean isolation model and a mutation rate of $\mu = 1.0 \times 10^{-9}\,\mathrm{bp}^{-1}\,\mathrm{y}^{-1}$, estimated a split time at 334±154 kya. The split time close to the end of gene flow we estimate with the IM model is consistent with simulation experiments that show that the isolation model is likely to estimate a split time close to the end of gene flow.

Locke *et al.* (2011) [10], using a mutation rate of $\mu = 1.0 \times 10^{-9}\,\mathrm{bp}^{-1}\,\mathrm{y}^{-1}$, estimated a split time at 400 kya with gene flow to the present.

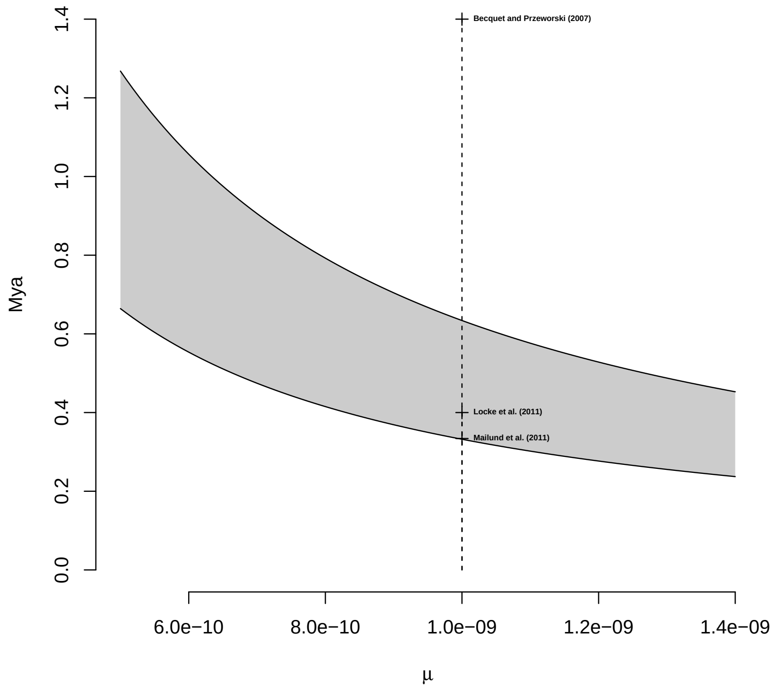

Figure 9. Scaled split time between Sumatran and Bornean orang-utans. The shaded area shows our estimate scaled with different mutation rates. Crosses show point estimates from previous studies while dashed lines show migration intervals estimated (with a cross at the bottom where it does not extend all the way down to the present).

References

1. McVicker G, Gordon D, Davis C, Green P (2009) Widespread genomic signatures of natural selection in hominid evolution. PLoS Genet 5: e1000471.

2. Won YJ, Hey J (2005) Divergence population genetics of chimpanzees. Mol Biol Evol 22: 297–307.

3. Becquet C, Przeworski M (2007) A new approach to estimate parameters of speciation models with application to apes. Genome Res 17: 1505–1519.

4. Caswell JL, Mallick S, Richter DJ, Neubauer J, Schirmer C, et al. (2008) Analysis of chimpanzee history based on genome sequence alignments. PLoS Genet 4: e1000057.

5. Hey J (2010) The divergence of chimpanzee species and subspecies as revealed in multipopulation isolation-with-migration analyses. Mol Biol Evol 27: 921–933.

6. Wegmann D, Excoffier L (2010) Bayesian inference of the demographic history of chimpanzees. Mol Biol Evol 27: 1425–1435.

7. Thalmann O, Fischer A, Lankester F, Pääbo S, Vigilant L (2007) The complex evolutionary history of gorillas: insights from genomic data. Mol Biol Evol 24: 146-58.

8. Scally A, Dutheil JY, Hillier LW, Jordan GE, Goodhead I, et al. (2012) Insights into hominid evolution from the gorilla genome sequence. Nature 483: 169–175.

9. Mailund T, Dutheil JY, Hobolth A, Lunter G, Schierup MH (2011) Estimating divergence time and ancestral effective population size of bornean and sumatran orangutan subspecies using a coalescent hidden markov model. PLoS Genet 7.

10. Locke DP, Hillier LW, Warren WC, Worley KC, Nazareth LV, et al. (2011) Comparative and demographic analysis of orang-utan genomes. Nature 469: 529–533.
